# Supplementary material for: Praja2 controls P-body assembly and translation in glioblastoma by non-proteolytic ubiquitylation of DDX6
Source: EMBO Rep. 2025 Mar 27;26(9):2347–77. doi: 10.1038/s44319-025-00425-5 (PMC12069581; doi:10.1038/s44319-025-00425-5)
Supplement: Supplementary file 1 — Appendix [file 44319_2025_425_MOESM1_ESM.pdf]

Appendix

Table of content

Page 2: **Appendix Figure S1**. Ubiquitylation assays of DDX6 lysin mutants

Page 3: **Appendix Figure S2**. Ubiquitylated lysines identified in DDX6 after stimulation of cells with cAMP

Page 3: **Appendix Figure S3**. praja2 controls mRNA translation and DDX6-mRNA complexes.

Page 4: **Appendix Figure S4**. praja2 deletion induces GBM growth arrest.

Page 5-11: **Appendix Table S1**. Statistical analysis of intersections of significant gene sets from GBM tissues (GSE129671_PW030_701/702, GBM bulk data study in TCGA, PDC00204 proteomic data, GSE67835 Normal tissue) and gene sets from siCNT or sipraja2 U87MG.

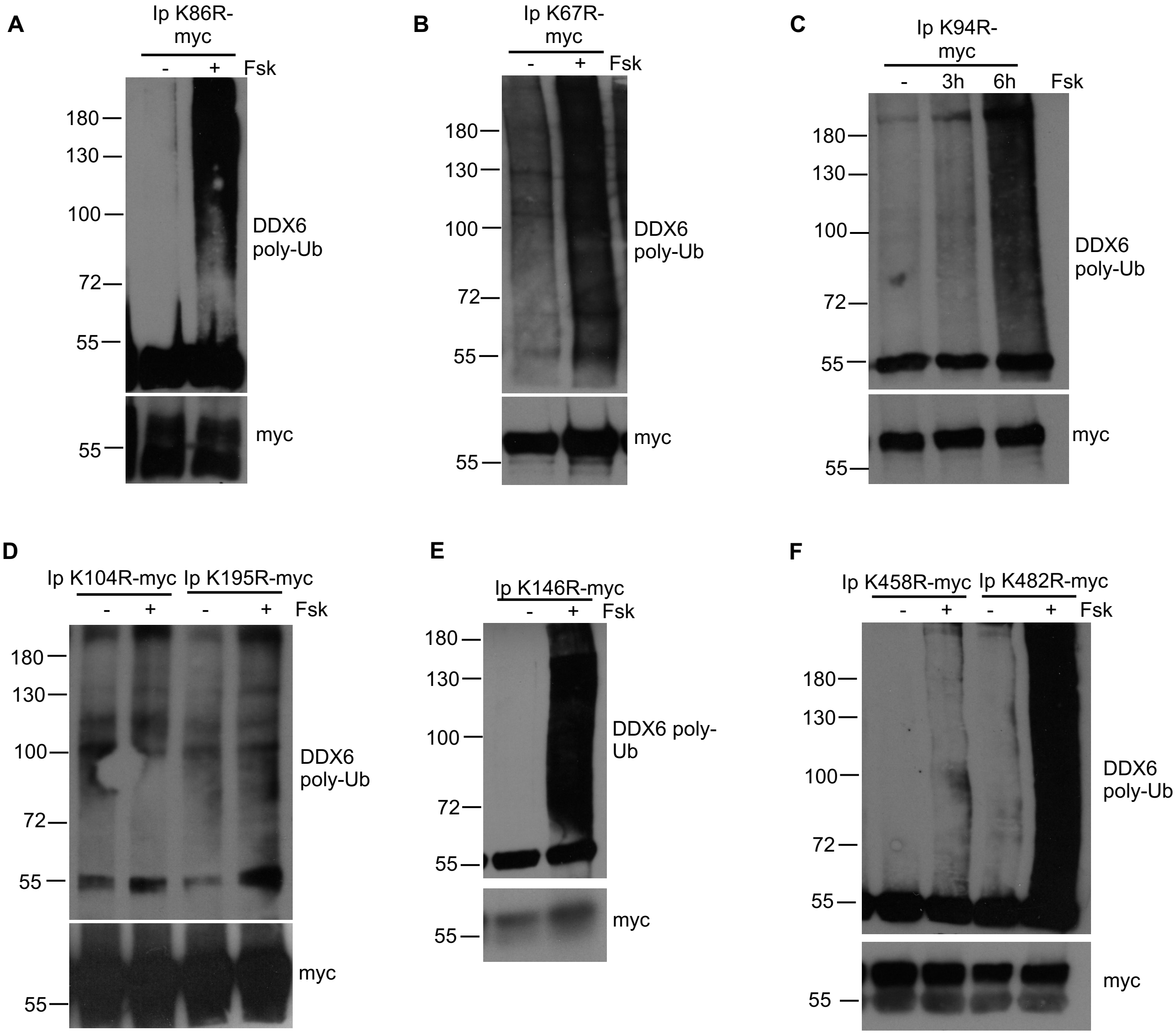

Appendix Figure S1. Ubiquitylation assays of DDX6 lysin mutants. HEK293 cells were co-transfected with Ubiquitin-HA and the indicated mutants of DDX6-myc. Cells were starved 24 hours, pretreated with MG132 (10 μM), treated with Forskolin (20 μM) and immunoprecipitated with anti-myc. Precipitates were immunoblotted with anti-Ub-HA (DDX6 poly-Ub) and anti-myc.

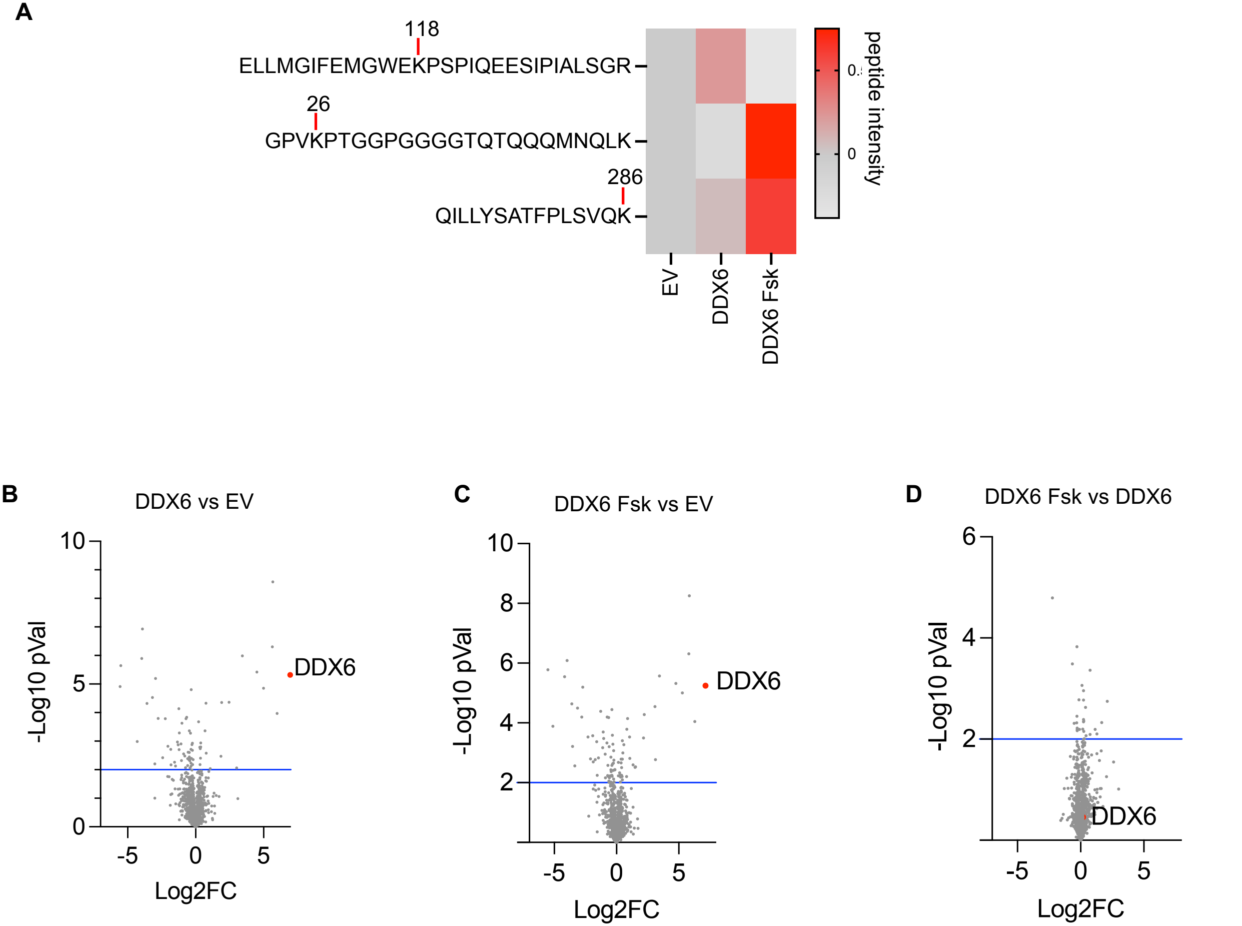

Appendix Figure S2. Ubiquitylated lysines identified in DDX6 after stimulation of cells with cAMP. A. DDX6's ubiquitylated peptides have been identified by LC-MS/MS from immunoprecipitates of empty vector (EV), DDX6, or DDX6 + Forskolin (DDX6 Fsk) , related to approach in B. The heatmap shows the peptide intensity of the three ubiquitylation sites of DDX6 (marked with red lines). **B.** Volcano plot comparing peptide intensity of DDX6 vs empty vector (EV) interacting proteins. Plotted for each protein are the -log10 of Student's t test p-value and log2 fold change (FC) of protein levels. Blue bar = FDR 5%. (n=3 biological independent replicates) **C.** Volcano plot comparing peptide intensity of DDX6+Fsk vs EV interacting proteins. Plotted for each protein are the -log10 of Student's t test p-value and log2FC of protein levels. Blue bar = FDR 5%. (n=3 biological independent replicates) **D.** Volcano plot comparing peptide intensity of DDX6+Fsk vs DDX6 interacting proteins. Plotted for each protein are the -log10 of Student's t test p-value and log2FC of protein levels. Blue bar = FDR 5%. (n=3 biological independent replicates)

A

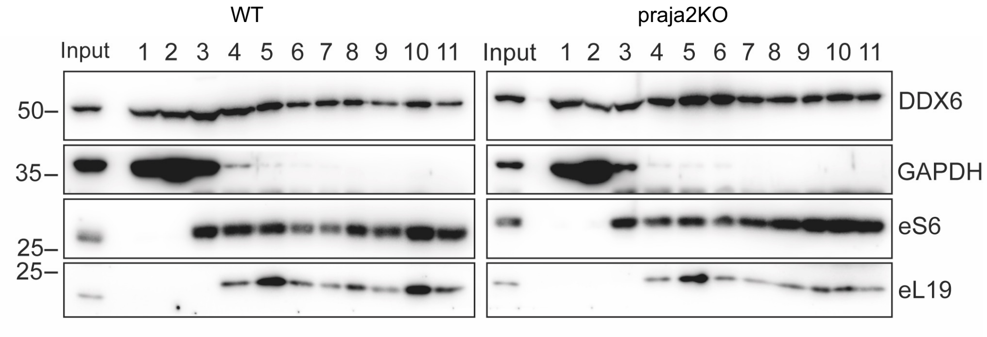

B

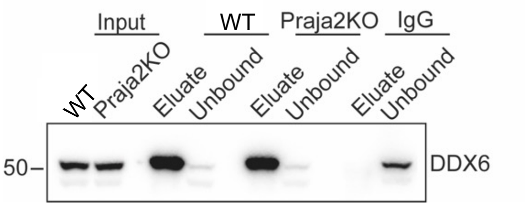

Appendix Figure S3. praja2 controls mRNA translation and DDX6-mRNA complexes. A. Fractions 1 and 2 are free cytosolic proteins or light complexes; fractions from 3 to 6, ribosomal subunits (60S, 40S) and monomer (80S); and fractions from 7 to 11, polysomes. Proteins from each fraction were analyzed by WB with the indicated antibodies. **B**. Quality control of DDX6 IP for RIP-seq experiments. 1% of the input fraction, 10% of the unbound fraction and 10% of DDX6 IP were analyzed by WB with DDX6 antibody.

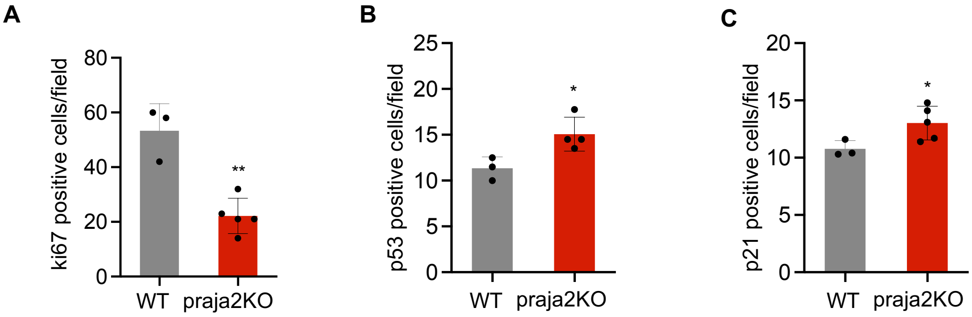

Appendix Figure S4. praja2 deletion induces GBM growth arrest. A. Quantitative analysis of ki67 immunostaining on tissue derived from mice injected with U87MG (WT) or with U87MG praja2KO (praja2KO). For each mouse (3 WT and 5 praja2KO) positive cells from 3 different fields (at 100x, approximatively 100 cells) has been counted. Mean value ± SEM is shown. t test **p=0.0015 **B.** Quantitative analysis of p53 immunostaining on tissue derived from mice injected with U87MG (WT) or with U87MG praja2KO (praja2KO). For each mouse (3 WT and 4 praja2KO) positive cells from 3 different fields (at 100x, approximatively 100 cells) has been counted. Mean value ± SEM is shown. t test *p=0.031 **C.** Quantitative analysis of p21 immunostaining on tissue derived from mice injected with U87MG (WT) or with U87MG praja2KO (praja2KO). For each mouse (3 WT and 5 praja2KO) positive cells from 3 different fields (at 100x, approximatively 100 cells) has been counted. Mean value ± SEM is shown. t test *p=0.028

Appendix Table S1. Statistical analysis of intersections of significant gene sets from GBM tissues: GSE129671 PW030 701/702 (Data ref: Ding *et al*, 2019), GBM bulk data study in TCGA (Data ref: TCGA-GBM, phs000178), PDC00204 proteomic data (Data ref: Wang *et al*, 2021), GSE67835 Normal tissue (Data ref: Darmanis *et al*, 2015) and gene sets from siCNT or sipraja2 U87MG.

The results below show the outputs of the study testing whether the intersections of the U87MG enrichments in Fig. 1C are significant when compared to each of the enrichments in Figs. 1D-H. The hypergeometric (Fisher's exact test) was considered to determine the significance of intersections.

The analysis was run with the function *massiveORT* in the R package massiveGST by replacing the reference gene sets with the names of the gene sets resulting significant in the U87MG experiment. Similarly, these two groups have been compared with the names of the significant gene sets in the enrichments from the different data types.

● Statistical analysis of the intersection of significant gene sets from **GSE129671_PW030_701** donor in **Fig1D** and the two reference groups of gene sets coming from the U87MG genomic profile in Fig.1C.

Hypergeometric test (Fisher's exact test) of gene sets positively correlated with PRAJA2 expression

	collection	universe size	geneList size	geneSet size	geneList in geneSet	odds_ratio	log2 odds_ratio	p.value	BH.value
SiControl	U87MG.gmt	9446	416	345	146	23.9692	4.5831	1.1e-112	2.1e-112
SiPJA2	U87MG.gmt	9446	416	248	13	1.2072	0.2717	3.0e-01	3.0e-01

Hypergeometric test (Fisher's exact test) of gene sets negatively correlated with PRAJA2 expression

	collection	universe size	geneList size	geneSet size	geneList in geneSet	odds_ratio	log2 odds_ratio	p.value	BH.value
SiPJA2	U87MG.gmt	9446	897	248	89	5.8111	2.5388	1.1e-30	2.2e-30
SiControl	U87MG.gmt	9446	897	345	1	0.0266	-5.2303	1.0e+00	1.0e+00

● Statistical analysis of the intersection of significant gene sets from **GSE129671_PW030_702** donor in **Fig1E** and the two reference groups of gene sets coming from the U87MG genomic profile in Fig.1C.

Hypergeometric test (Fisher's exact test) of gene sets positively correlated with PRAJA2 expression

	collection	universe size	geneList size	geneSet size	geneList in geneSet	odds_ratio	log2 odds_ratio	p.value	BH.value
SiControl	U87MG.gmt	9446	302	345	86	13.6474	3.7706	8.7e-55	1.7e-54
SiPJA2	U87MG.gmt	9446	302	248	15	1.9986	0.9990	1.3e-02	1.3e-02

Hypergeometric test (Fisher's exact test) of gene sets negatively correlated with PRAJA2 expression

	collection	universe size	geneList size	geneSet size	geneList in geneSet	odds_ratio	log2 odds_ratio	p.value	BH.value
SiPJA2	U87MG.gmt	9446	855	248	91	6.3954	2.6770	6.6e-34	1.3e-33
SiControl	U87MG.gmt	9446	855	345	3	0.0849	-3.5574	1.0e+00	1.0e+00

● Statistical analysis of the intersection of significant gene sets from **GBM bulk data study in TCGA** in **Fig1F** and the two reference groups of gene sets coming from the U87MG genomic profile in Fig.1C.

Hypergeometric test (Fisher's exact test) of gene sets positively correlated with PRAJA2 expression

	collection	universe size	geneList size	geneSet size	geneList in geneSet	odds_ratio	log2 odds_ratio	p.value	BH.value
SiControl	U87MG.gmt	9446	316	345	137	32.7724	5.0344	4.2e-120	8.5e-120
SiPJA2	U87MG.gmt	9446	316	248	18	2.3370	1.2247	1.7e-03	1.7e-03

Hypergeometric test (Fisher's exact test) of gene sets negatively correlated with PRAJA2 expression

	collection	universe size	geneList size	geneSet size	geneList in geneSet	odds_ratio	log2 odds_ratio	p.value	BH.value
SiPJA2	U87MG.gmt	9446	193	248	28	6.9628	2.7997	1.1e-13	2.2e-13
SiControl	U87MG.gmt	9446	193	345	1	0.1349	-2.8901	1.0e+00	1.0e+00

● Statistical analysis of the intersection of significant gene sets from **PDC00204** proteomic data in **Fig1G** and the two reference groups of gene sets coming from the U87MG genomic profile in Fig.1C.

Hypergeometric test (Fisher's exact test) of gene sets positively correlated with PRAJA2 expression

	collection	universe size	geneList size	geneSet size	geneList in geneSet	odds_ratio	log2 odds_ratio	p.value	BH.value
SiControl	U87MG.gmt	9446	462	345	214	58.1895	5.8627	2.3e-209	4.6e-209
SiPJA2	U87MG.gmt	9446	462	248	2	0.1545	-2.6948	1.0e+00	1.0e+00

Hypergeometric test (Fisher's exact test) of gene sets negatively correlated with PRAJA2 expression

	collection	universe size	geneList size	geneSet size	geneList in geneSet	odds_ratio	log2 odds_ratio	p.value	BH.value
SiPJA2	U87MG.gmt	9446	790	248	96	7.7357	2.9515	4.7e-41	9.4e-41
SiControl	U87MG.gmt	9446	790	345	2	0.0615	-4.0228	1.0e+00	1.0e+00

● Statistical analysis of the intersection of significant gene sets from **GSE67835 Normal tissue** data in **Fig1H** and the two reference groups of gene sets coming from the U87MG genomic profile in Fig.1C.

Hypergeometric test (Fisher's exact test) of gene sets positively correlated with PRAJA2 expression

	collection	universe size	geneList size	geneSet size	geneList in geneSet	odds_ratio	log2 odds_ratio	p.value	BH.value
SiPJA2	U87MG.gmt	9446	5	248	3	56.1632	5.8116	0.00017	0.00034
SiControl	U87MG.gmt	9446	5	345	0	0.0000	-Inf	1.00000	1.00000

Hypergeometric test (Fisher's exact test) of gene sets negatively correlated with PRAJA2 expression

	collection	universe size	geneList size	geneSet size	geneList in geneSet	odds_ratio	log2 odds_ratio	p.value	BH.value
SiPJA2	U87MG.gmt	9446	66	248	23	21.7338	4.4419	2e-20	4e-20
SiControl	U87MG.gmt	9446	66	345	2	0.8234	-0.2804	7e-01	7e-01
