# Supplementary figures and images for: Praja2 controls P-body assembly and translation in glioblastoma by non-proteolytic ubiquitylation of DDX6 (part 1 of 2)
Source: EMBO Rep. 2025 Mar 27;26(9):2347–77. doi: 10.1038/s44319-025-00425-5 (PMC12069581; doi:10.1038/s44319-025-00425-5)

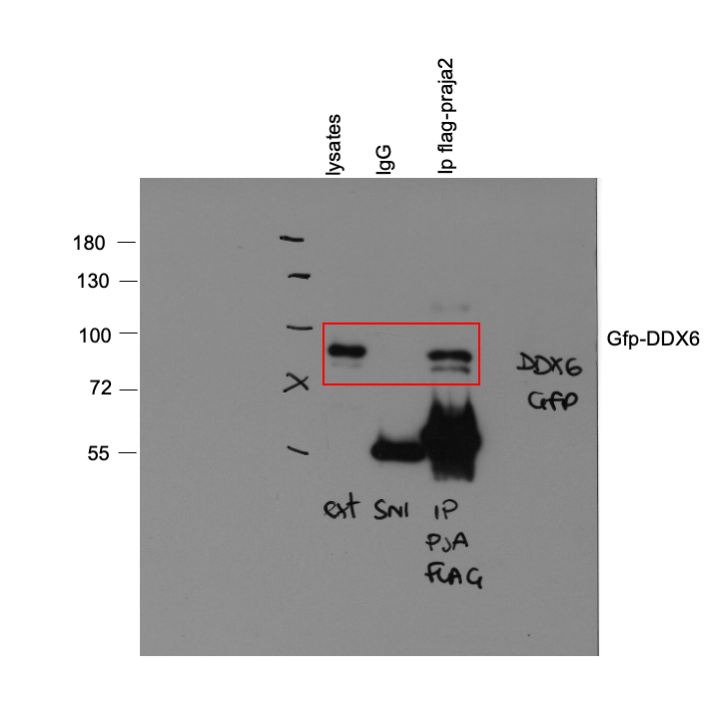

Supplement: Supplementary file 13 — Source data Fig. 3 [file 44319_2025_425_MOESM13_ESM.zip › Figure 3/3E/Gfp-DDX6.tiff]

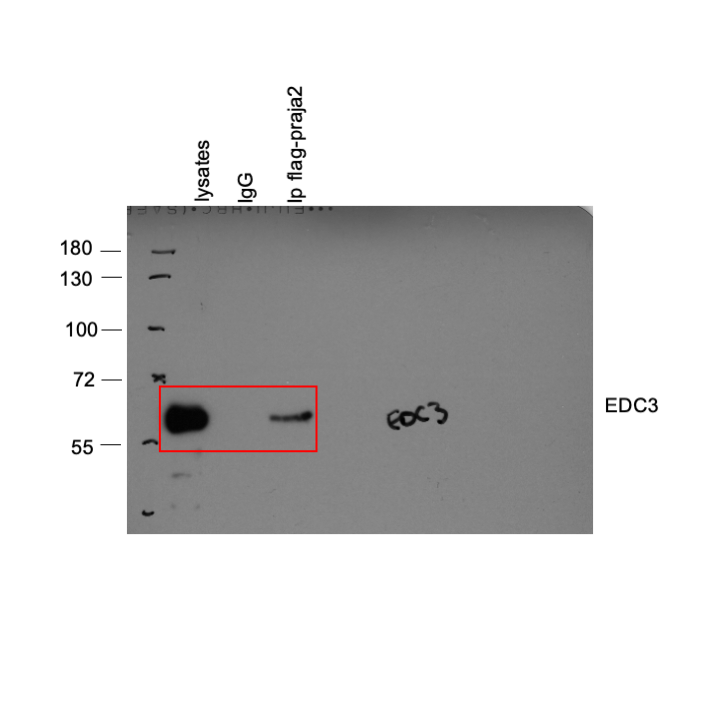

Supplement: Supplementary file 13 — Source data Fig. 3 [file 44319_2025_425_MOESM13_ESM.zip › Figure 3/3E/EDC3.tiff]

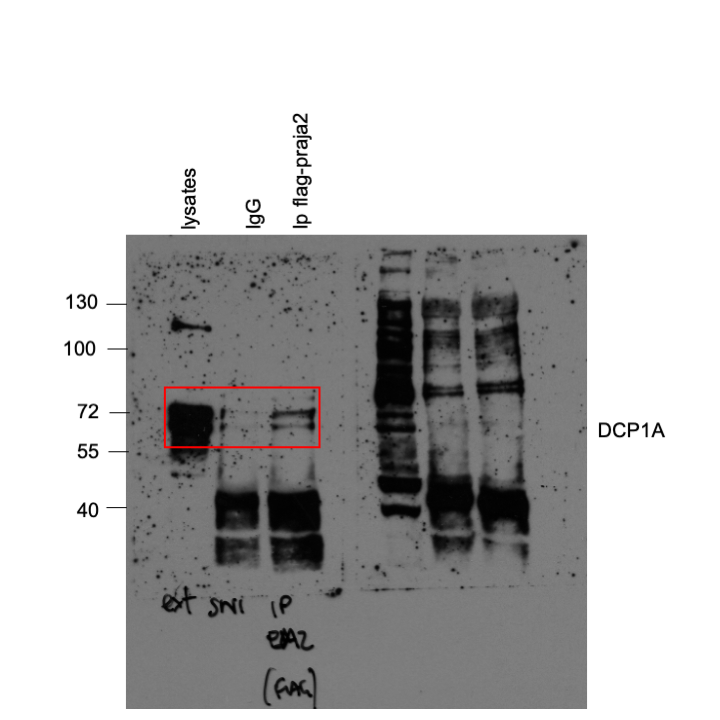

Supplement: Supplementary file 13 — Source data Fig. 3 [file 44319_2025_425_MOESM13_ESM.zip › Figure 3/3E/DCP1A.tiff]

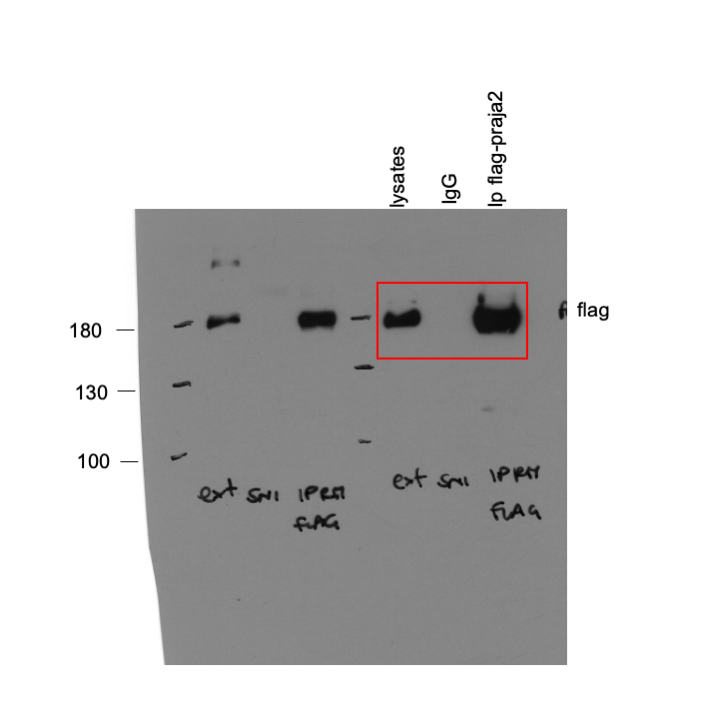

Supplement: Supplementary file 13 — Source data Fig. 3 [file 44319_2025_425_MOESM13_ESM.zip › Figure 3/3E/flag.tiff]

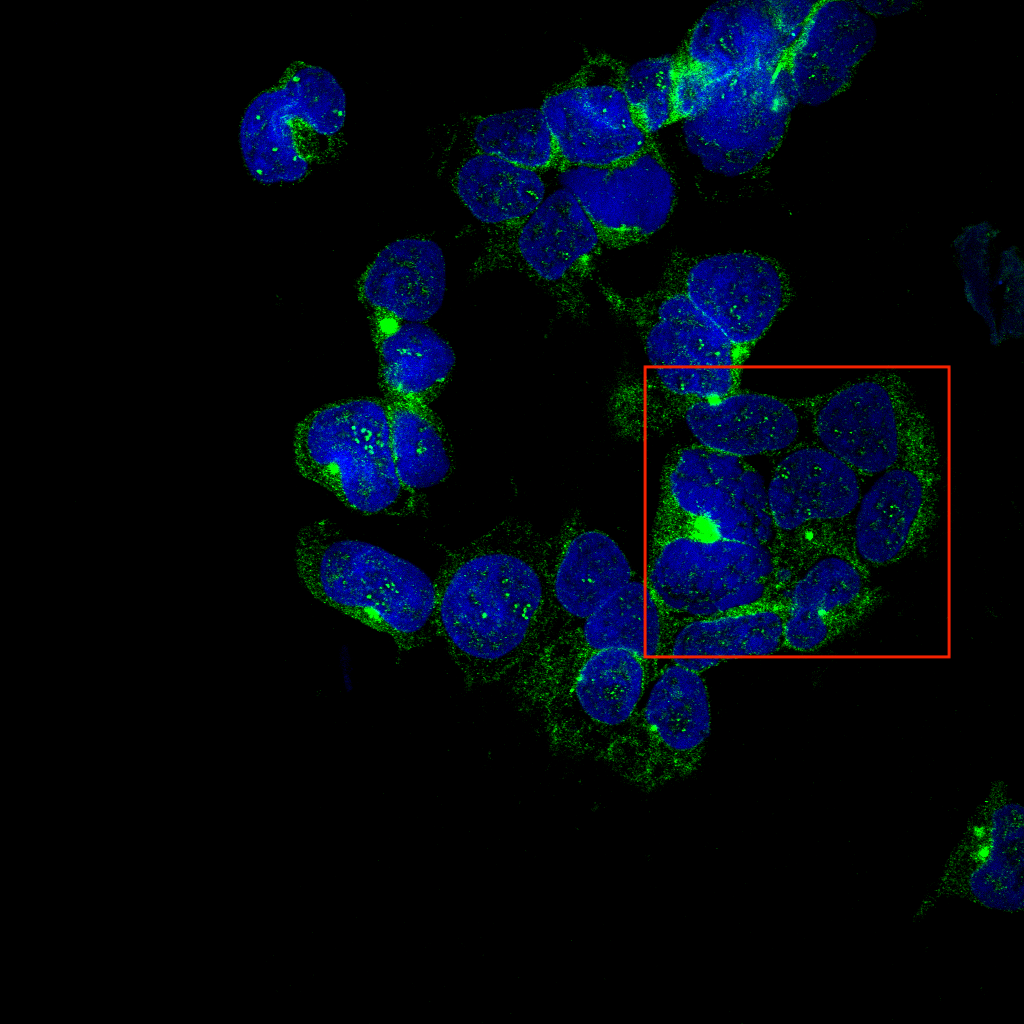

Supplement: Supplementary file 13 — Source data Fig. 3 [file 44319_2025_425_MOESM13_ESM.zip › Figure 3/3B/praja2.tiff]

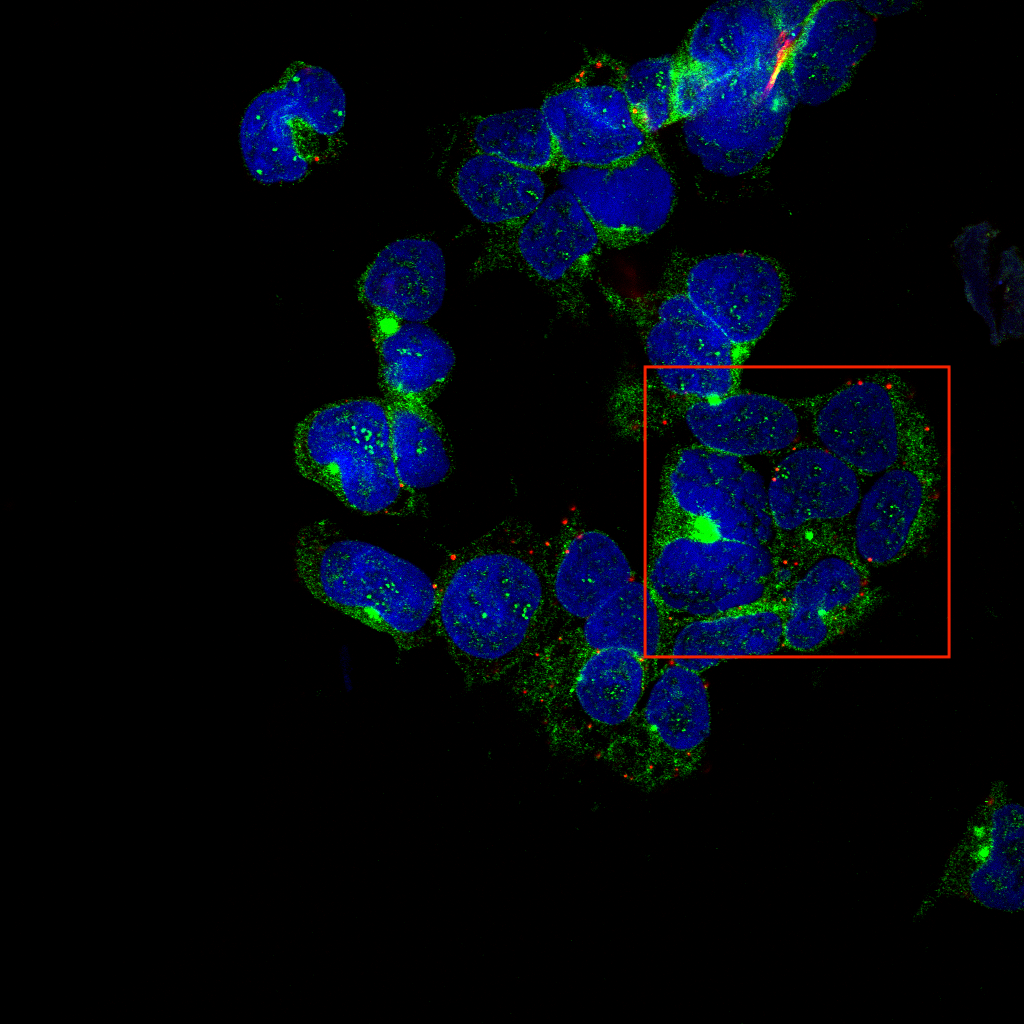

Supplement: Supplementary file 13 — Source data Fig. 3 [file 44319_2025_425_MOESM13_ESM.zip › Figure 3/3B/Merge.tiff]

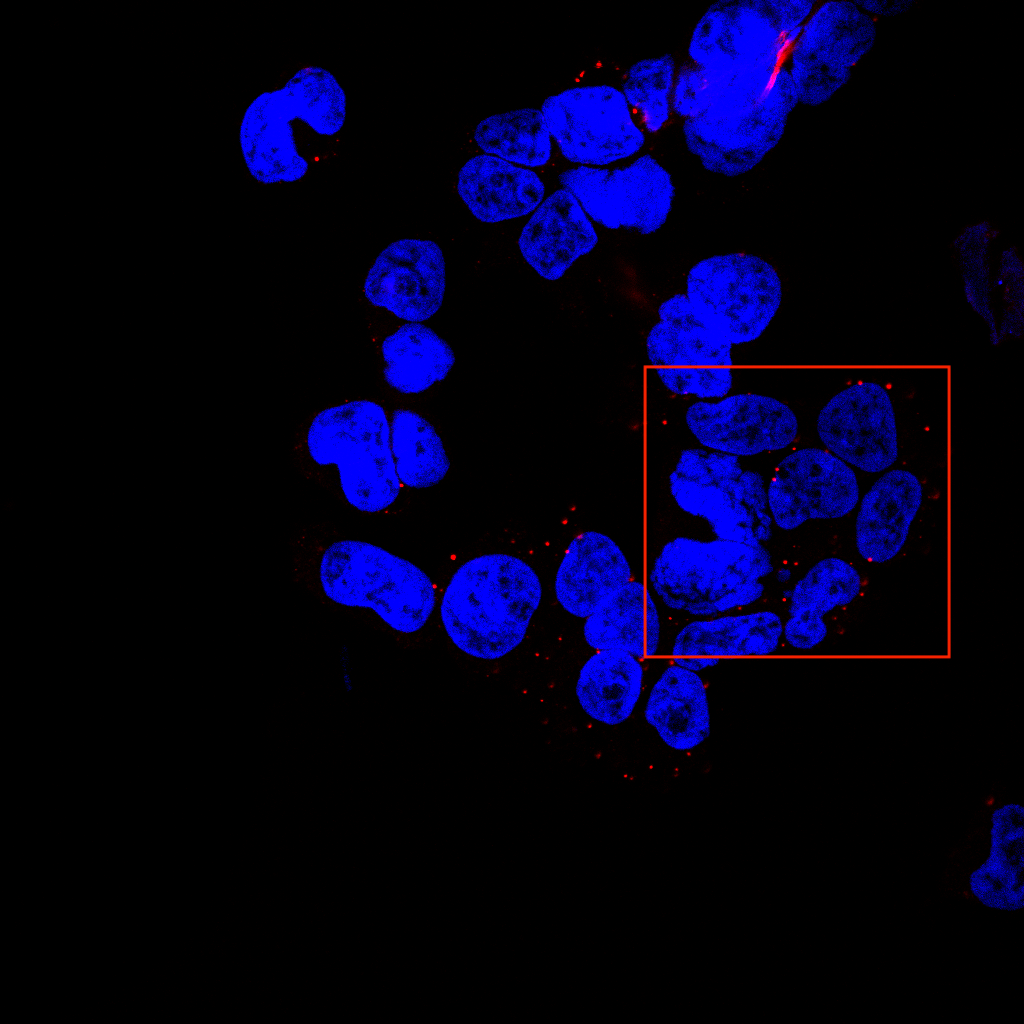

Supplement: Supplementary file 13 — Source data Fig. 3 [file 44319_2025_425_MOESM13_ESM.zip › Figure 3/3B/DDX6.tiff]

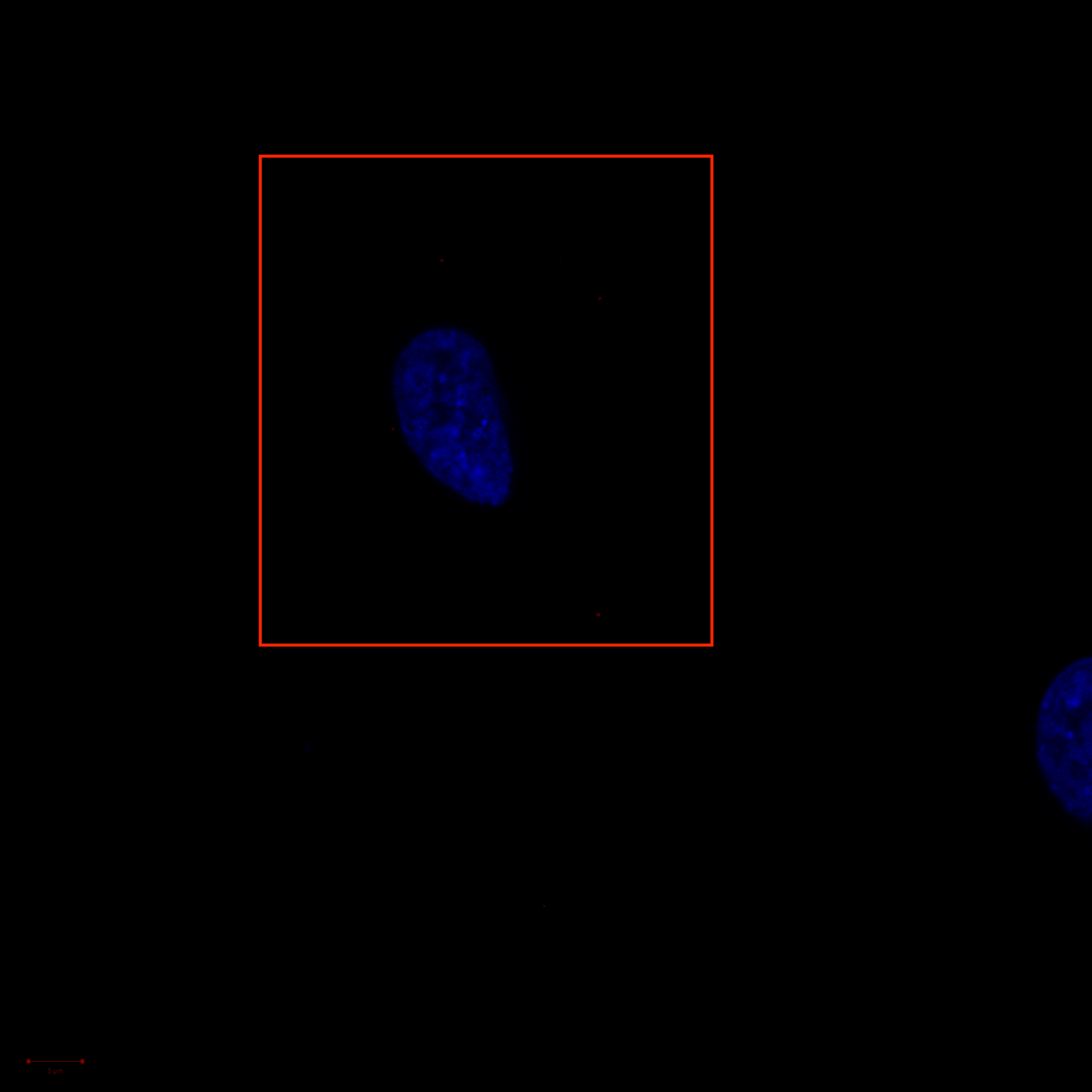

Supplement: Supplementary file 13 — Source data Fig. 3 [file 44319_2025_425_MOESM13_ESM.zip › Figure 3/3C/DAPI.tif]

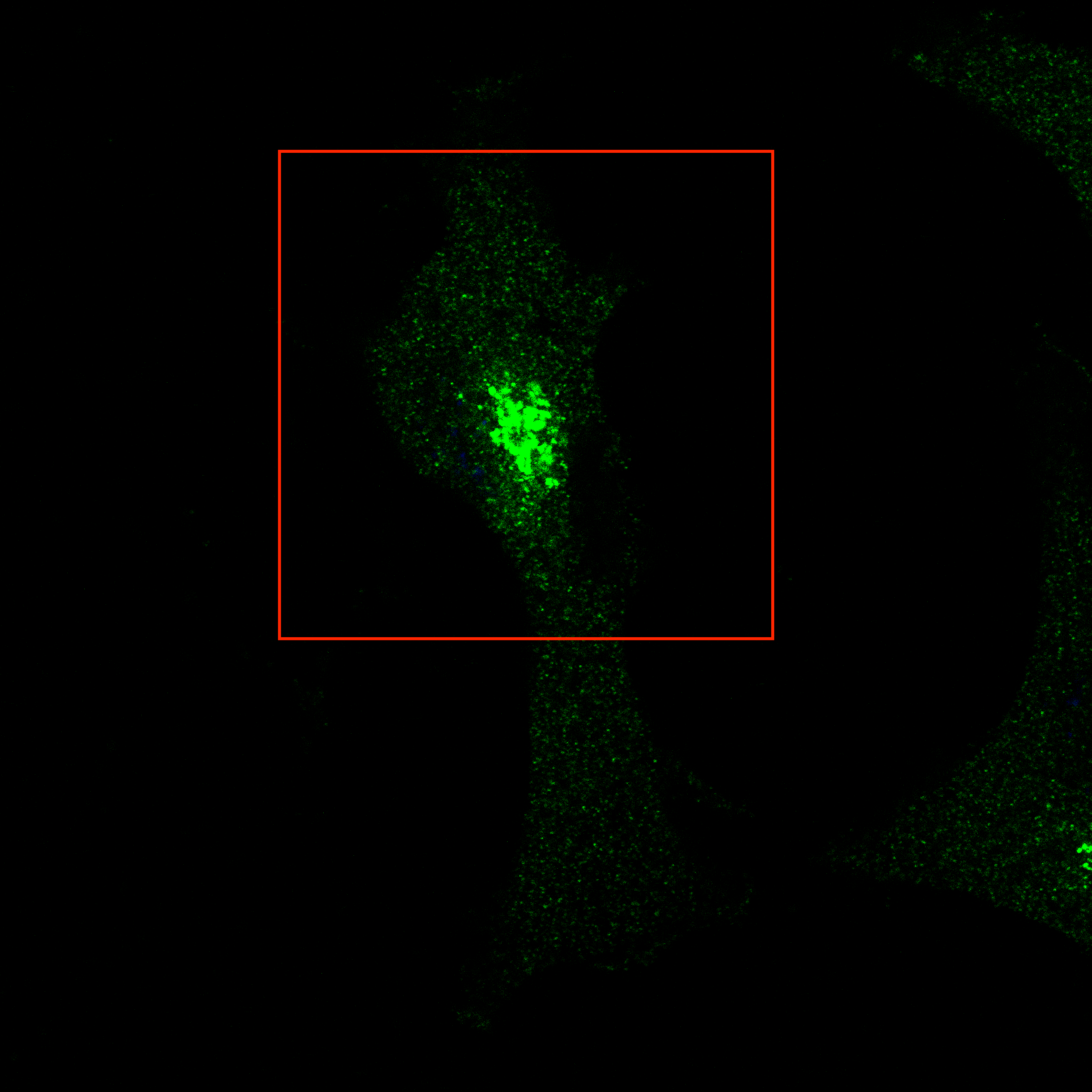

Supplement: Supplementary file 13 — Source data Fig. 3 [file 44319_2025_425_MOESM13_ESM.zip › Figure 3/3C/praja2.tif]

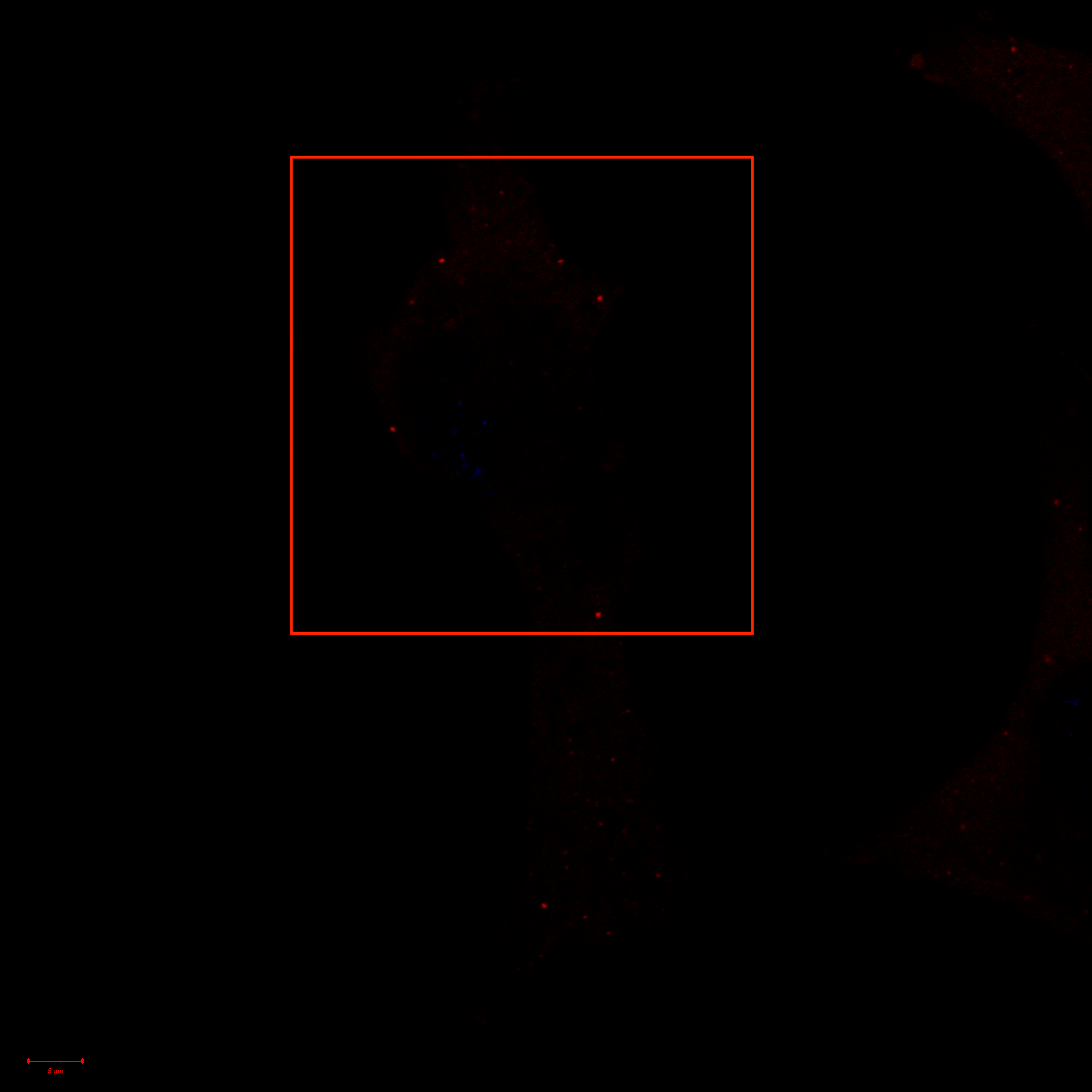

Supplement: Supplementary file 13 — Source data Fig. 3 [file 44319_2025_425_MOESM13_ESM.zip › Figure 3/3C/DDX6.tif]

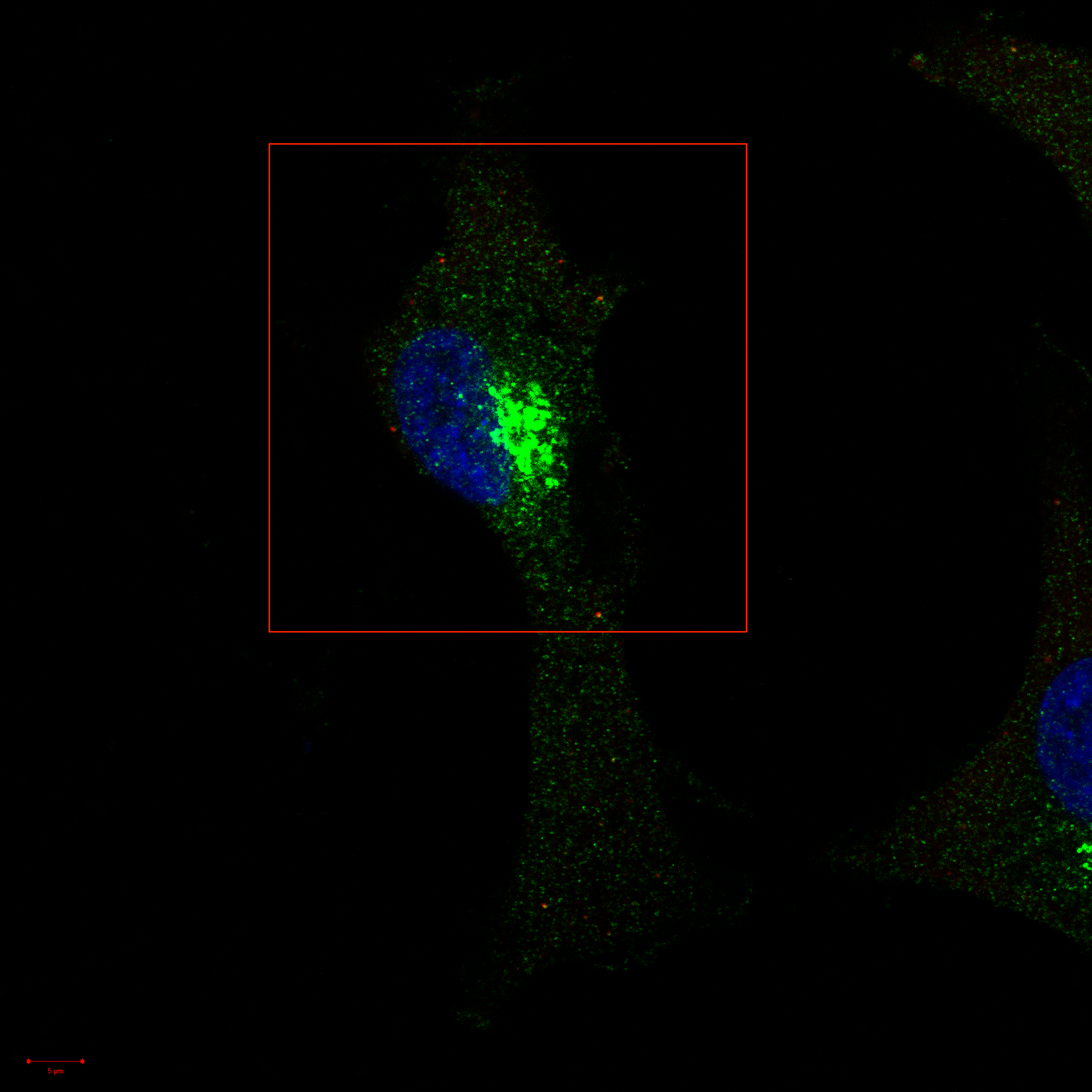

Supplement: Supplementary file 13 — Source data Fig. 3 [file 44319_2025_425_MOESM13_ESM.zip › Figure 3/3C/Merge.tiff]

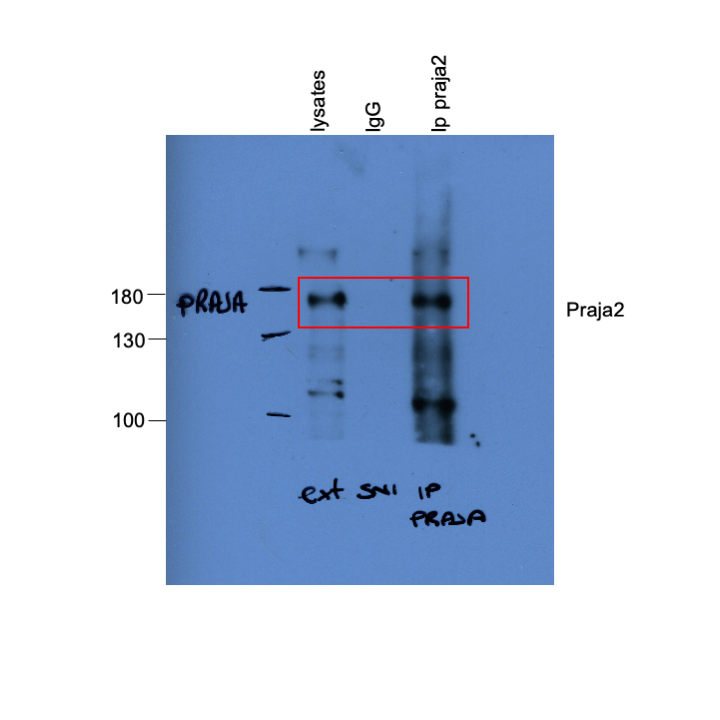

Supplement: Supplementary file 13 — Source data Fig. 3 [file 44319_2025_425_MOESM13_ESM.zip › Figure 3/3D/praja2.tiff]

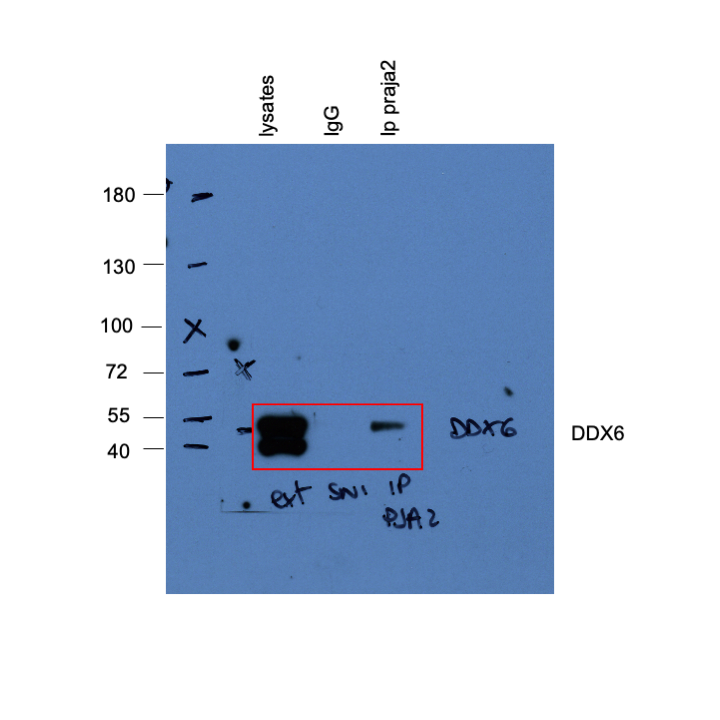

Supplement: Supplementary file 13 — Source data Fig. 3 [file 44319_2025_425_MOESM13_ESM.zip › Figure 3/3D/DDX6.tiff]

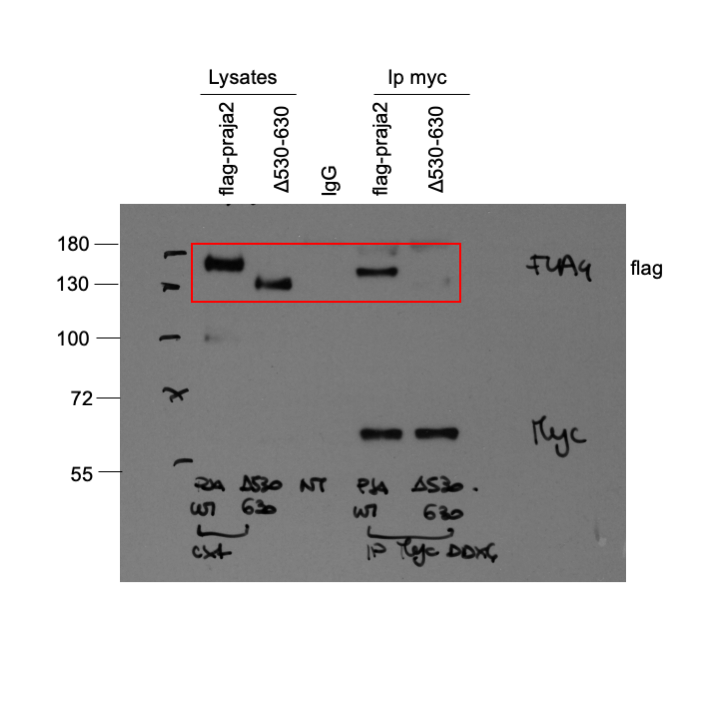

Supplement: Supplementary file 13 — Source data Fig. 3 [file 44319_2025_425_MOESM13_ESM.zip › Figure 3/3H/flag.tiff]

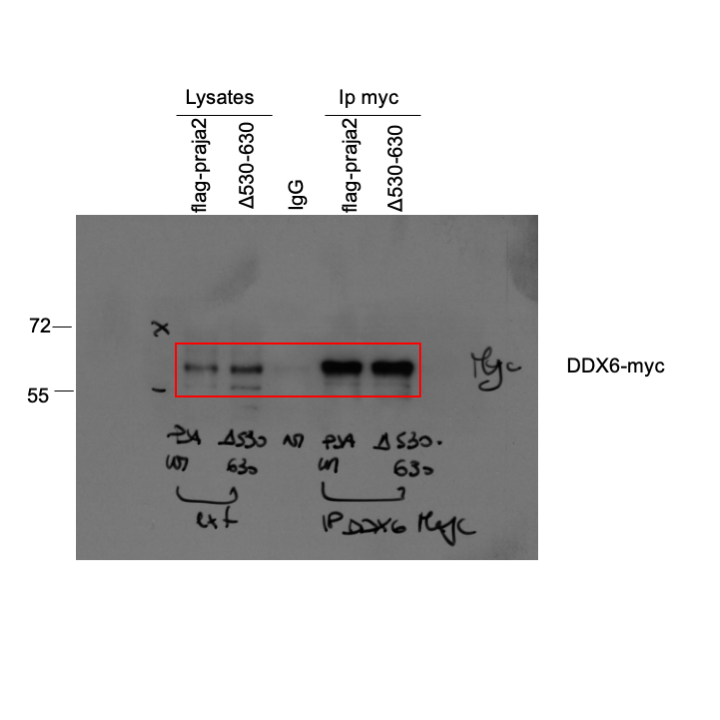

Supplement: Supplementary file 13 — Source data Fig. 3 [file 44319_2025_425_MOESM13_ESM.zip › Figure 3/3H/DDX6-myc.tiff]

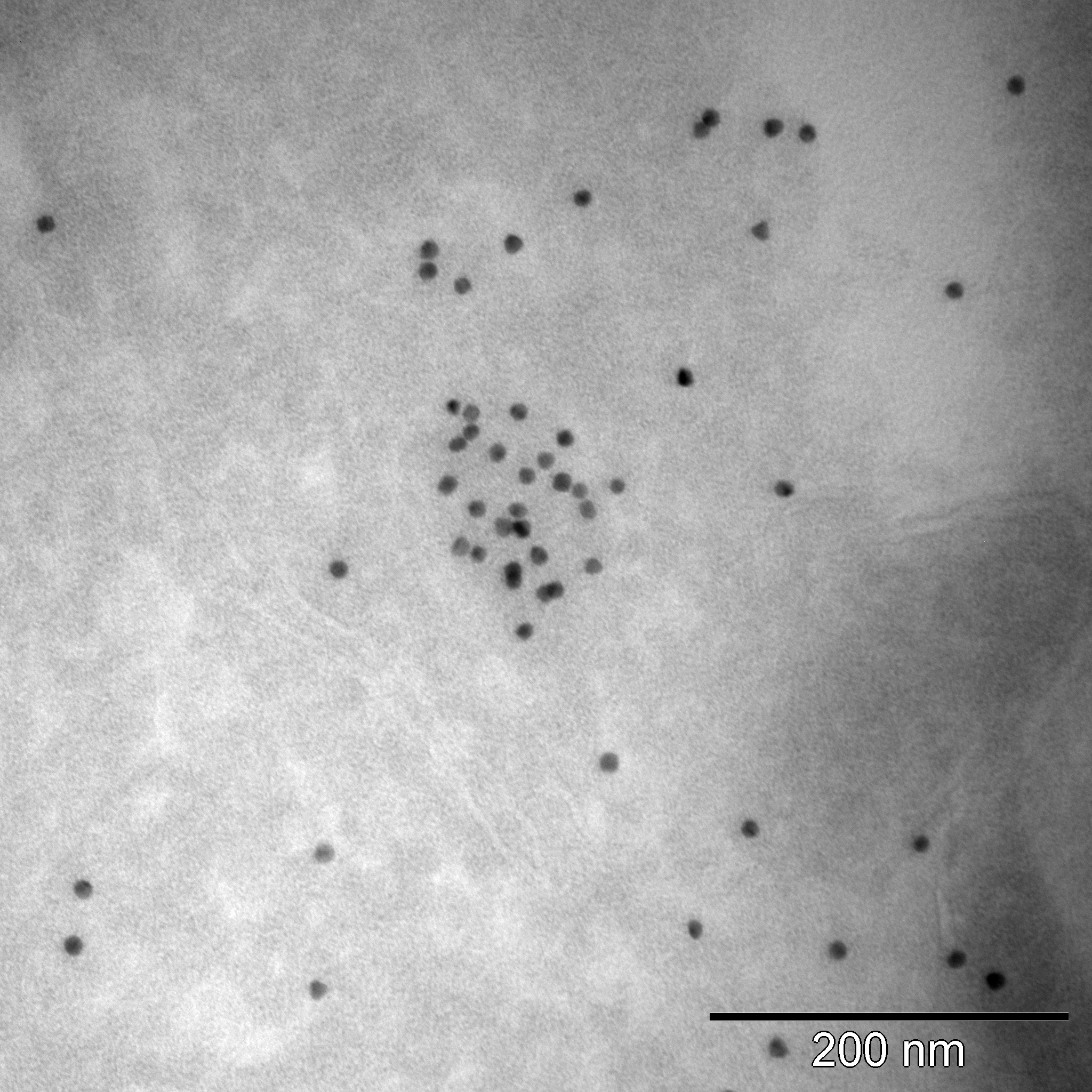

Supplement: Supplementary file 13 — Source data Fig. 3 [file 44319_2025_425_MOESM13_ESM.zip › Figure 3/3A/EM.tif]

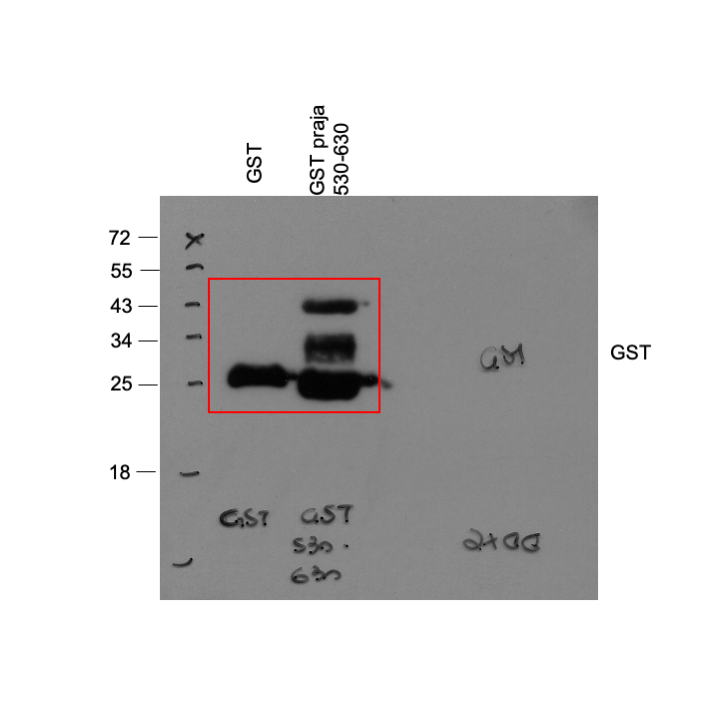

Supplement: Supplementary file 13 — Source data Fig. 3 [file 44319_2025_425_MOESM13_ESM.zip › Figure 3/3I/GST.tiff]

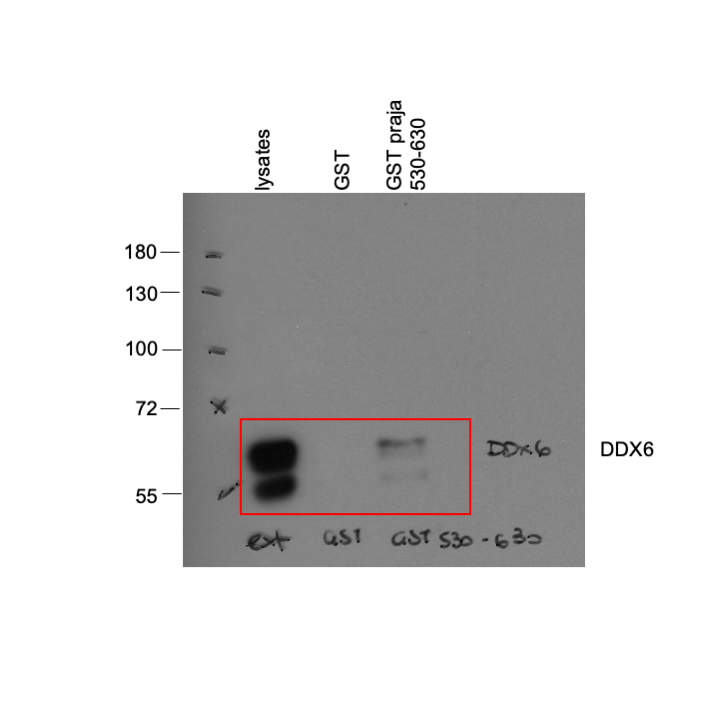

Supplement: Supplementary file 13 — Source data Fig. 3 [file 44319_2025_425_MOESM13_ESM.zip › Figure 3/3I/DDX6.tiff]

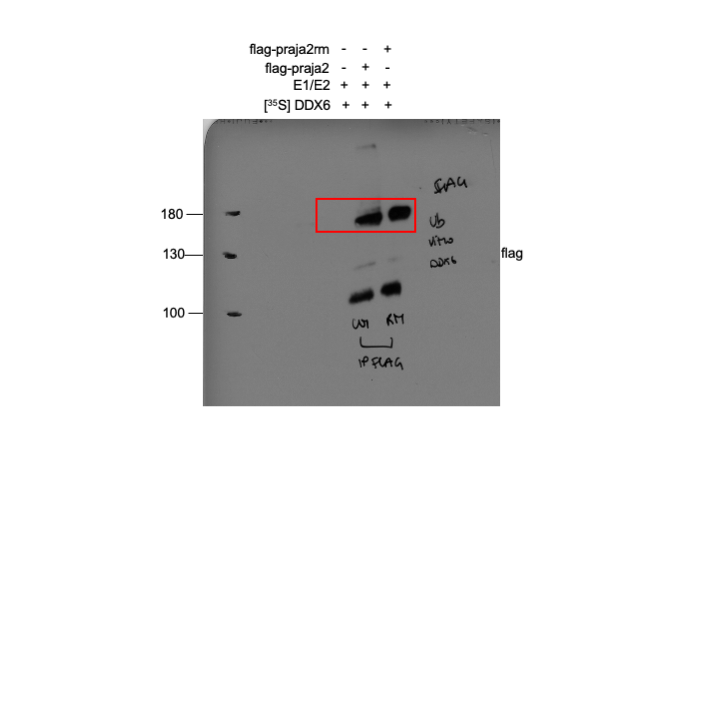

Supplement: Supplementary file 14 — Source data Fig. 4 [file 44319_2025_425_MOESM14_ESM.zip › Figure 4/Fig.4B/flag.tiff]

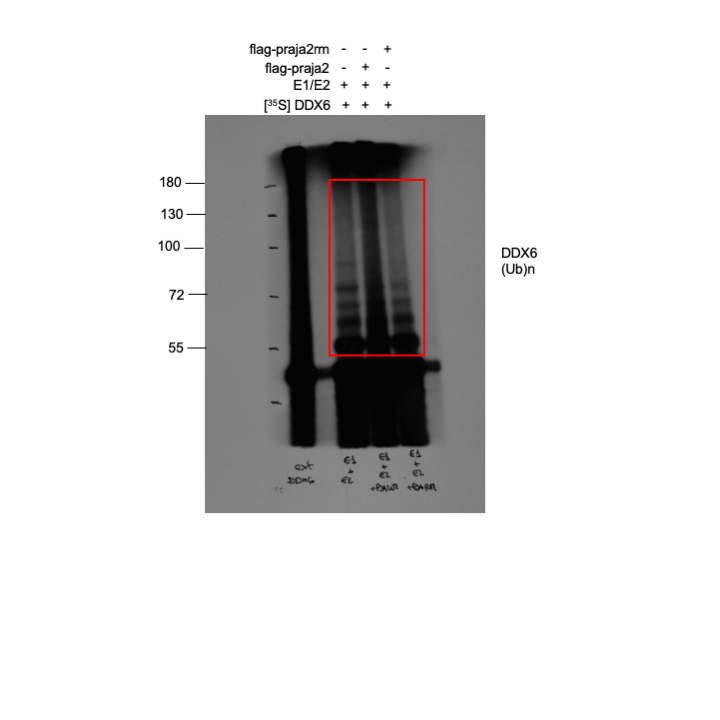

Supplement: Supplementary file 14 — Source data Fig. 4 [file 44319_2025_425_MOESM14_ESM.zip › Figure 4/Fig.4B/DDX(ub)n.tiff]

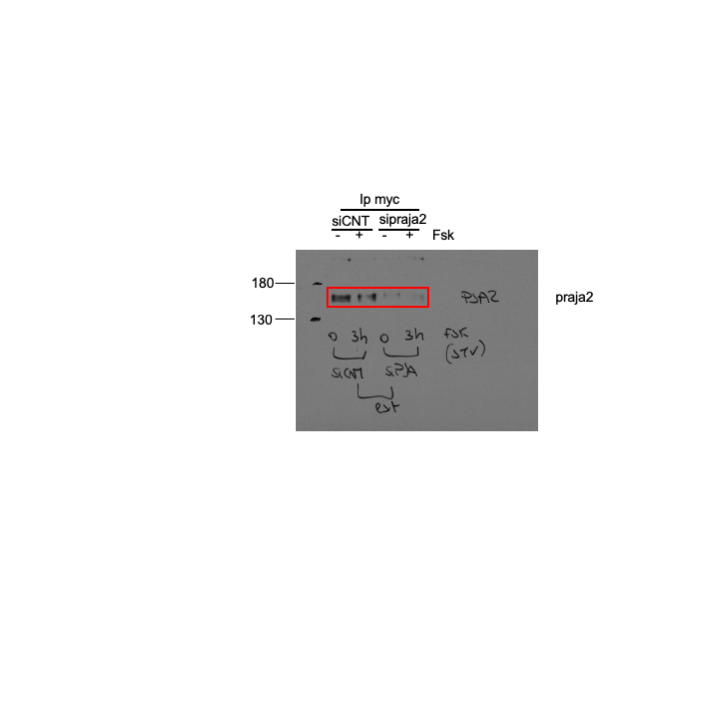

Supplement: Supplementary file 14 — Source data Fig. 4 [file 44319_2025_425_MOESM14_ESM.zip › Figure 4/Fig.4C/praja2.tiff]

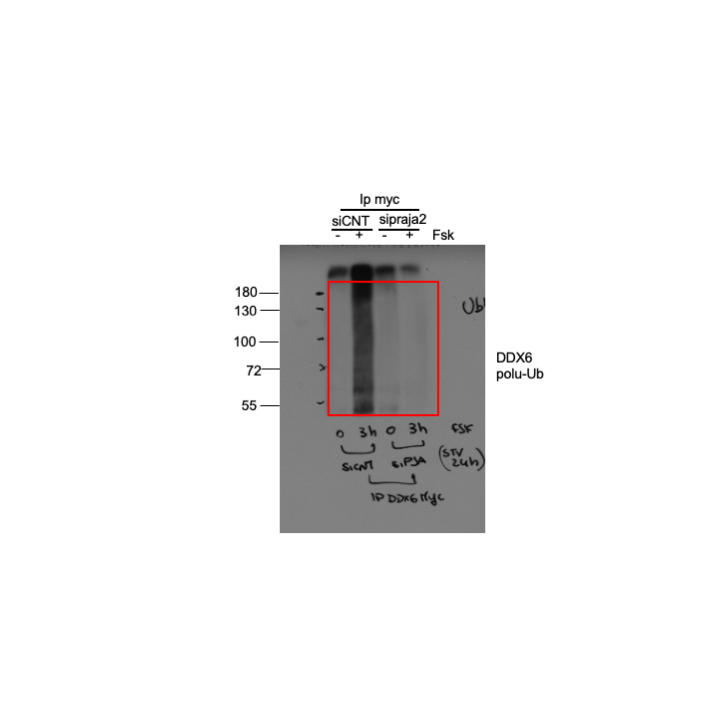

Supplement: Supplementary file 14 — Source data Fig. 4 [file 44319_2025_425_MOESM14_ESM.zip › Figure 4/Fig.4C/DDX6 poly-Ub.tiff]

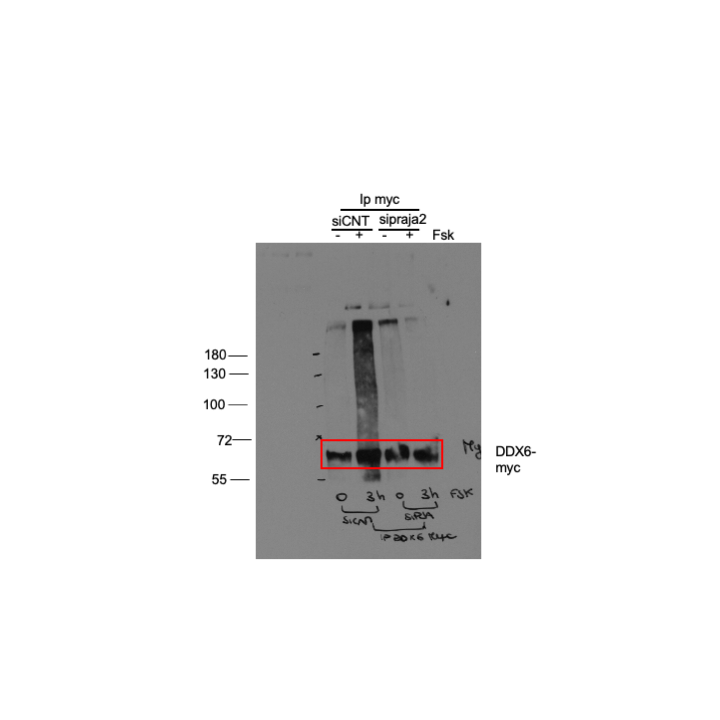

Supplement: Supplementary file 14 — Source data Fig. 4 [file 44319_2025_425_MOESM14_ESM.zip › Figure 4/Fig.4C/DDX6-myc.tiff]

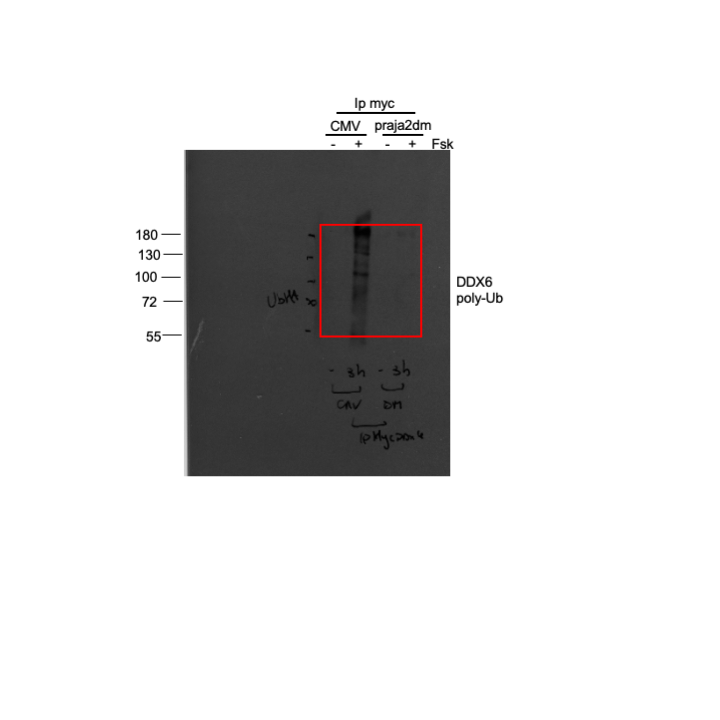

Supplement: Supplementary file 14 — Source data Fig. 4 [file 44319_2025_425_MOESM14_ESM.zip › Figure 4/Fig.4D/DDX6 poly-Ub.tiff]

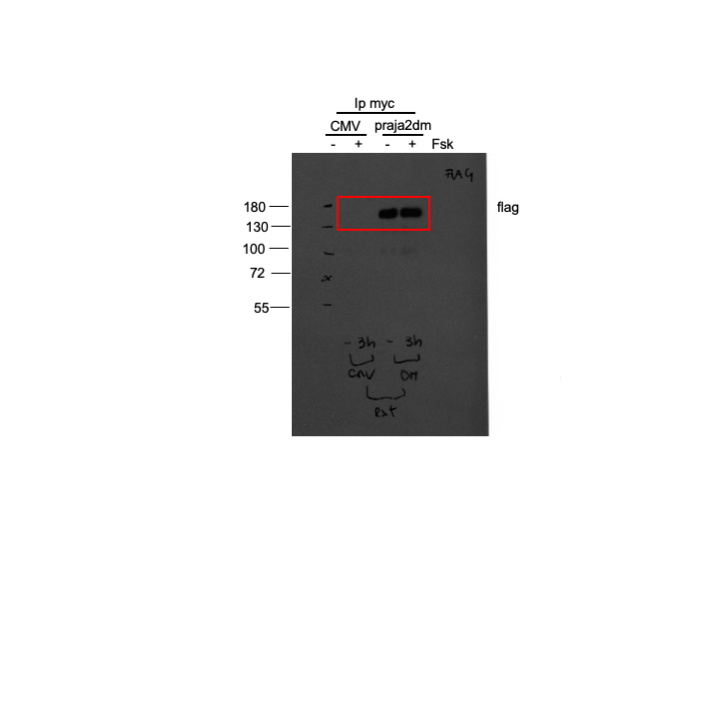

Supplement: Supplementary file 14 — Source data Fig. 4 [file 44319_2025_425_MOESM14_ESM.zip › Figure 4/Fig.4D/flag.tiff]

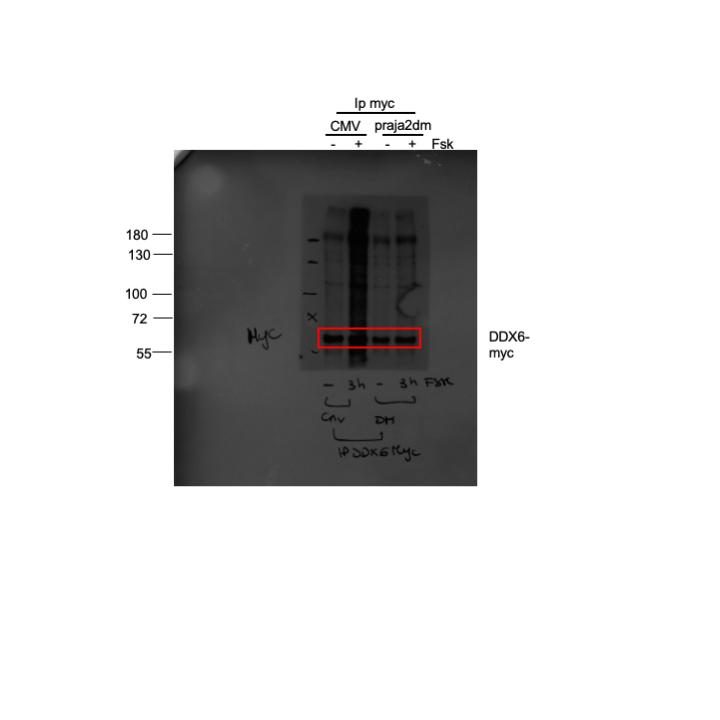

Supplement: Supplementary file 14 — Source data Fig. 4 [file 44319_2025_425_MOESM14_ESM.zip › Figure 4/Fig.4D/DDX6-myc.tiff]

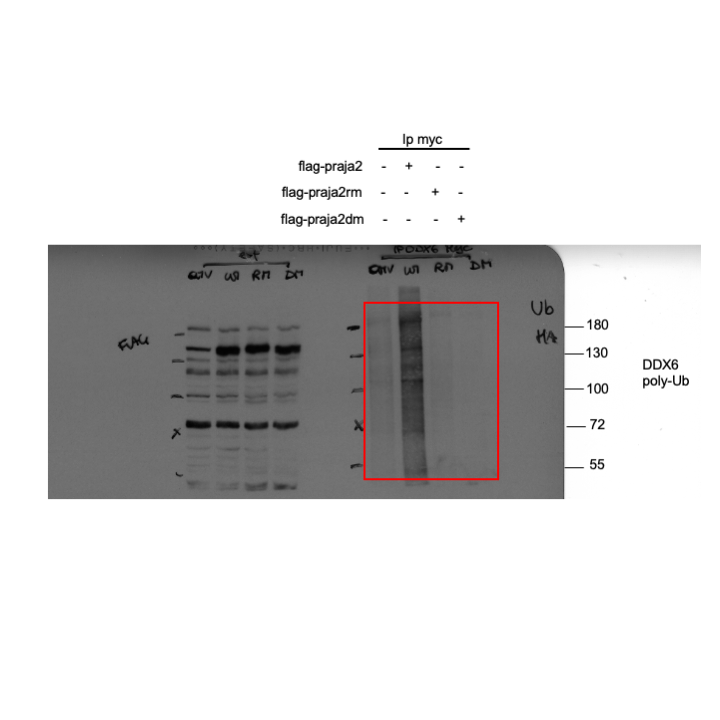

Supplement: Supplementary file 14 — Source data Fig. 4 [file 44319_2025_425_MOESM14_ESM.zip › Figure 4/Fig.4A/DDX6 poly-Ub.tiff]

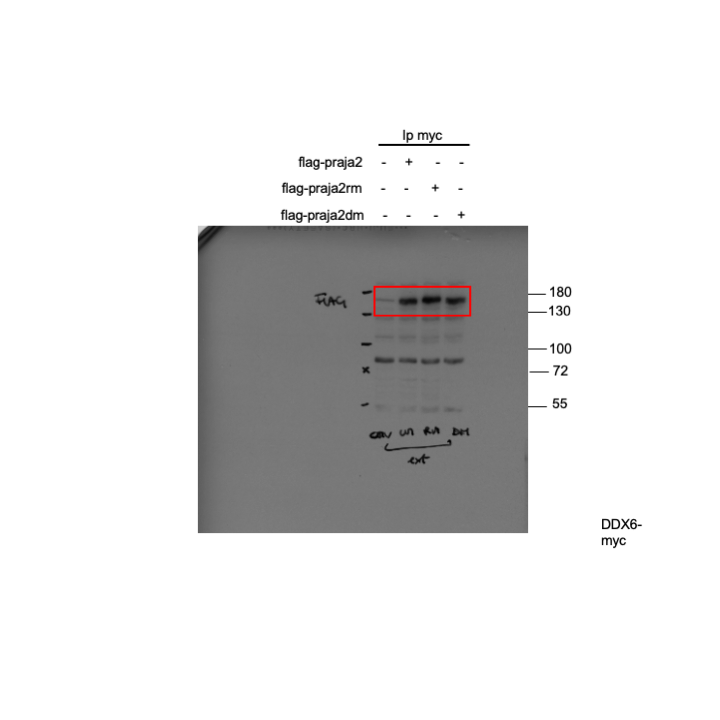

Supplement: Supplementary file 14 — Source data Fig. 4 [file 44319_2025_425_MOESM14_ESM.zip › Figure 4/Fig.4A/flag.tiff]

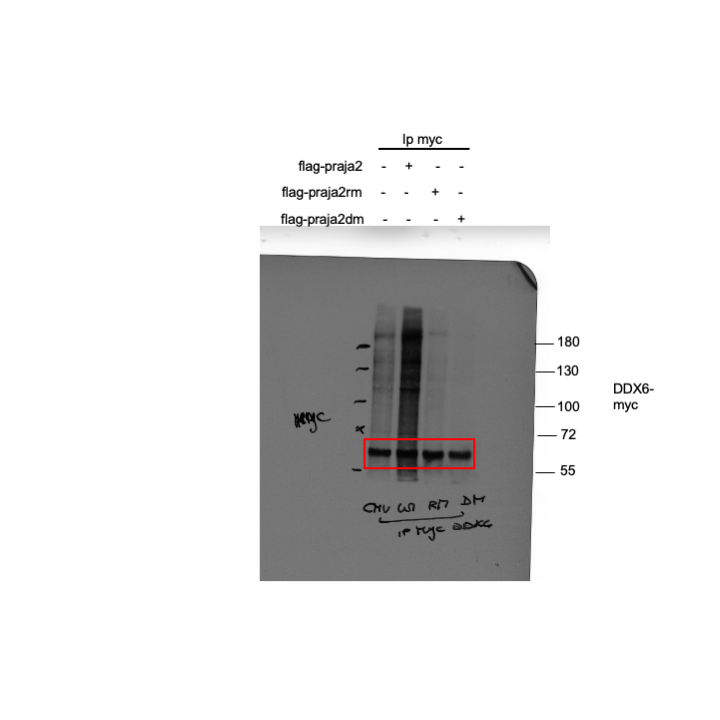

Supplement: Supplementary file 14 — Source data Fig. 4 [file 44319_2025_425_MOESM14_ESM.zip › Figure 4/Fig.4A/DDX6-myc.tiff]

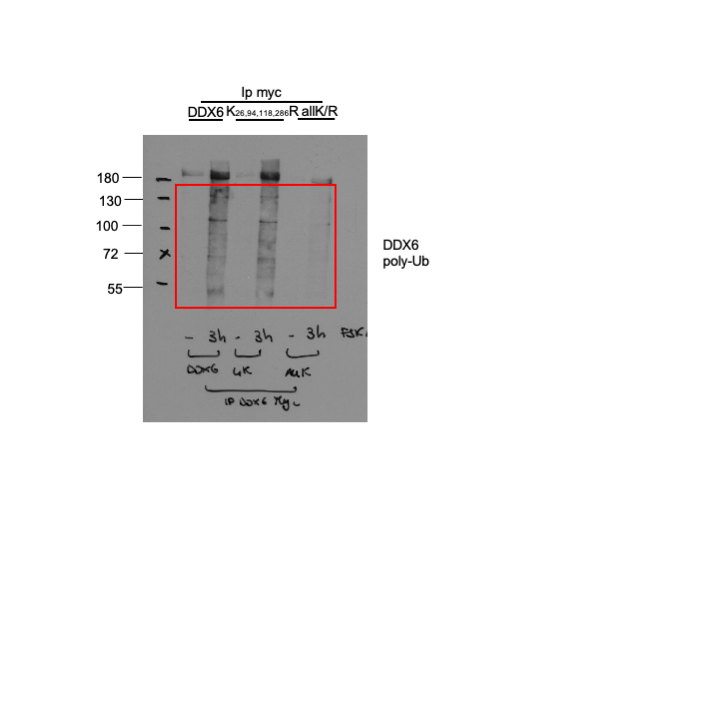

Supplement: Supplementary file 14 — Source data Fig. 4 [file 44319_2025_425_MOESM14_ESM.zip › Figure 4/Fig.4F/DDX6 poly-Ub.tiff]

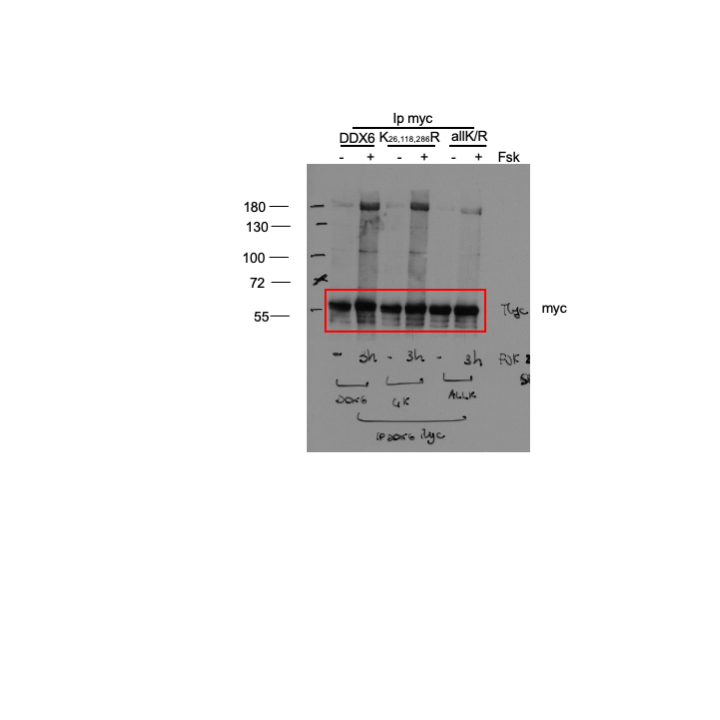

Supplement: Supplementary file 14 — Source data Fig. 4 [file 44319_2025_425_MOESM14_ESM.zip › Figure 4/Fig.4F/DDX6-myc.tiff]

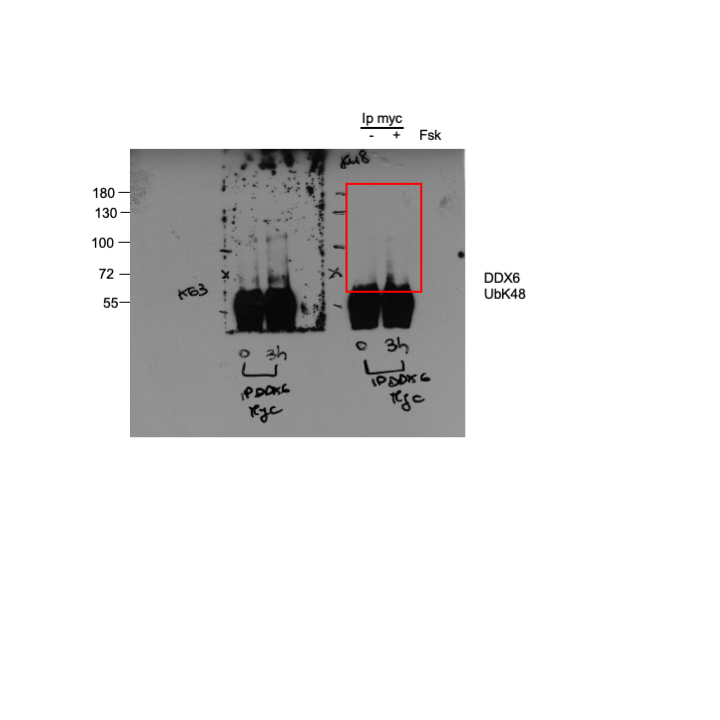

Supplement: Supplementary file 14 — Source data Fig. 4 [file 44319_2025_425_MOESM14_ESM.zip › Figure 4/Fig.4H/DDX6 UbK48.tiff]

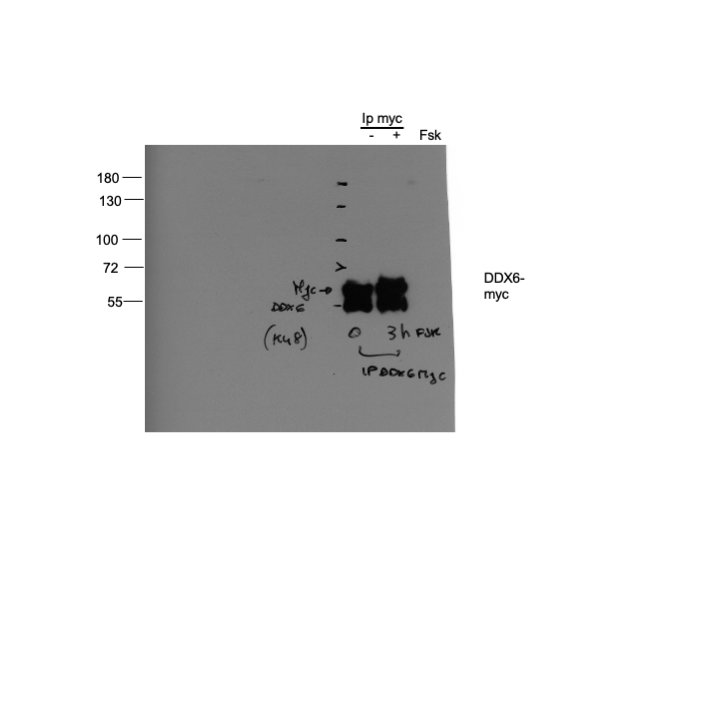

Supplement: Supplementary file 14 — Source data Fig. 4 [file 44319_2025_425_MOESM14_ESM.zip › Figure 4/Fig.4H/DDX6-myc.tiff]

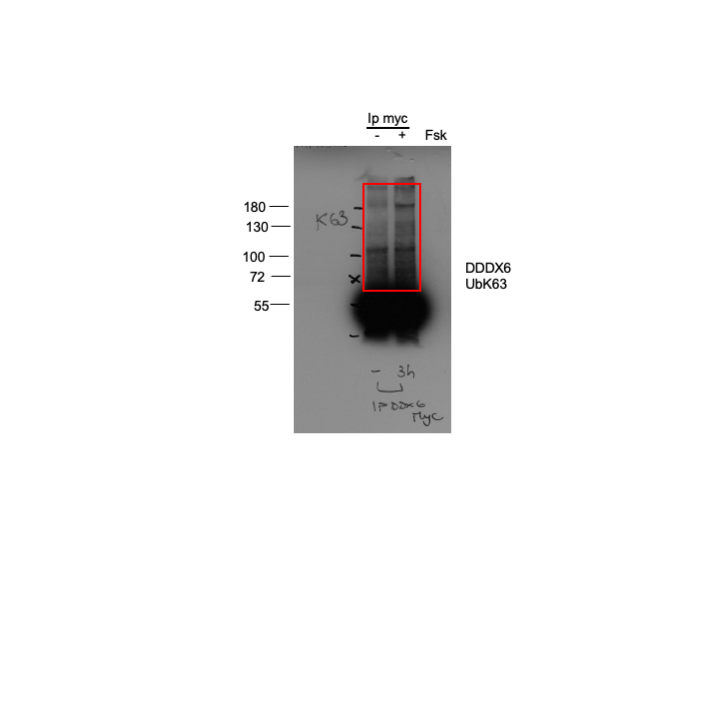

Supplement: Supplementary file 14 — Source data Fig. 4 [file 44319_2025_425_MOESM14_ESM.zip › Figure 4/Fig.4G/DDX6 UbK63.tiff]

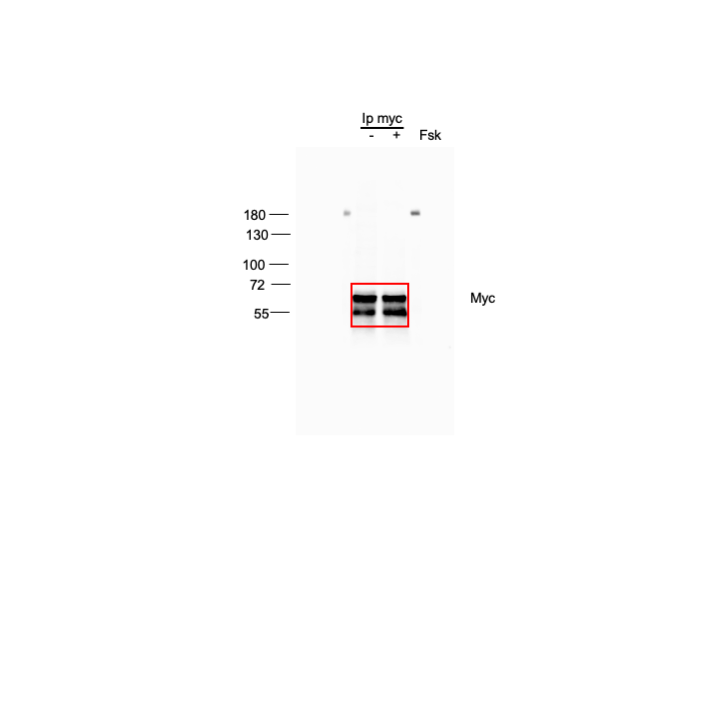

Supplement: Supplementary file 14 — Source data Fig. 4 [file 44319_2025_425_MOESM14_ESM.zip › Figure 4/Fig.4G/DDX6-myc.tiff]

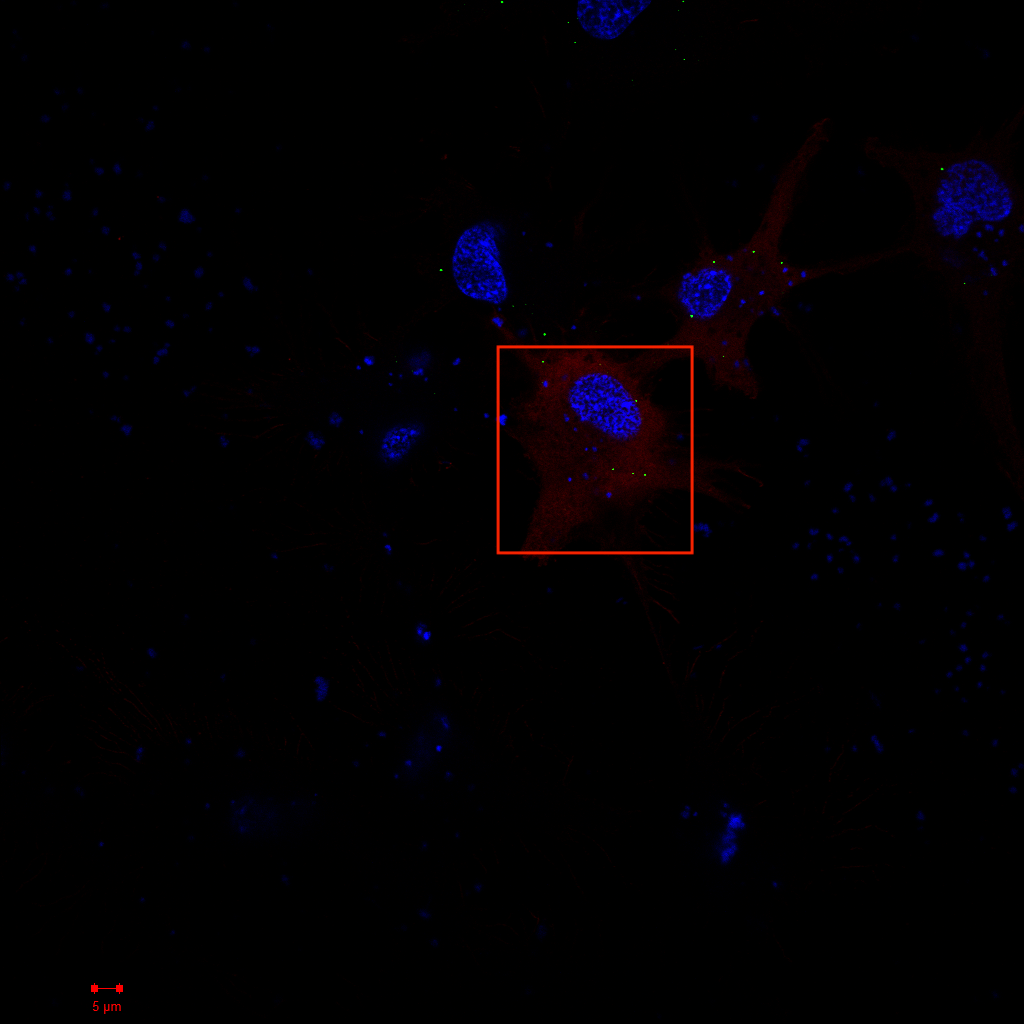

Supplement: Supplementary file 15 — Source data Fig. 5 [file 44319_2025_425_MOESM15_ESM.zip › Figure 5/5I/flag-praja2dm 60' Fsk.tiff]

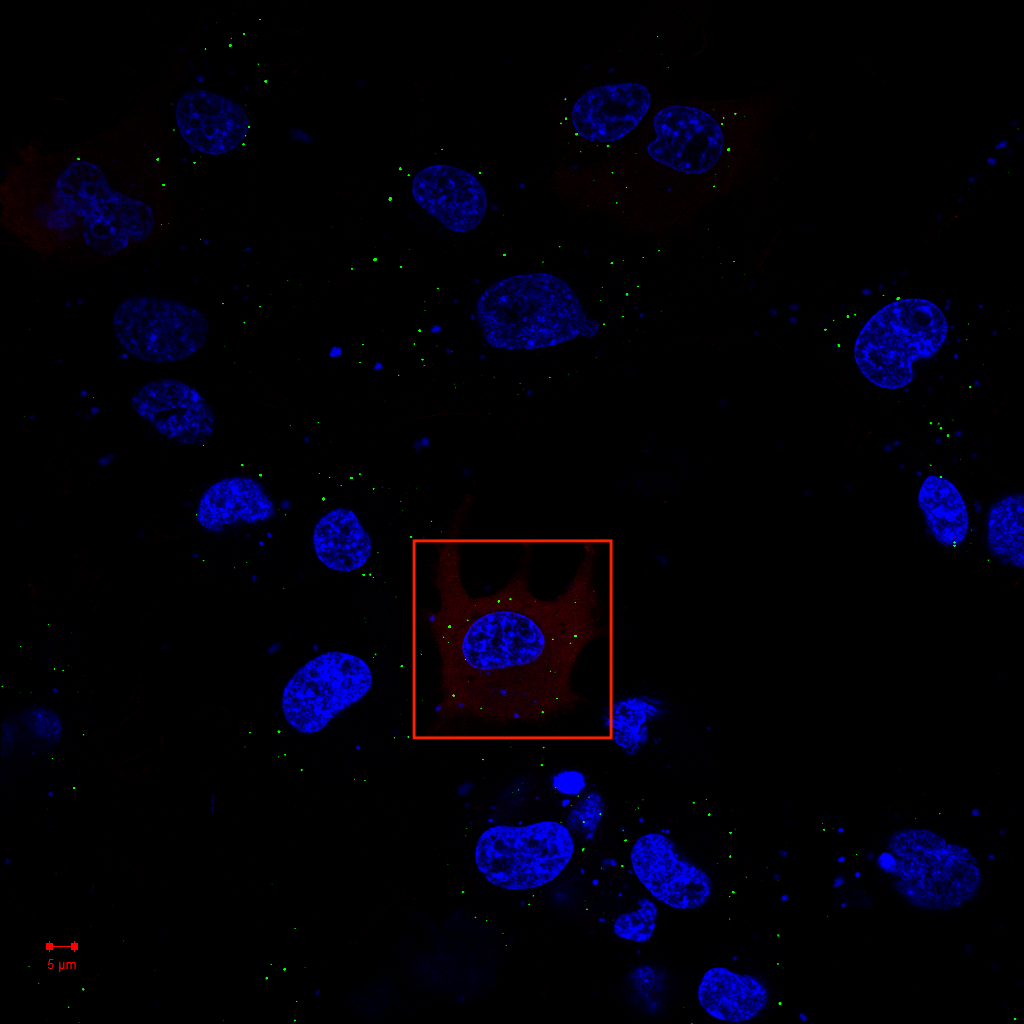

Supplement: Supplementary file 15 — Source data Fig. 5 [file 44319_2025_425_MOESM15_ESM.zip › Figure 5/5I/flag-praja2 60' Fsk.tiff]

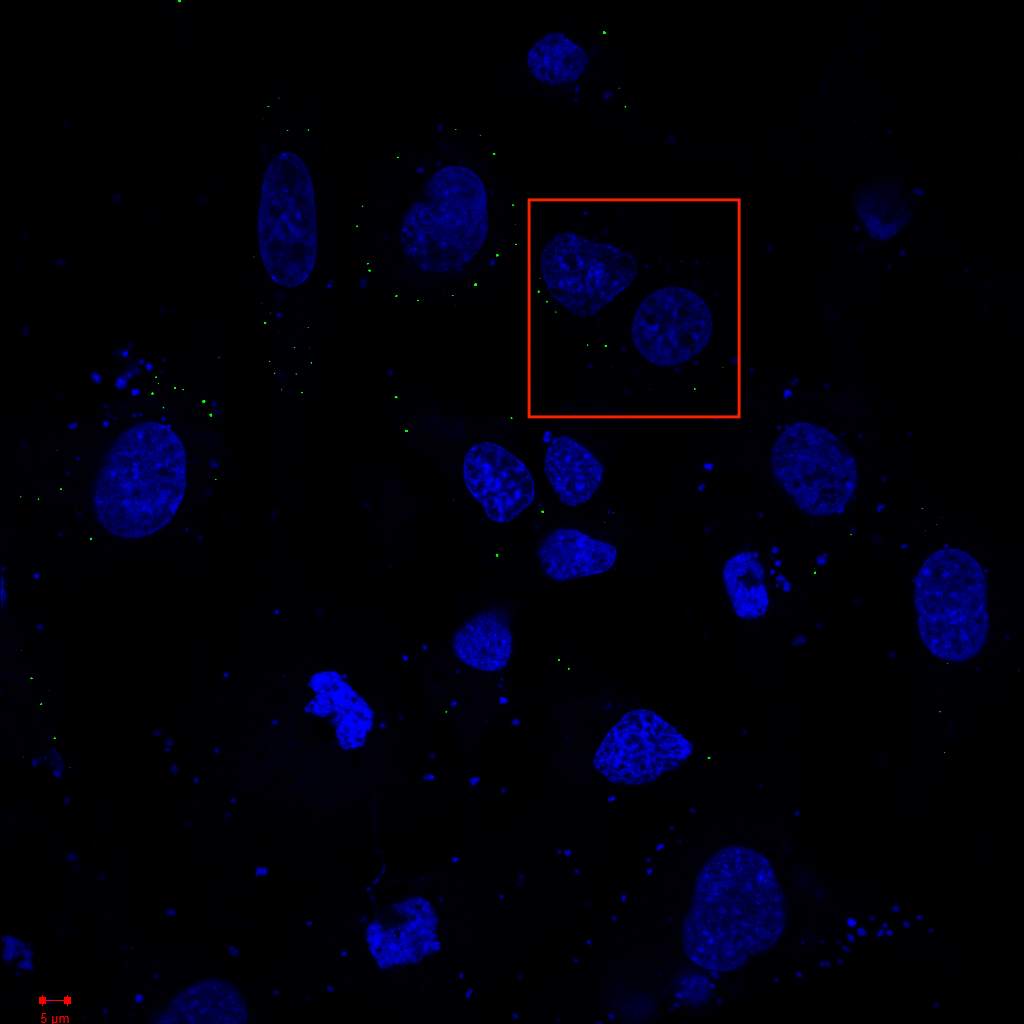

Supplement: Supplementary file 15 — Source data Fig. 5 [file 44319_2025_425_MOESM15_ESM.zip › Figure 5/5I/NT -.tiff]

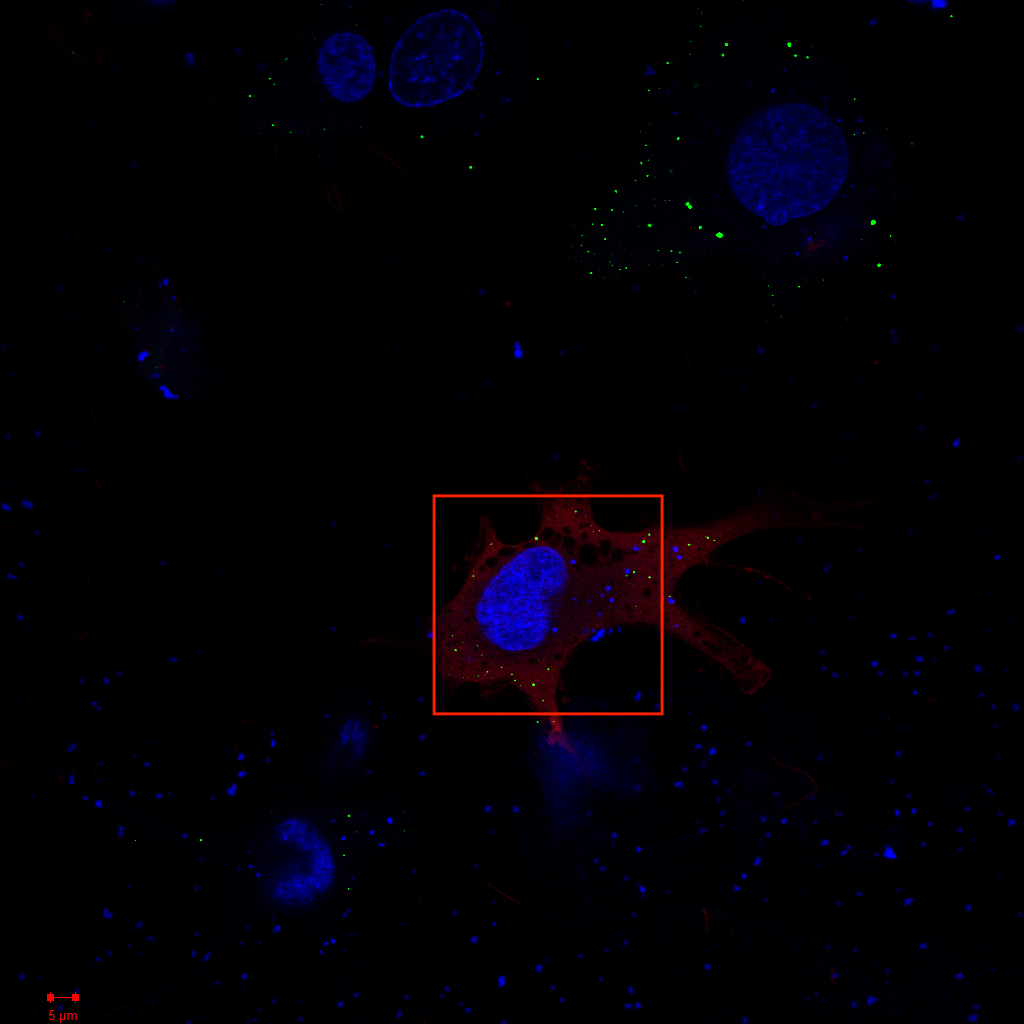

Supplement: Supplementary file 15 — Source data Fig. 5 [file 44319_2025_425_MOESM15_ESM.zip › Figure 5/5I/flag-praja2 -.tiff]

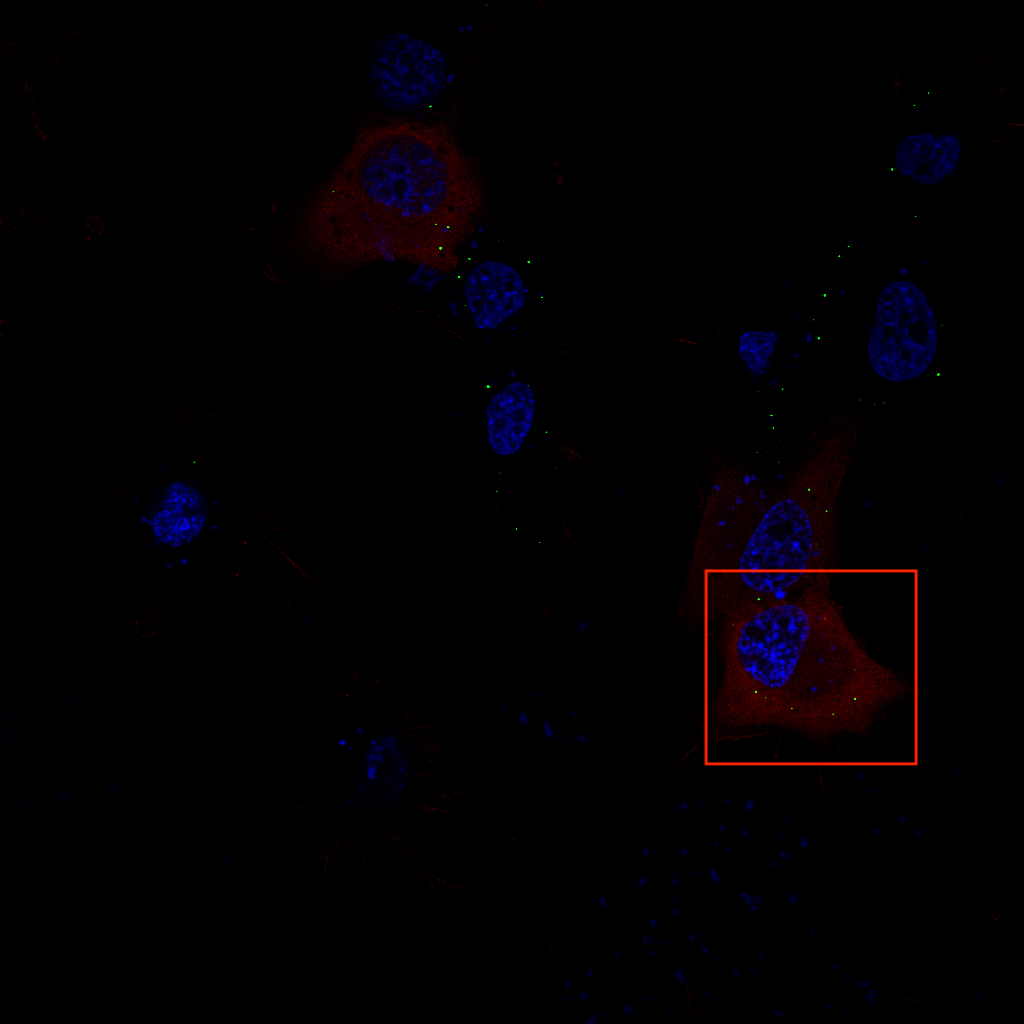

Supplement: Supplementary file 15 — Source data Fig. 5 [file 44319_2025_425_MOESM15_ESM.zip › Figure 5/5I/flag-praja2dm -.tiff]

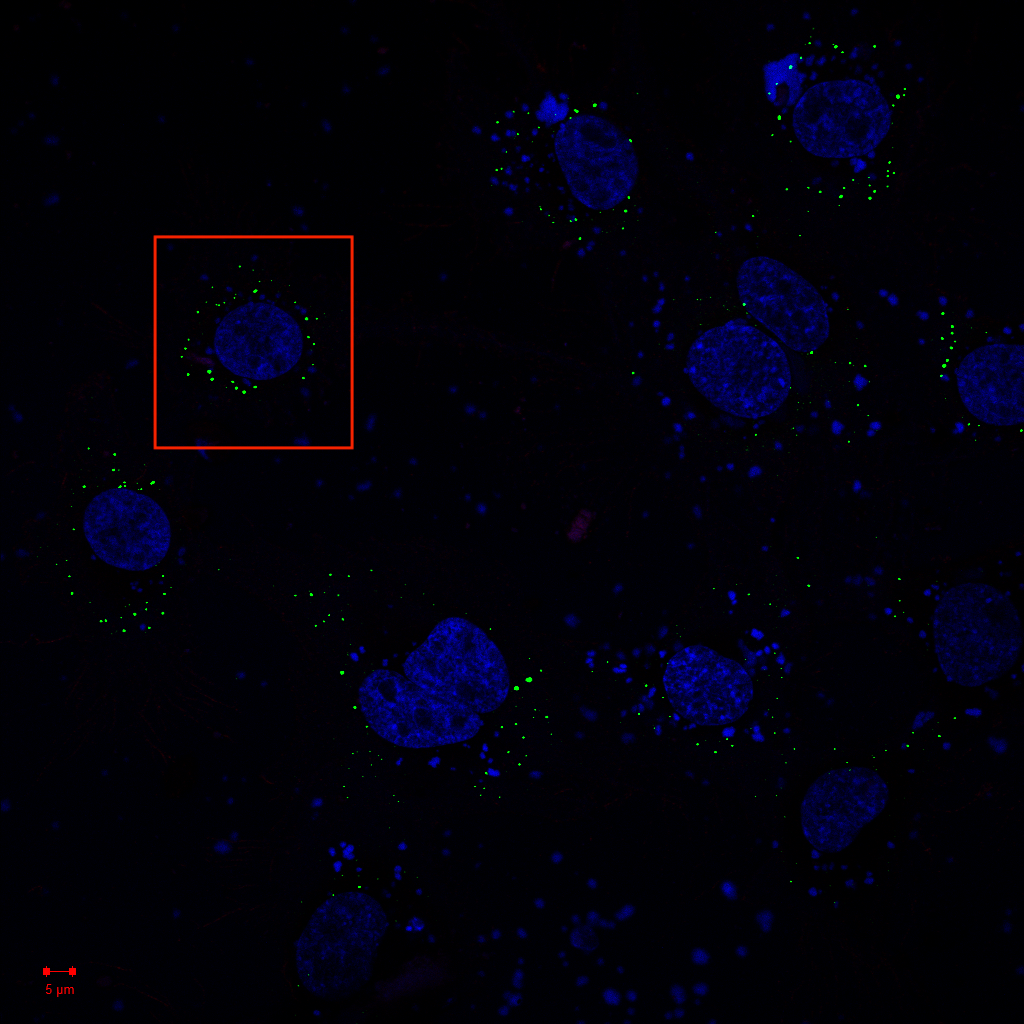

Supplement: Supplementary file 15 — Source data Fig. 5 [file 44319_2025_425_MOESM15_ESM.zip › Figure 5/5I/NT 60' Fsk.tiff]

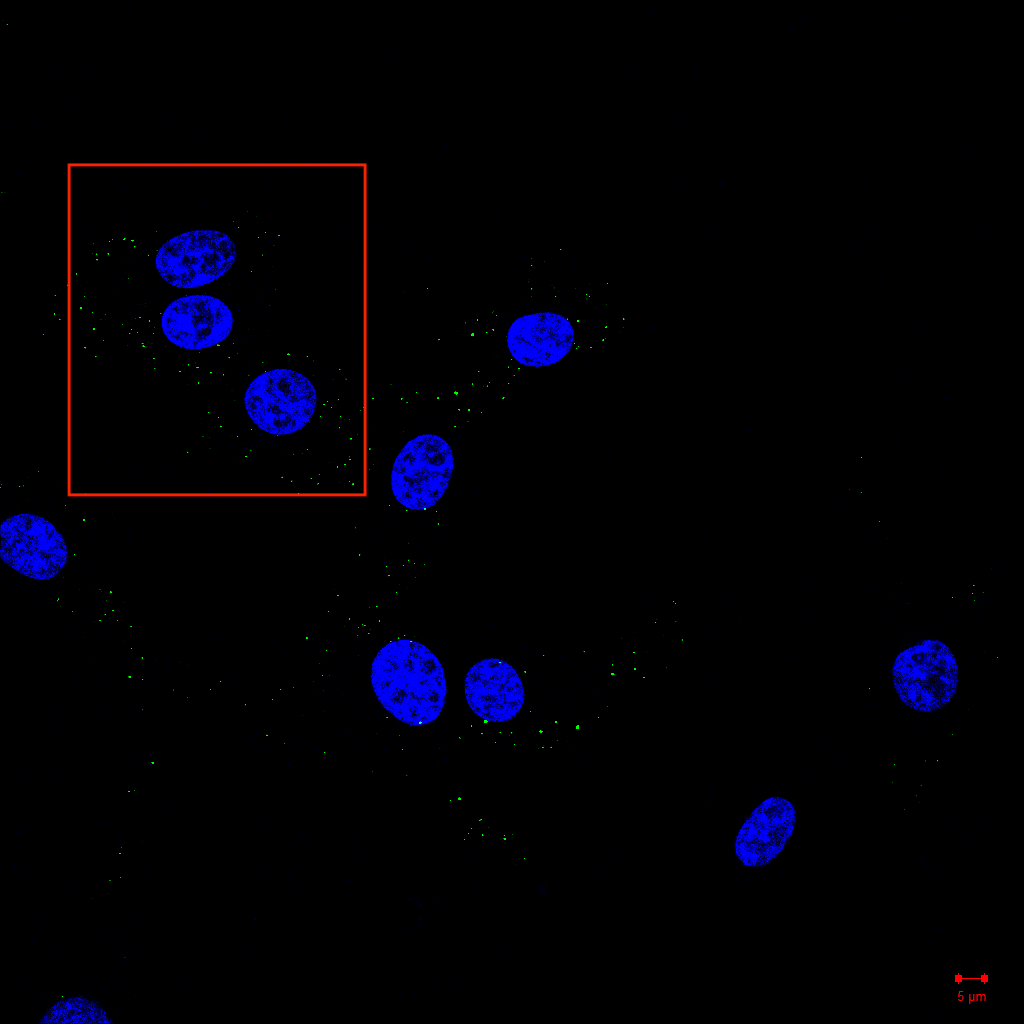

Supplement: Supplementary file 15 — Source data Fig. 5 [file 44319_2025_425_MOESM15_ESM.zip › Figure 5/5G/U87 60' PGE.tiff]

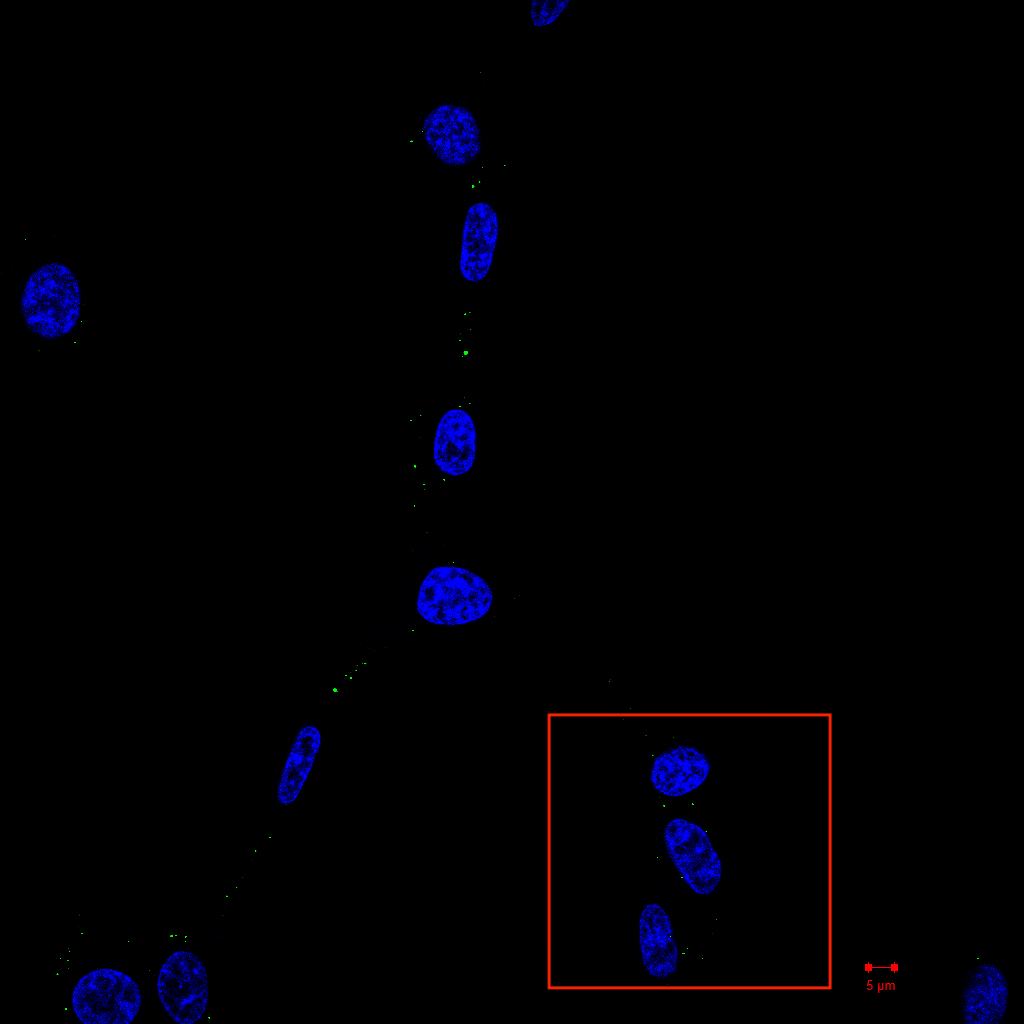

Supplement: Supplementary file 15 — Source data Fig. 5 [file 44319_2025_425_MOESM15_ESM.zip › Figure 5/5G/U87 +KT5720.tiff]

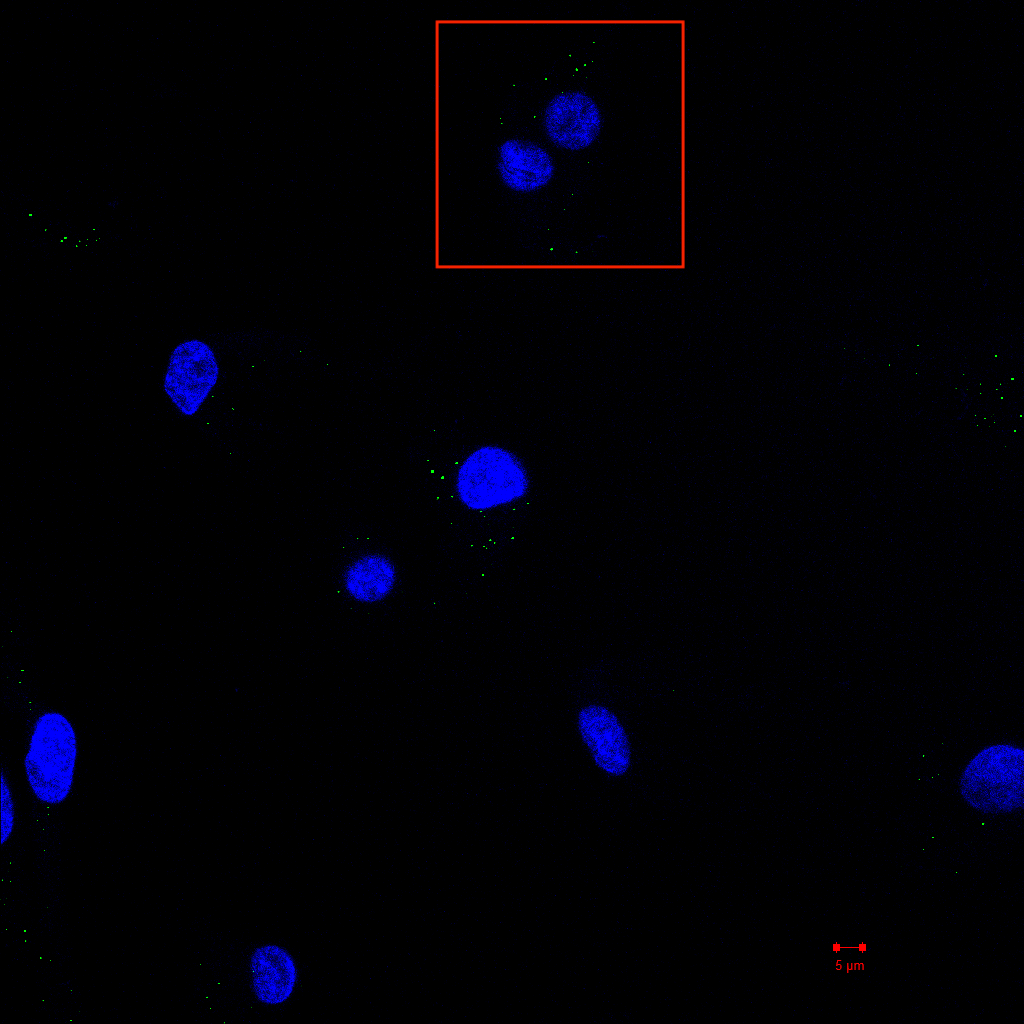

Supplement: Supplementary file 15 — Source data Fig. 5 [file 44319_2025_425_MOESM15_ESM.zip › Figure 5/5G/U87 60' PGE2+KT5720.tiff]

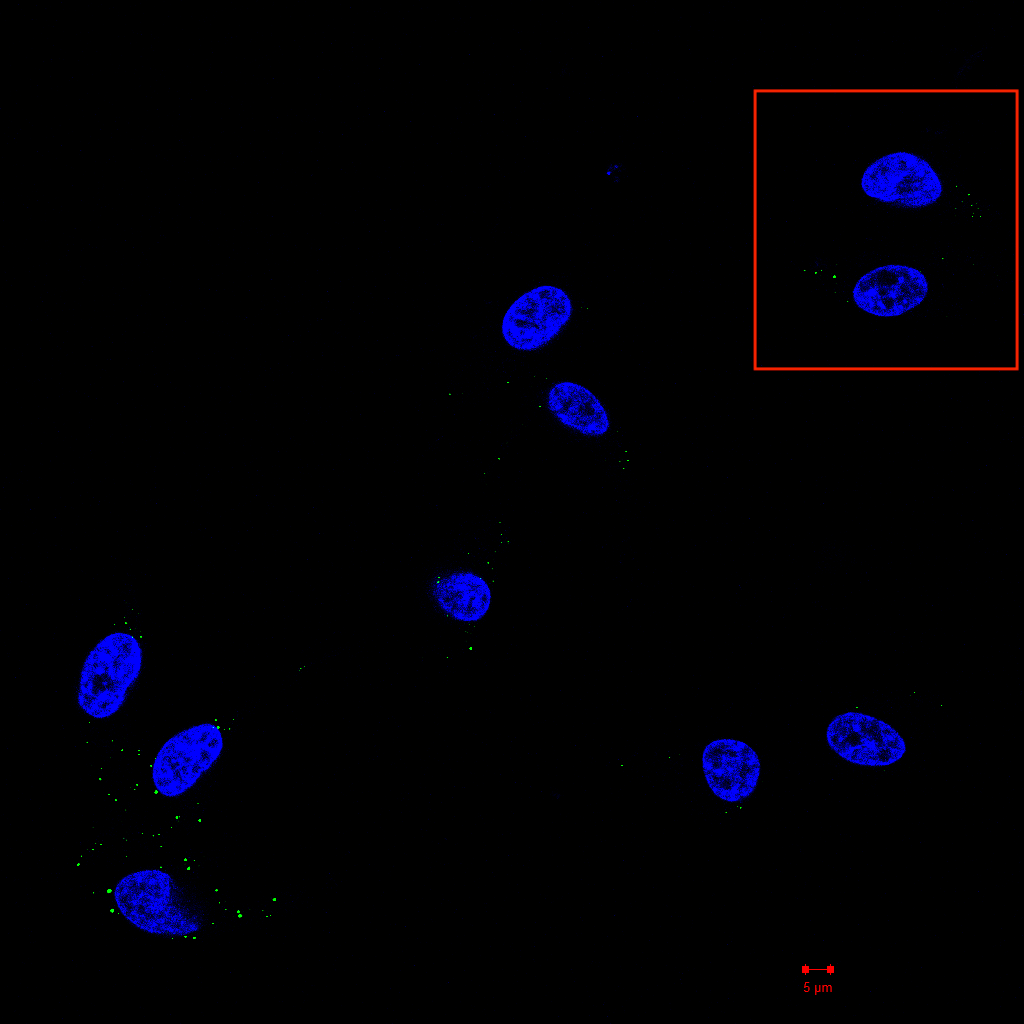

Supplement: Supplementary file 15 — Source data Fig. 5 [file 44319_2025_425_MOESM15_ESM.zip › Figure 5/5G/U87 -.tiff]

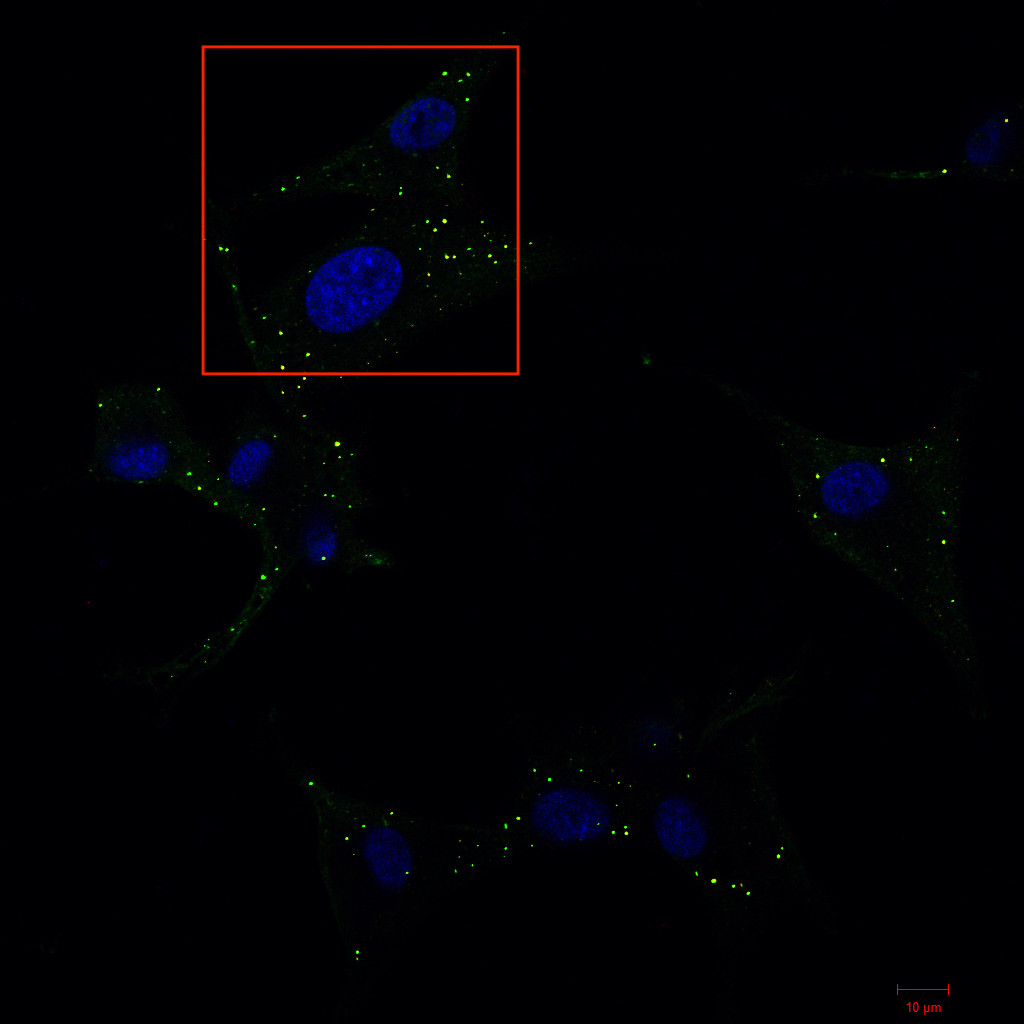

Supplement: Supplementary file 15 — Source data Fig. 5 [file 44319_2025_425_MOESM15_ESM.zip › Figure 5/5A/U87 30' Fsk.tiff]

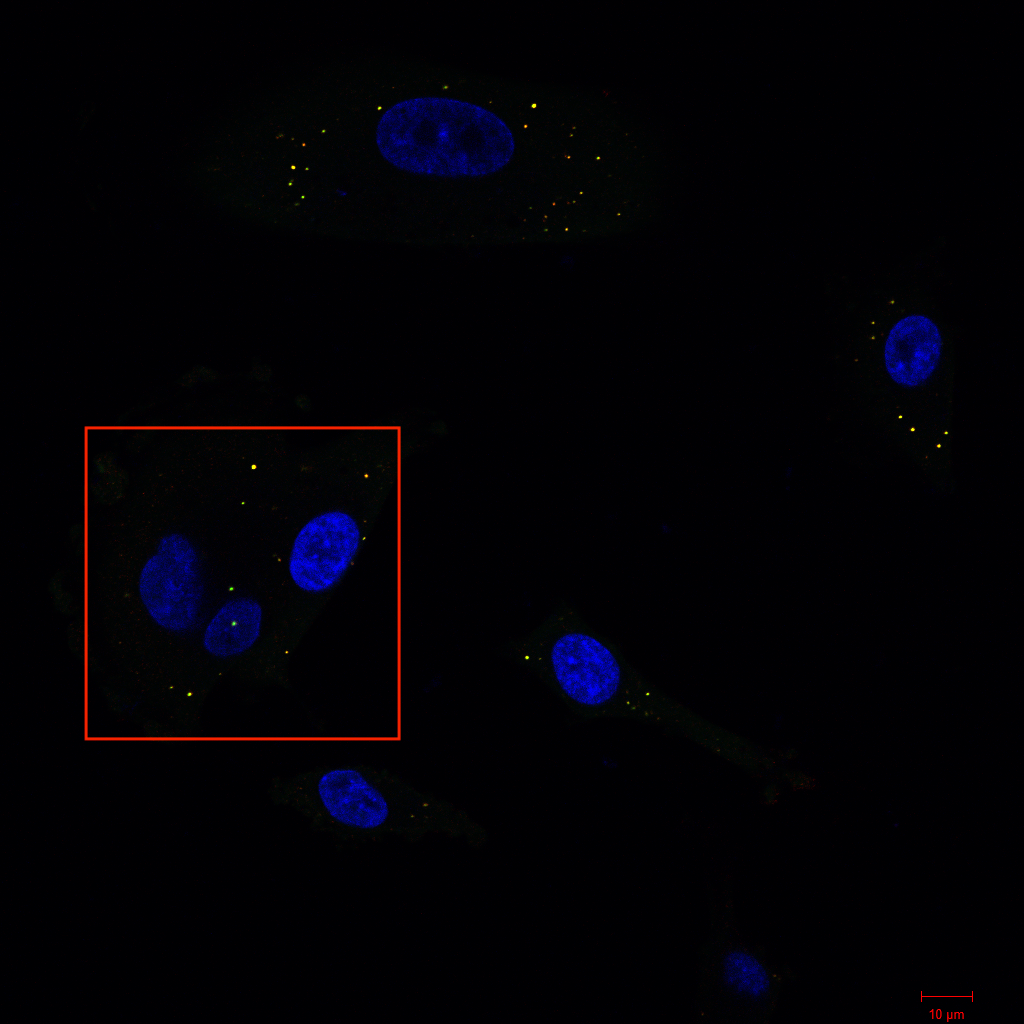

Supplement: Supplementary file 15 — Source data Fig. 5 [file 44319_2025_425_MOESM15_ESM.zip › Figure 5/5A/U87 -.tiff]

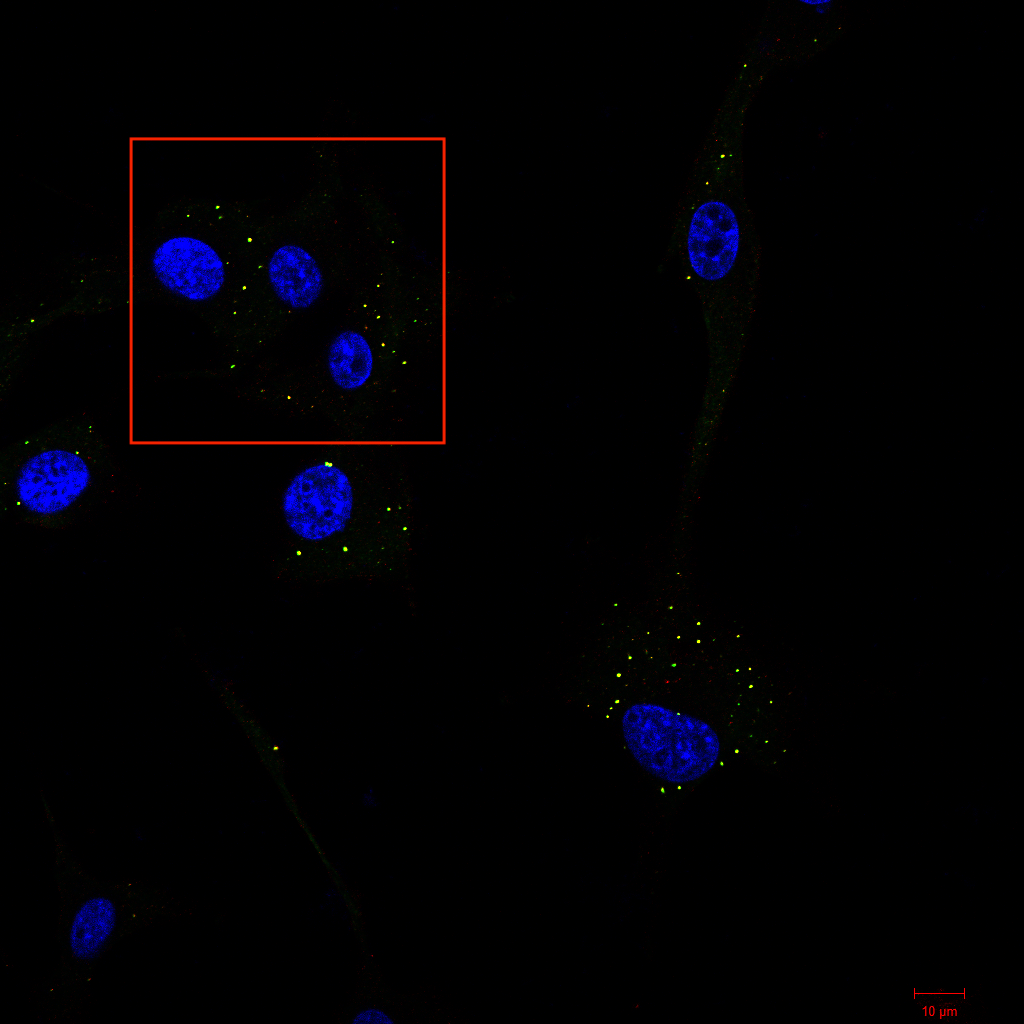

Supplement: Supplementary file 15 — Source data Fig. 5 [file 44319_2025_425_MOESM15_ESM.zip › Figure 5/5A/U87 60'Fsk.tiff]

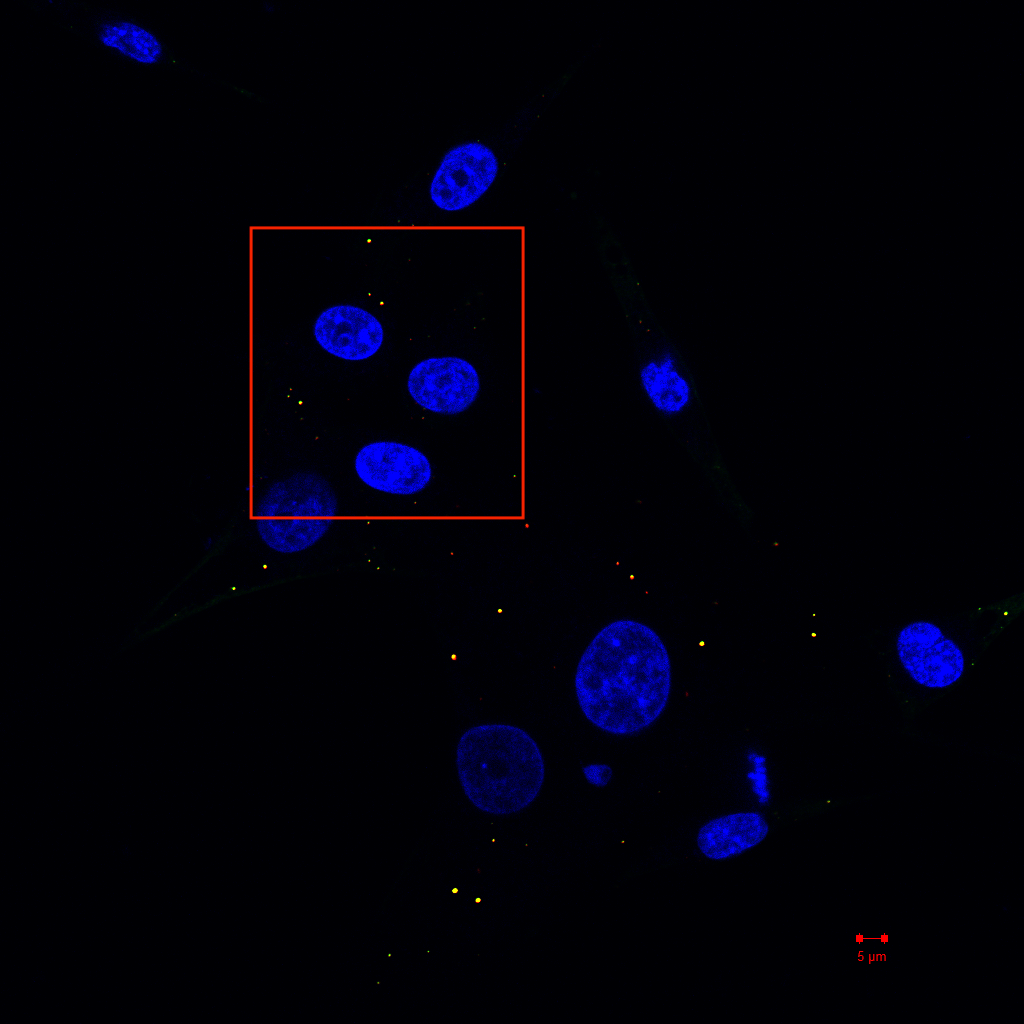

Supplement: Supplementary file 15 — Source data Fig. 5 [file 44319_2025_425_MOESM15_ESM.zip › Figure 5/5D/Patient-derived GBM cells (n┬░4) 30'Fsk.tiff]

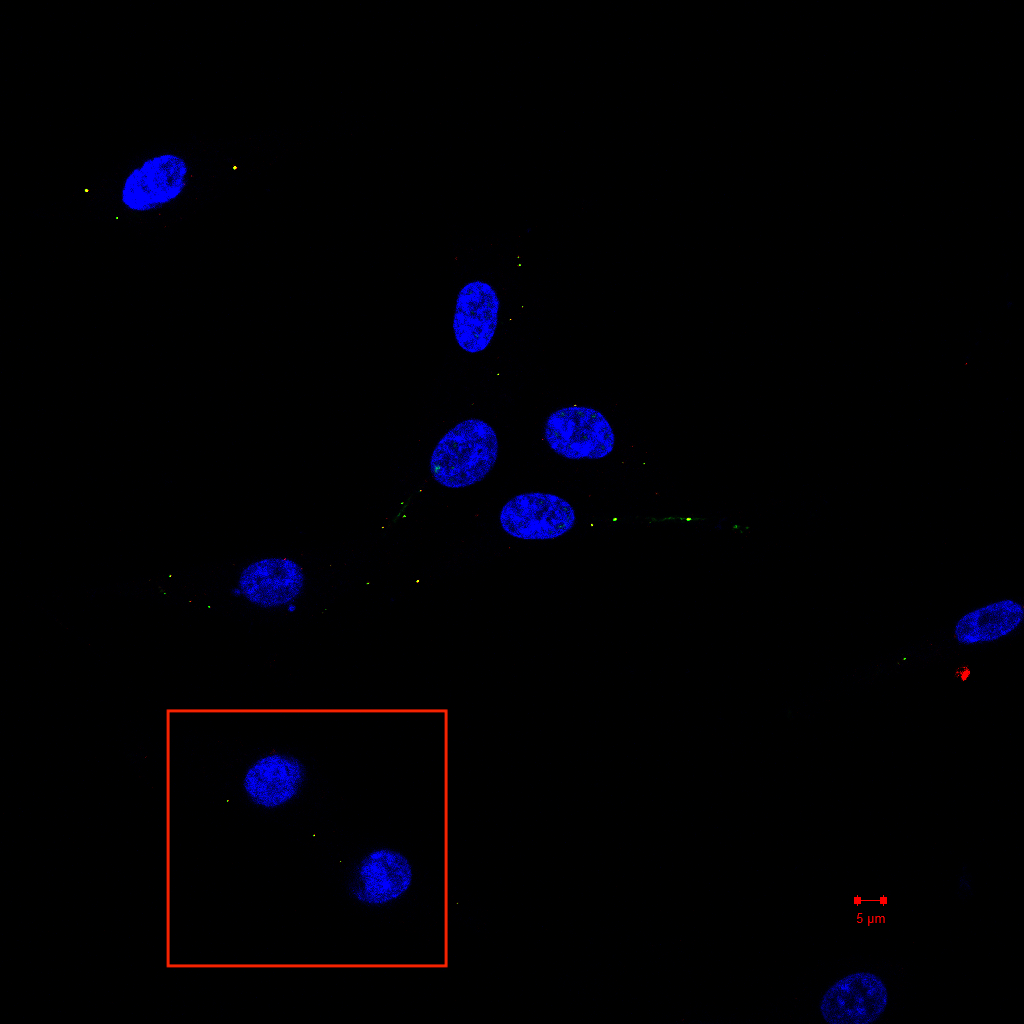

Supplement: Supplementary file 15 — Source data Fig. 5 [file 44319_2025_425_MOESM15_ESM.zip › Figure 5/5D/Patient-derived GBM cells (n┬░4) -.tiff]

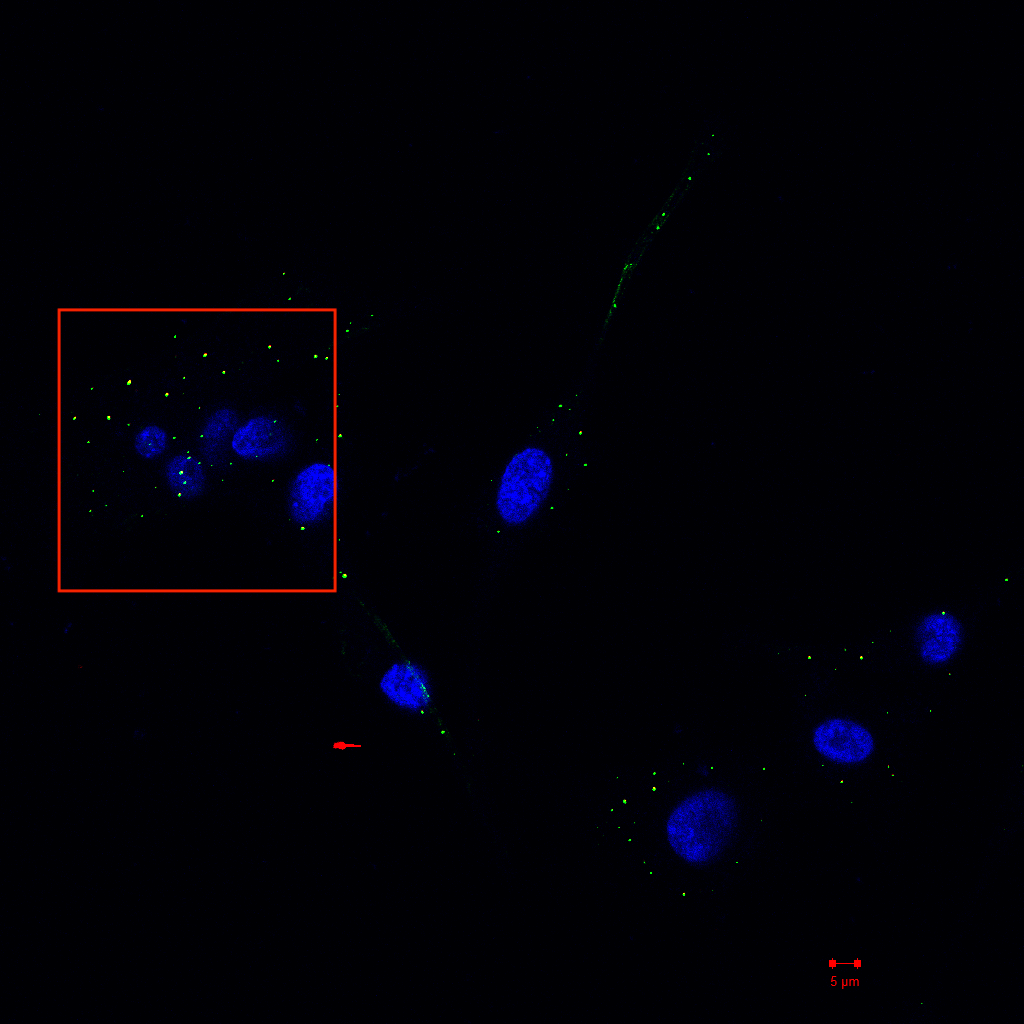

Supplement: Supplementary file 15 — Source data Fig. 5 [file 44319_2025_425_MOESM15_ESM.zip › Figure 5/5D/Patient-derived GBM cells (n┬░4) 60' Fsk.tiff]

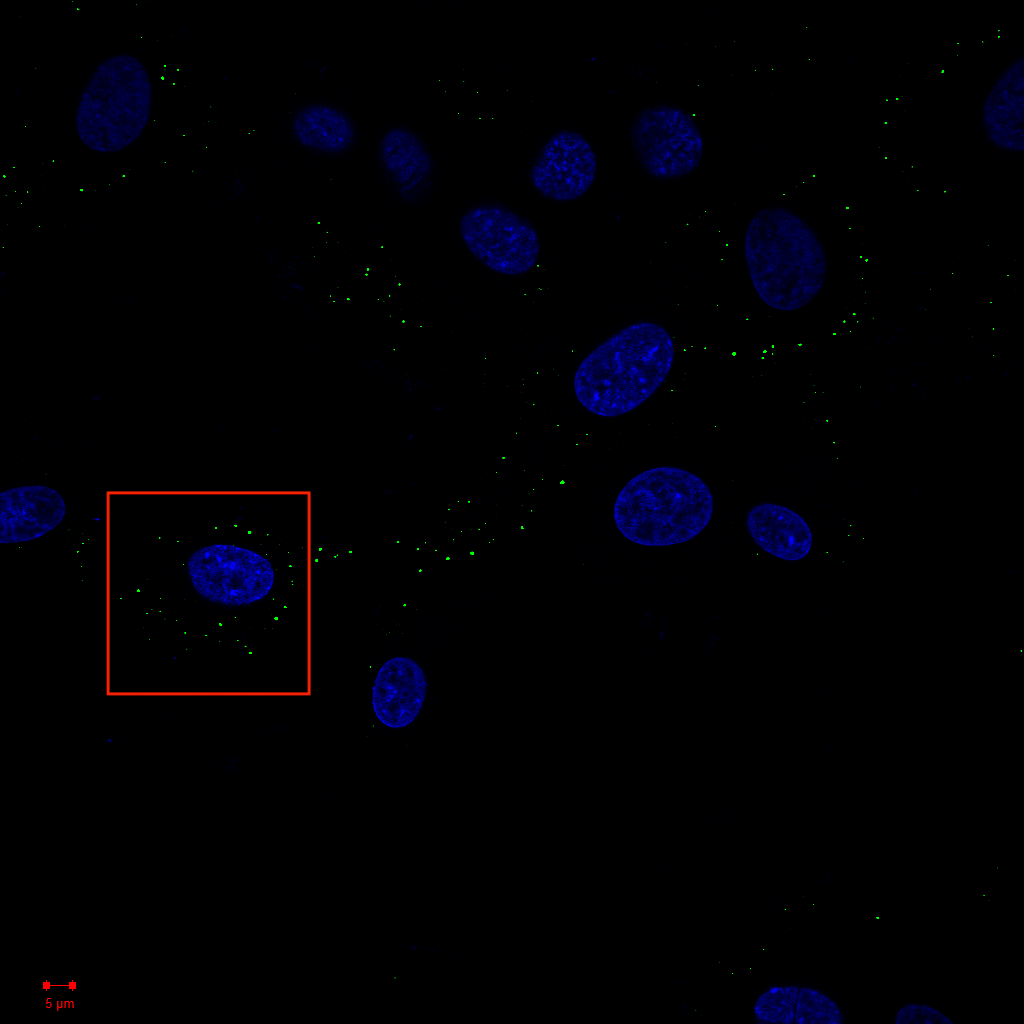

Supplement: Supplementary file 16 — Source data Fig. 6 [file 44319_2025_425_MOESM16_ESM.zip › Figure 6/6A/WT 30' Fsk.tiff]

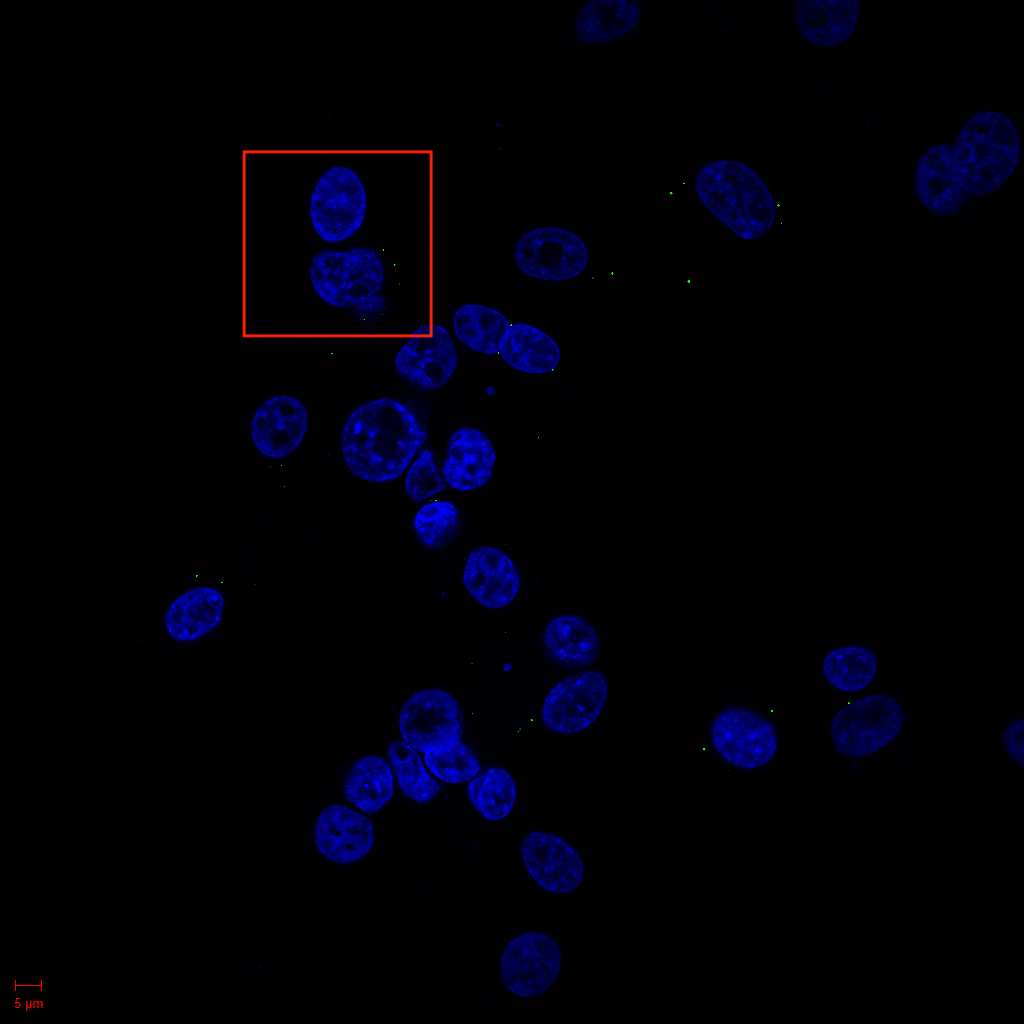

Supplement: Supplementary file 16 — Source data Fig. 6 [file 44319_2025_425_MOESM16_ESM.zip › Figure 6/6A/praja2KO -.tiff]

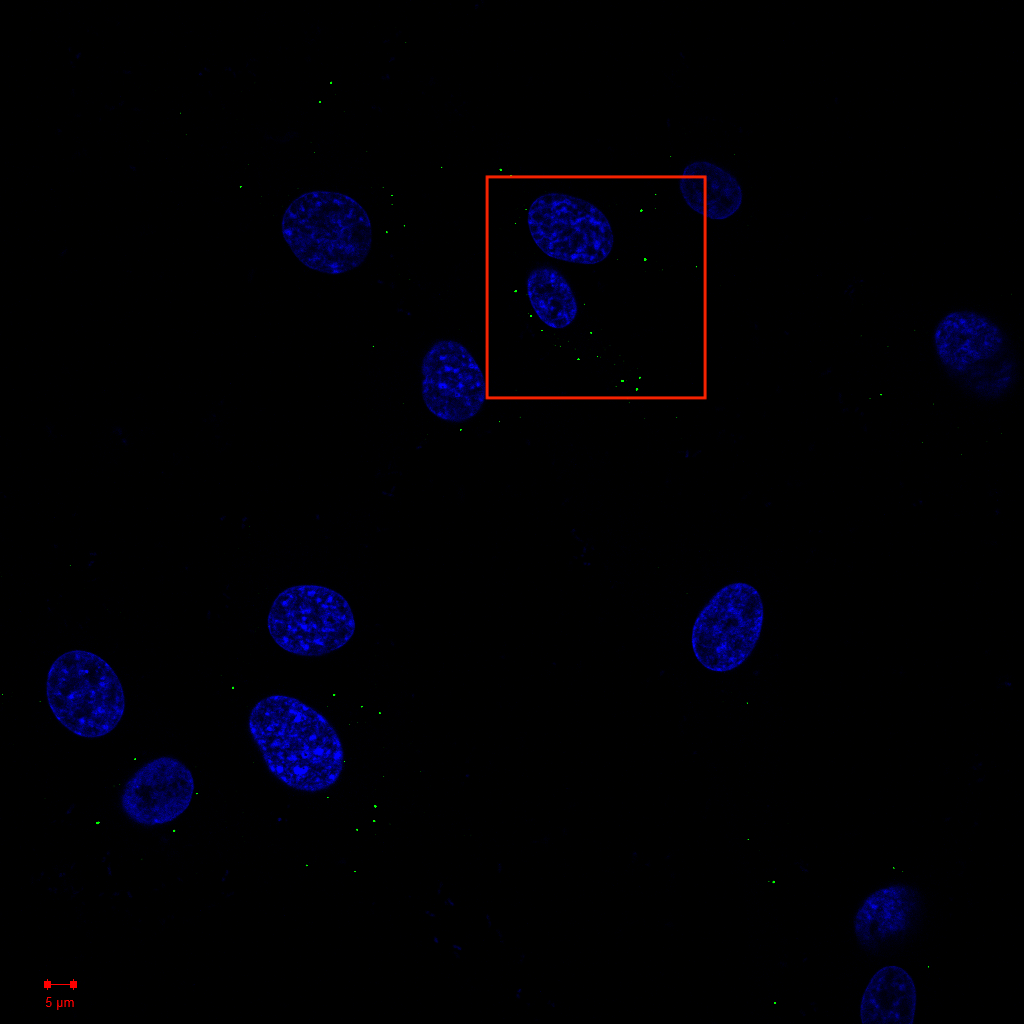

Supplement: Supplementary file 16 — Source data Fig. 6 [file 44319_2025_425_MOESM16_ESM.zip › Figure 6/6A/WT 60' Fsk.tiff]

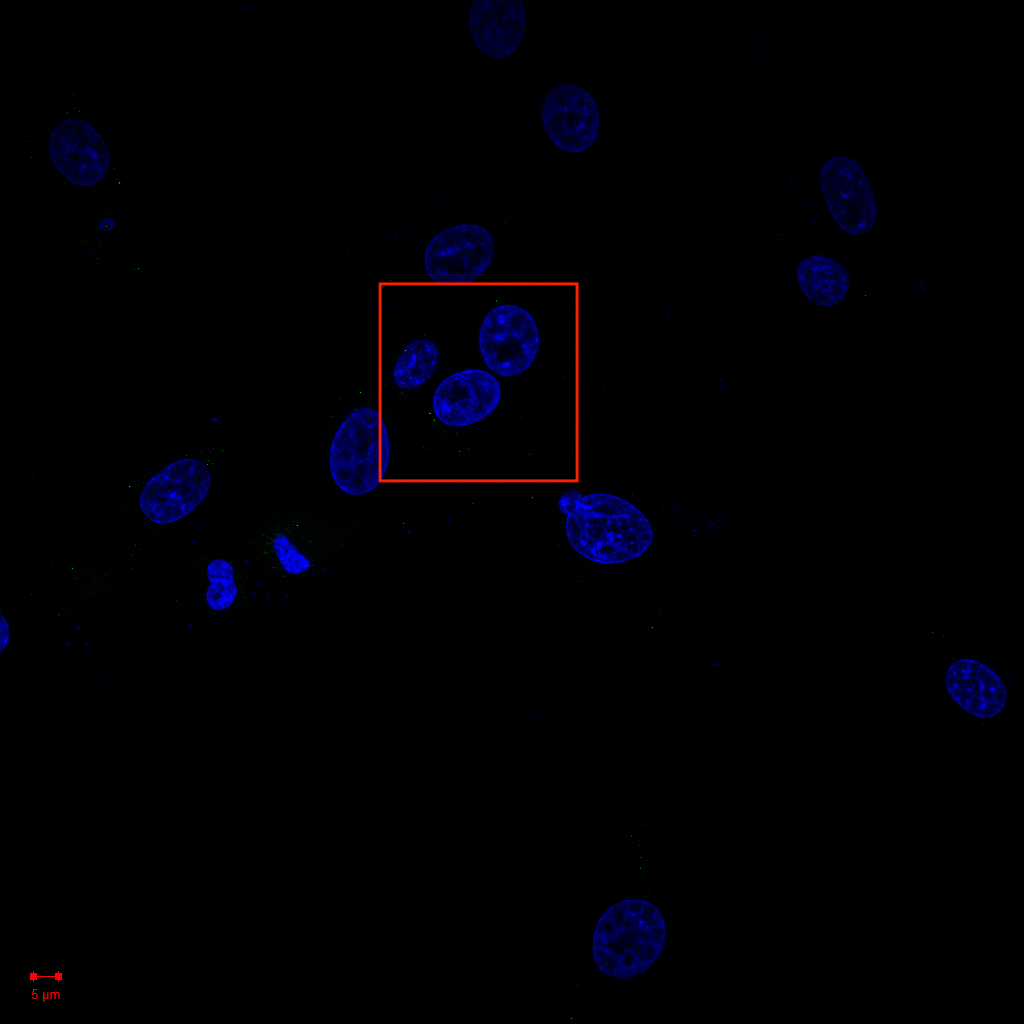

Supplement: Supplementary file 16 — Source data Fig. 6 [file 44319_2025_425_MOESM16_ESM.zip › Figure 6/6A/praja2KO 60' Fsk.tiff]

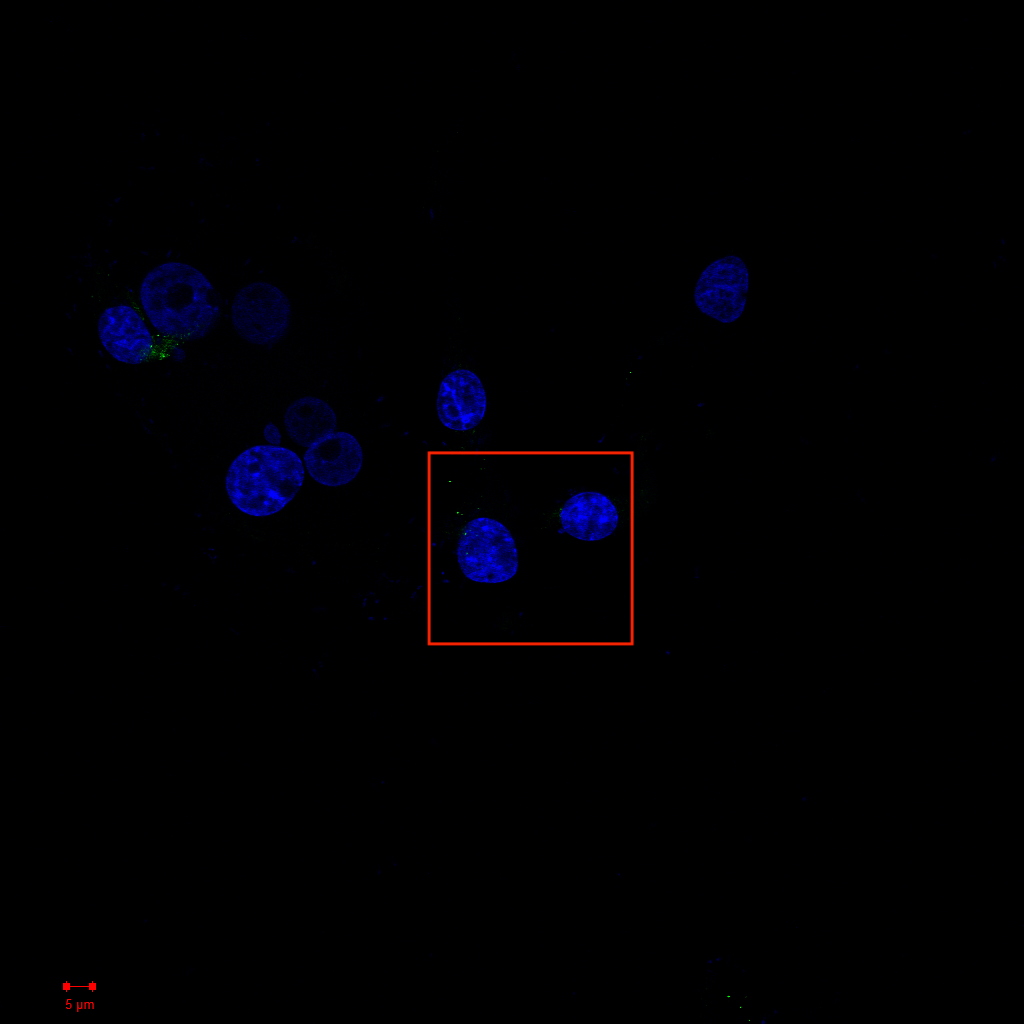

Supplement: Supplementary file 16 — Source data Fig. 6 [file 44319_2025_425_MOESM16_ESM.zip › Figure 6/6A/praja2KO 30' Fsk.tiff]

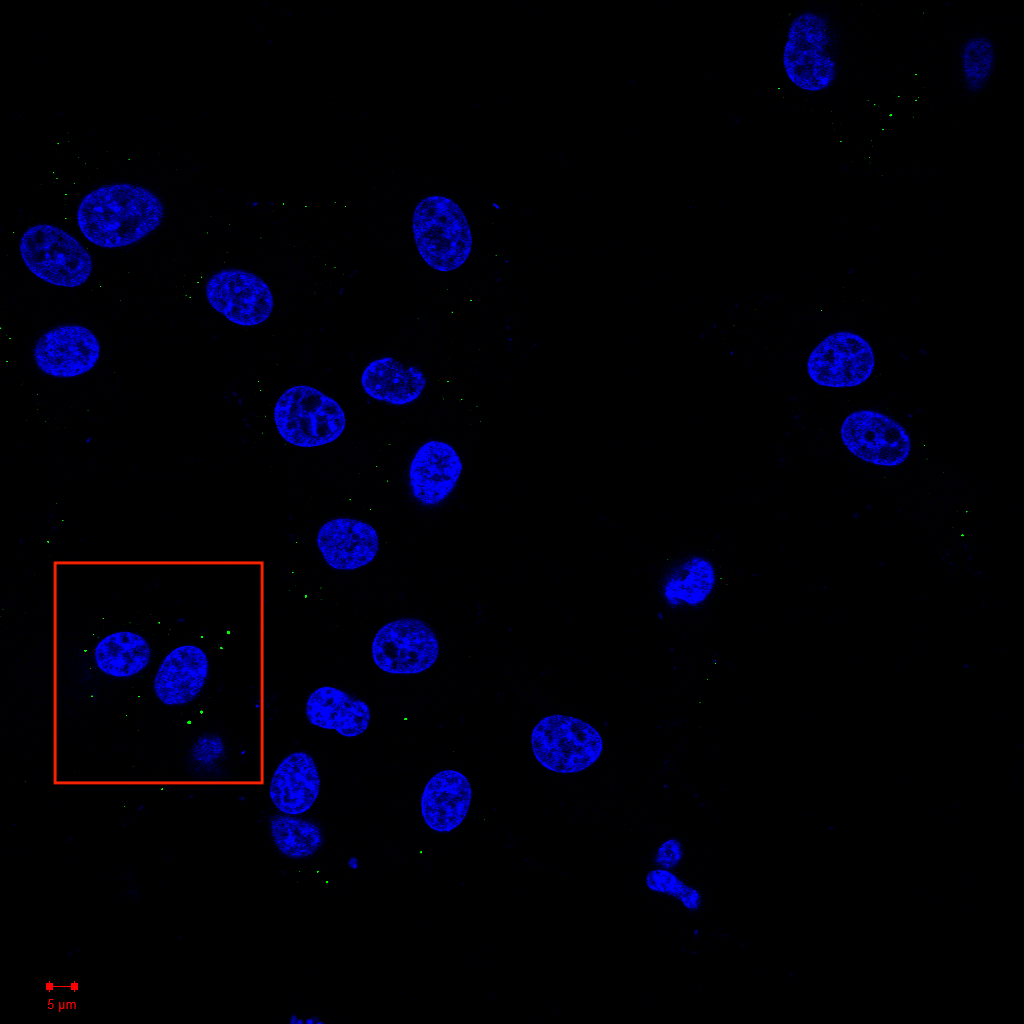

Supplement: Supplementary file 16 — Source data Fig. 6 [file 44319_2025_425_MOESM16_ESM.zip › Figure 6/6A/praja2KO+flag-praja2 30' Fsk.tiff]

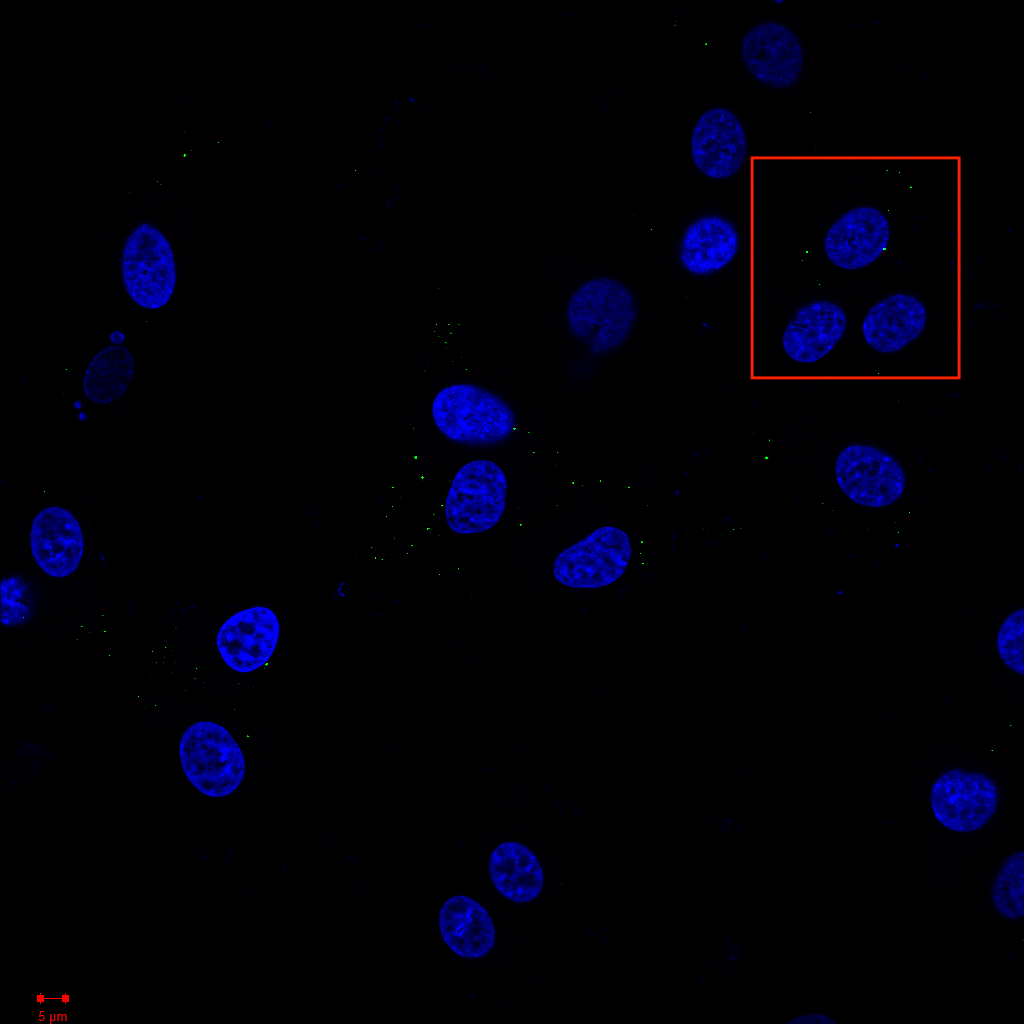

Supplement: Supplementary file 16 — Source data Fig. 6 [file 44319_2025_425_MOESM16_ESM.zip › Figure 6/6A/praja2KO+flag-praja2.tiff]

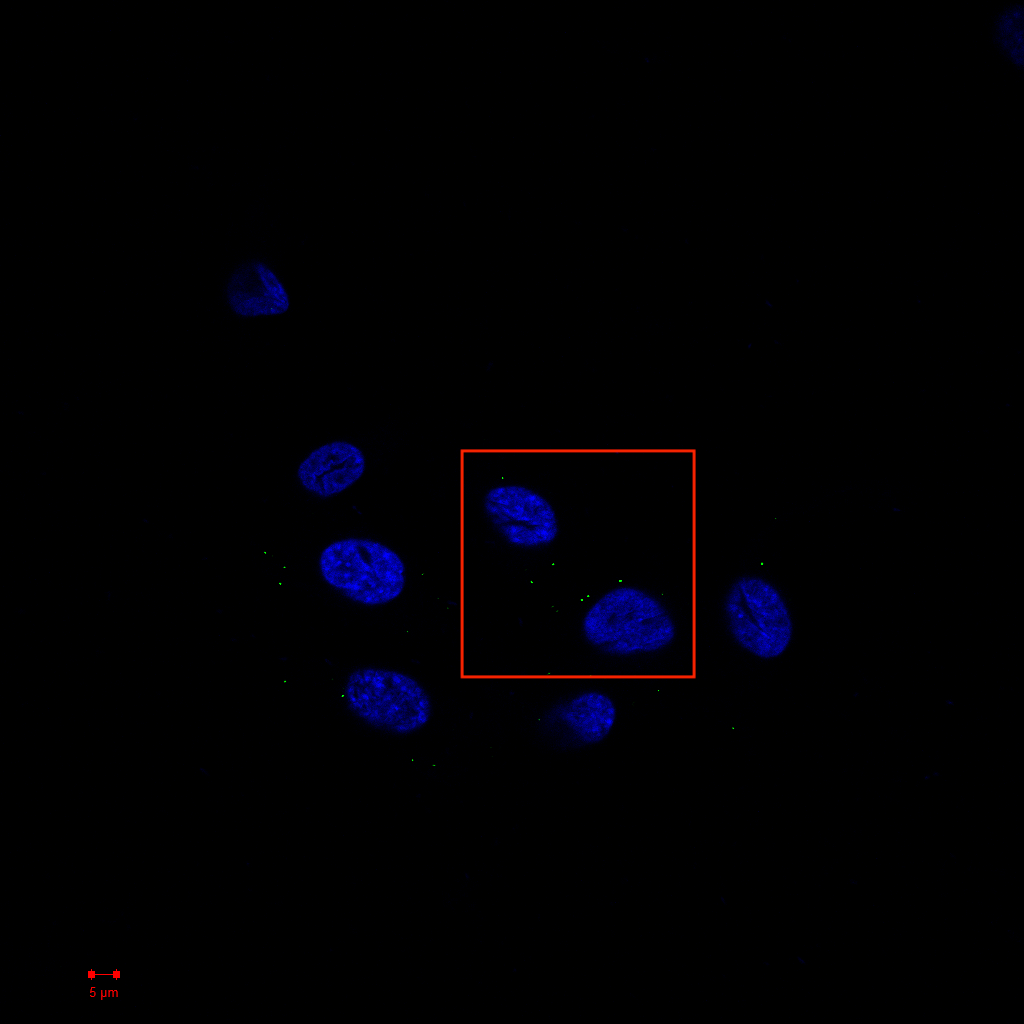

Supplement: Supplementary file 16 — Source data Fig. 6 [file 44319_2025_425_MOESM16_ESM.zip › Figure 6/6A/WT -.tiff]

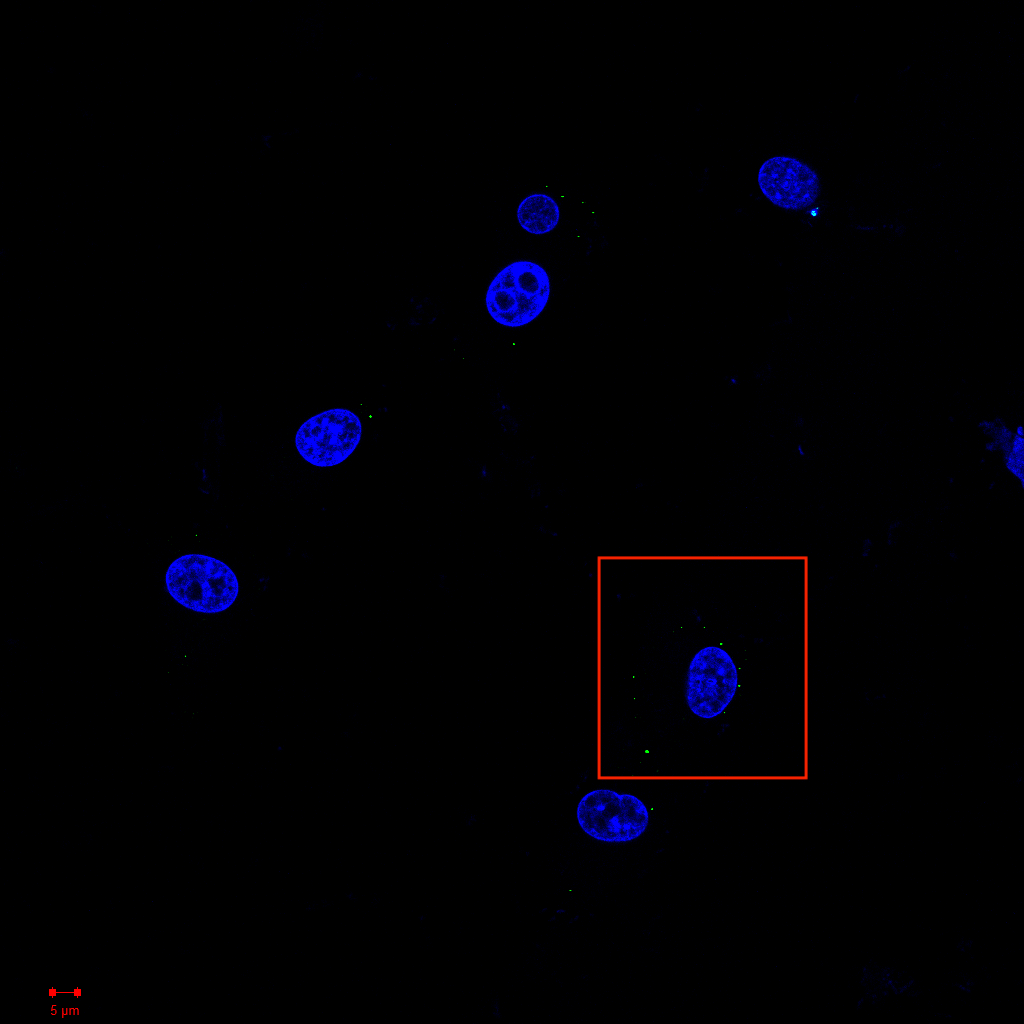

Supplement: Supplementary file 16 — Source data Fig. 6 [file 44319_2025_425_MOESM16_ESM.zip › Figure 6/6A/praja2KO+flag-praja2 60' Fsk.tiff]

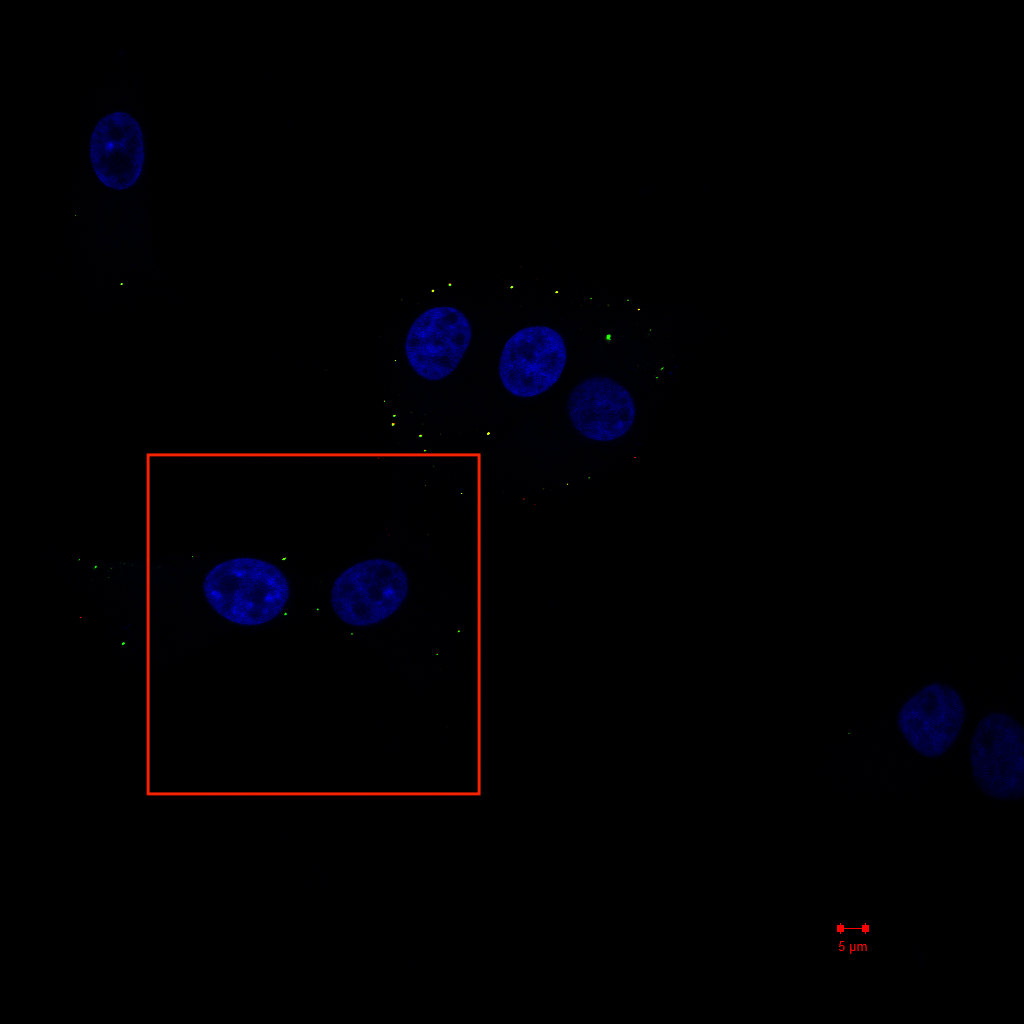

Supplement: Supplementary file 16 — Source data Fig. 6 [file 44319_2025_425_MOESM16_ESM.zip › Figure 6/6D/praja2KO -.tiff]

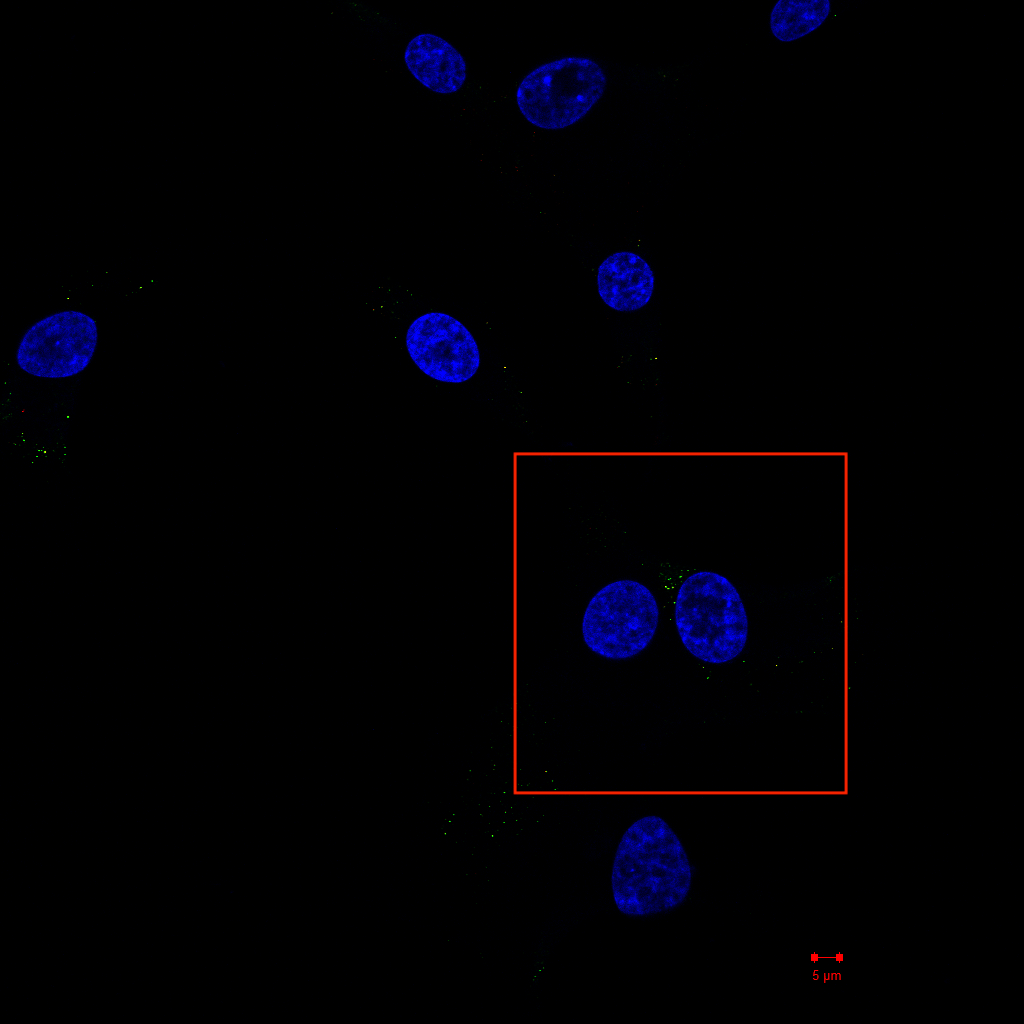

Supplement: Supplementary file 16 — Source data Fig. 6 [file 44319_2025_425_MOESM16_ESM.zip › Figure 6/6D/praja2KO 60' PGE2.tiff]

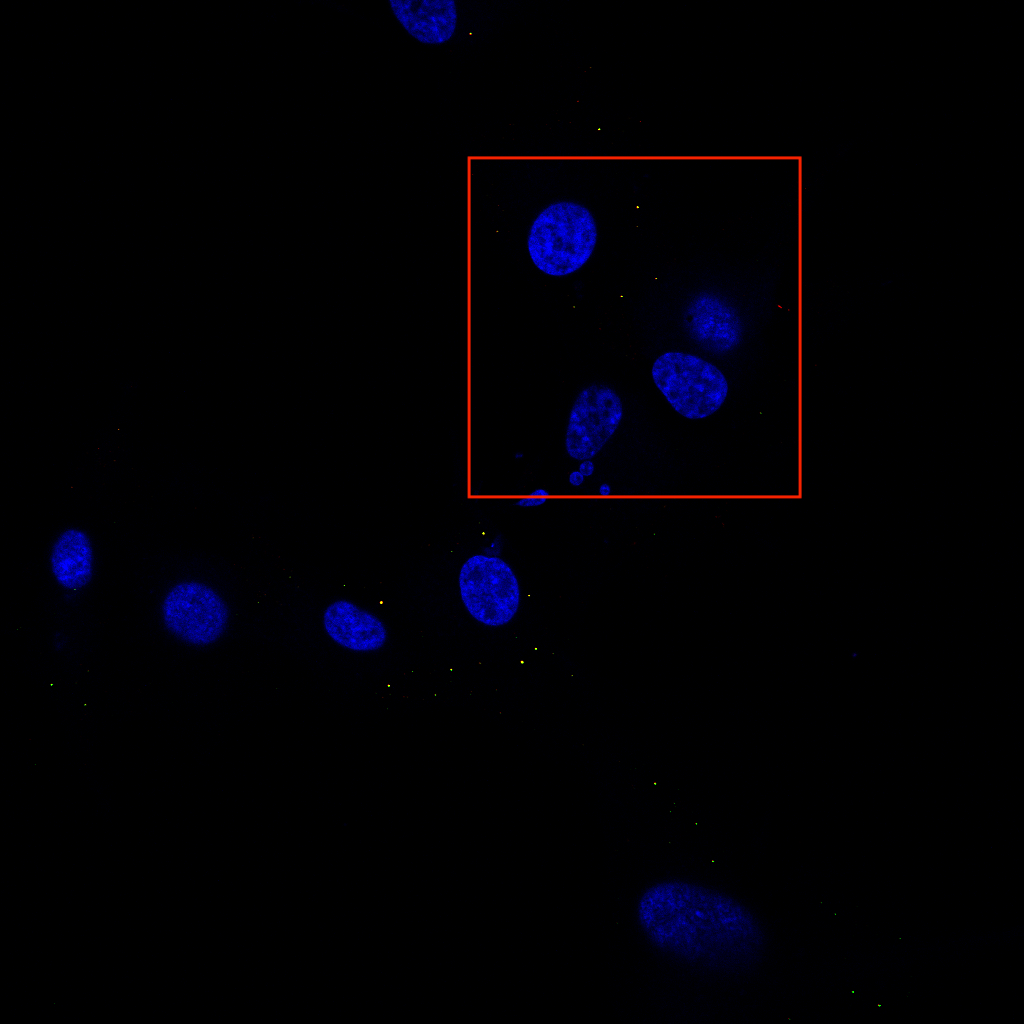

Supplement: Supplementary file 16 — Source data Fig. 6 [file 44319_2025_425_MOESM16_ESM.zip › Figure 6/6D/praja2KO 30' PGE2.tiff]

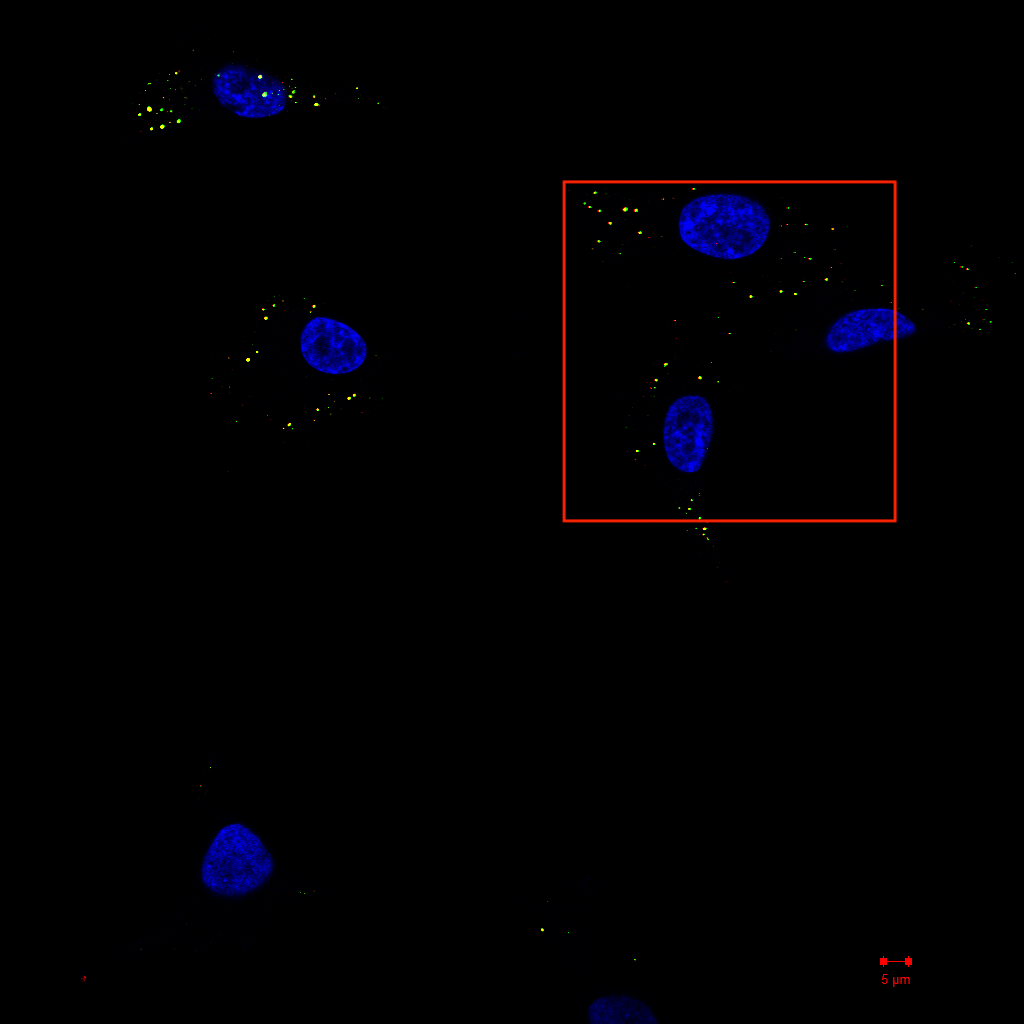

Supplement: Supplementary file 16 — Source data Fig. 6 [file 44319_2025_425_MOESM16_ESM.zip › Figure 6/6D/WT 60' PGE2.tiff]

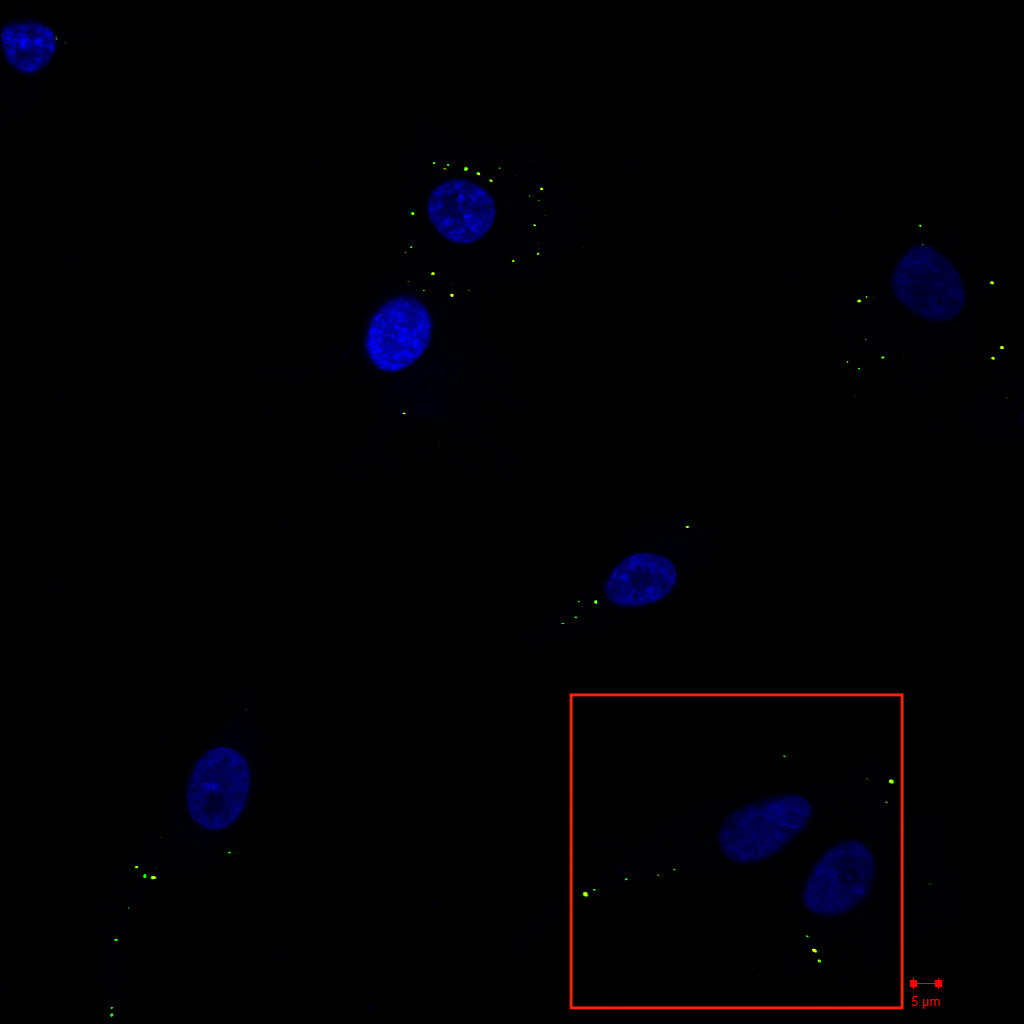

Supplement: Supplementary file 16 — Source data Fig. 6 [file 44319_2025_425_MOESM16_ESM.zip › Figure 6/6D/WT -.tiff]

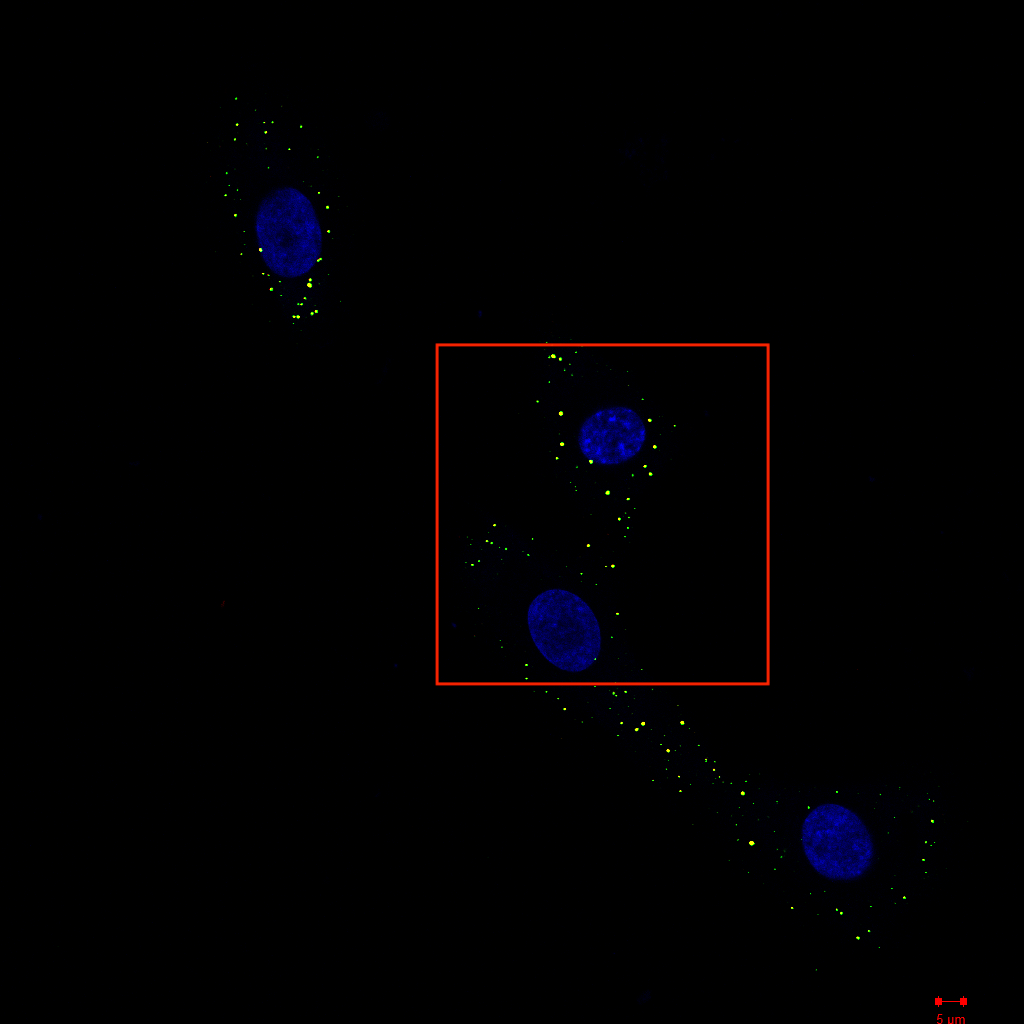

Supplement: Supplementary file 16 — Source data Fig. 6 [file 44319_2025_425_MOESM16_ESM.zip › Figure 6/6D/WT 30' PGE2.tiff]

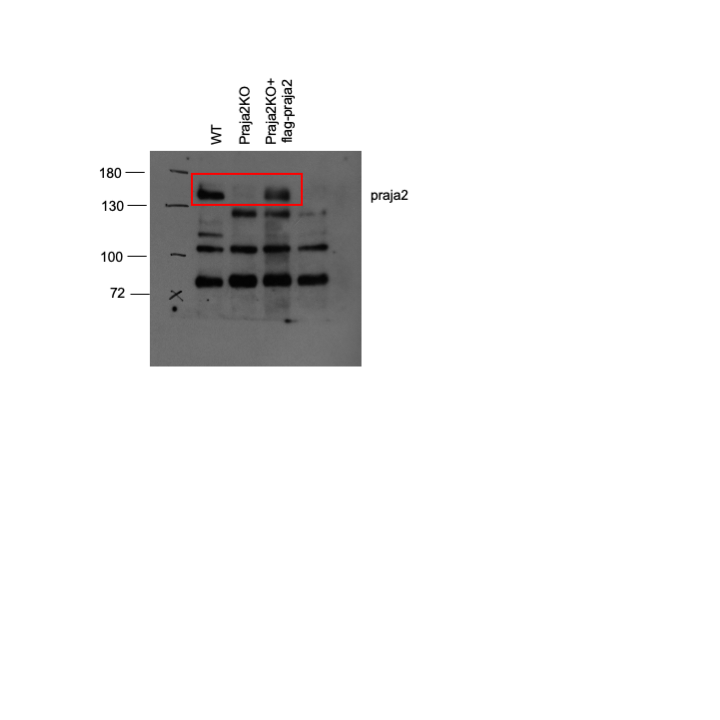

Supplement: Supplementary file 16 — Source data Fig. 6 [file 44319_2025_425_MOESM16_ESM.zip › Figure 6/6C/praja2.tiff]

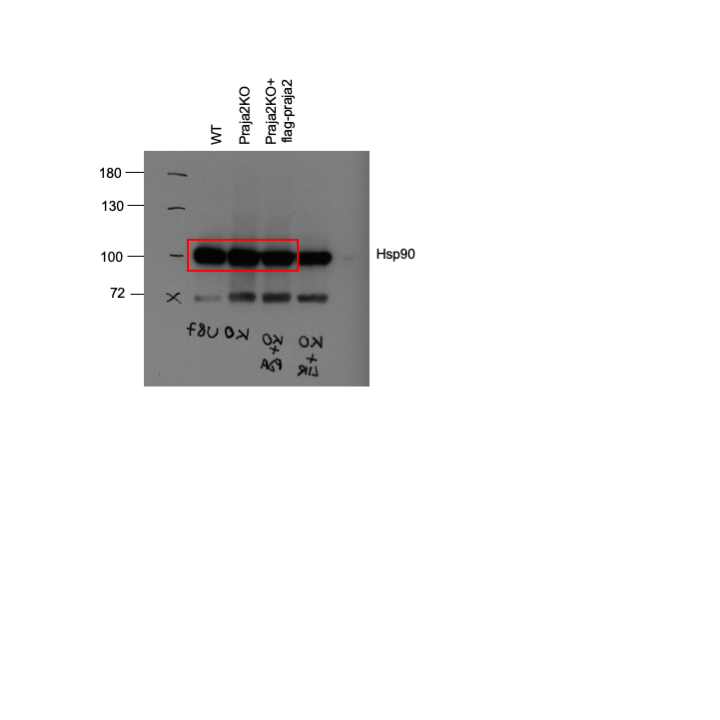

Supplement: Supplementary file 16 — Source data Fig. 6 [file 44319_2025_425_MOESM16_ESM.zip › Figure 6/6C/Hsp90.tiff]

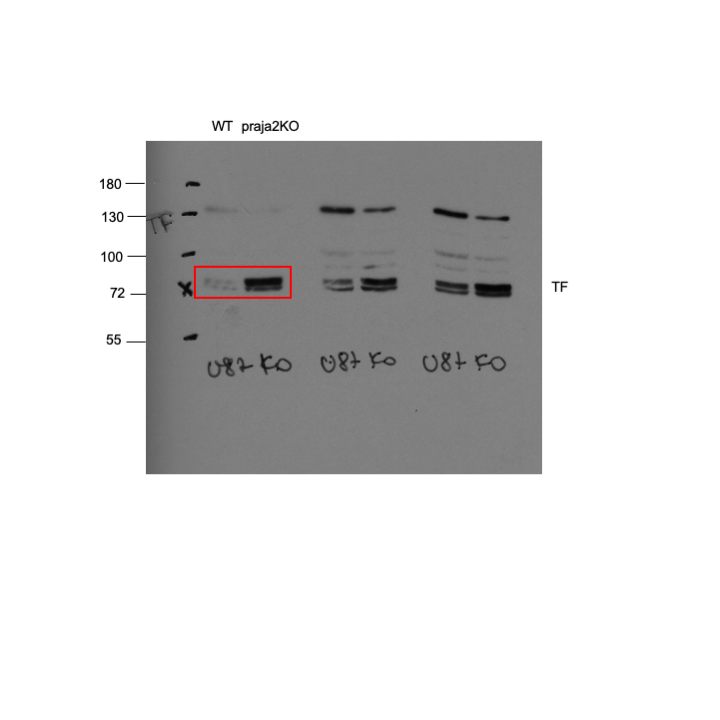

Supplement: Supplementary file 17 — Source data Fig. 7 [file 44319_2025_425_MOESM17_ESM.zip › Figure 7/7D/TF.tiff]

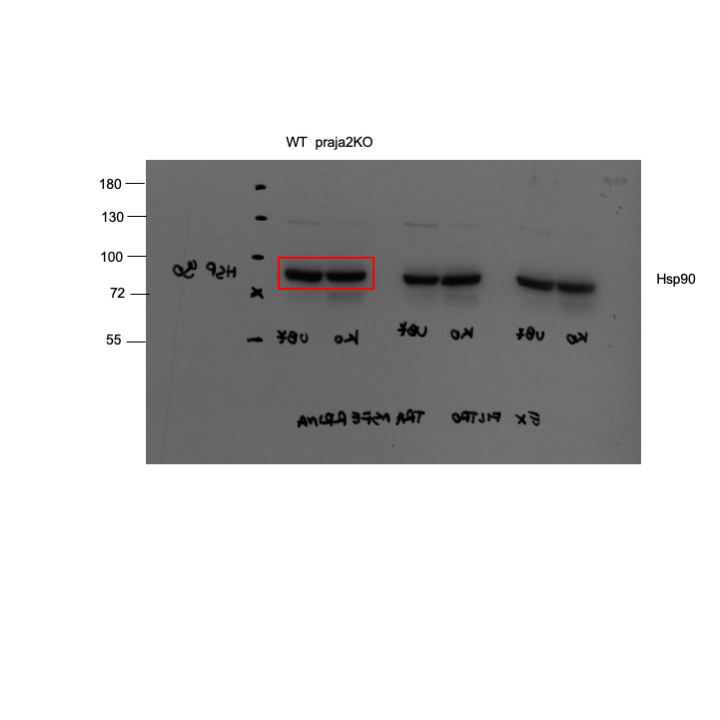

Supplement: Supplementary file 17 — Source data Fig. 7 [file 44319_2025_425_MOESM17_ESM.zip › Figure 7/7D/Hsp90.tiff]

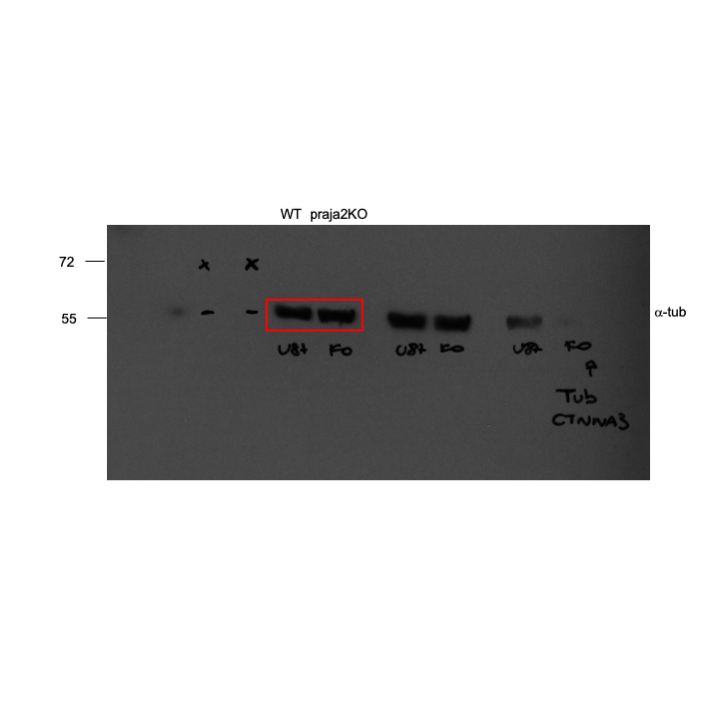

Supplement: Supplementary file 17 — Source data Fig. 7 [file 44319_2025_425_MOESM17_ESM.zip › Figure 7/7F/a-tub .tiff]

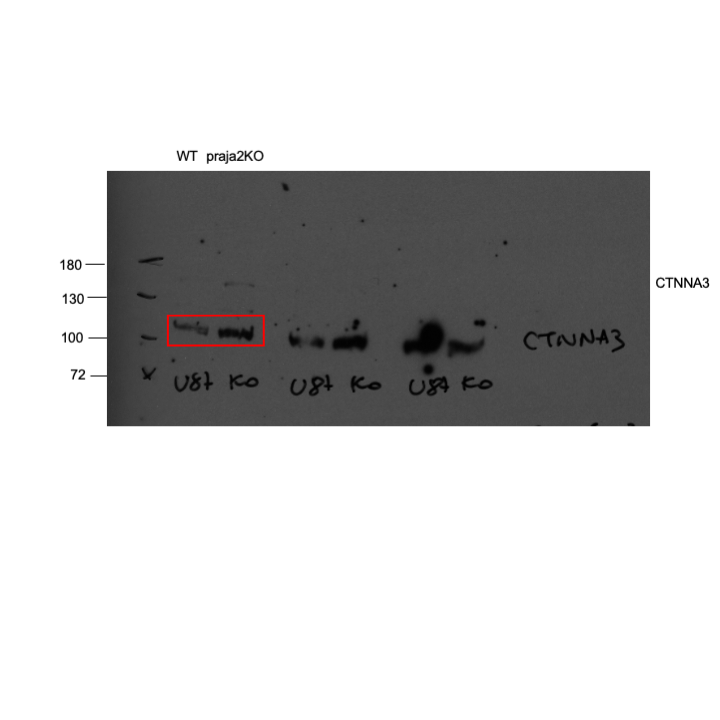

Supplement: Supplementary file 17 — Source data Fig. 7 [file 44319_2025_425_MOESM17_ESM.zip › Figure 7/7F/CTNNA3.tiff]

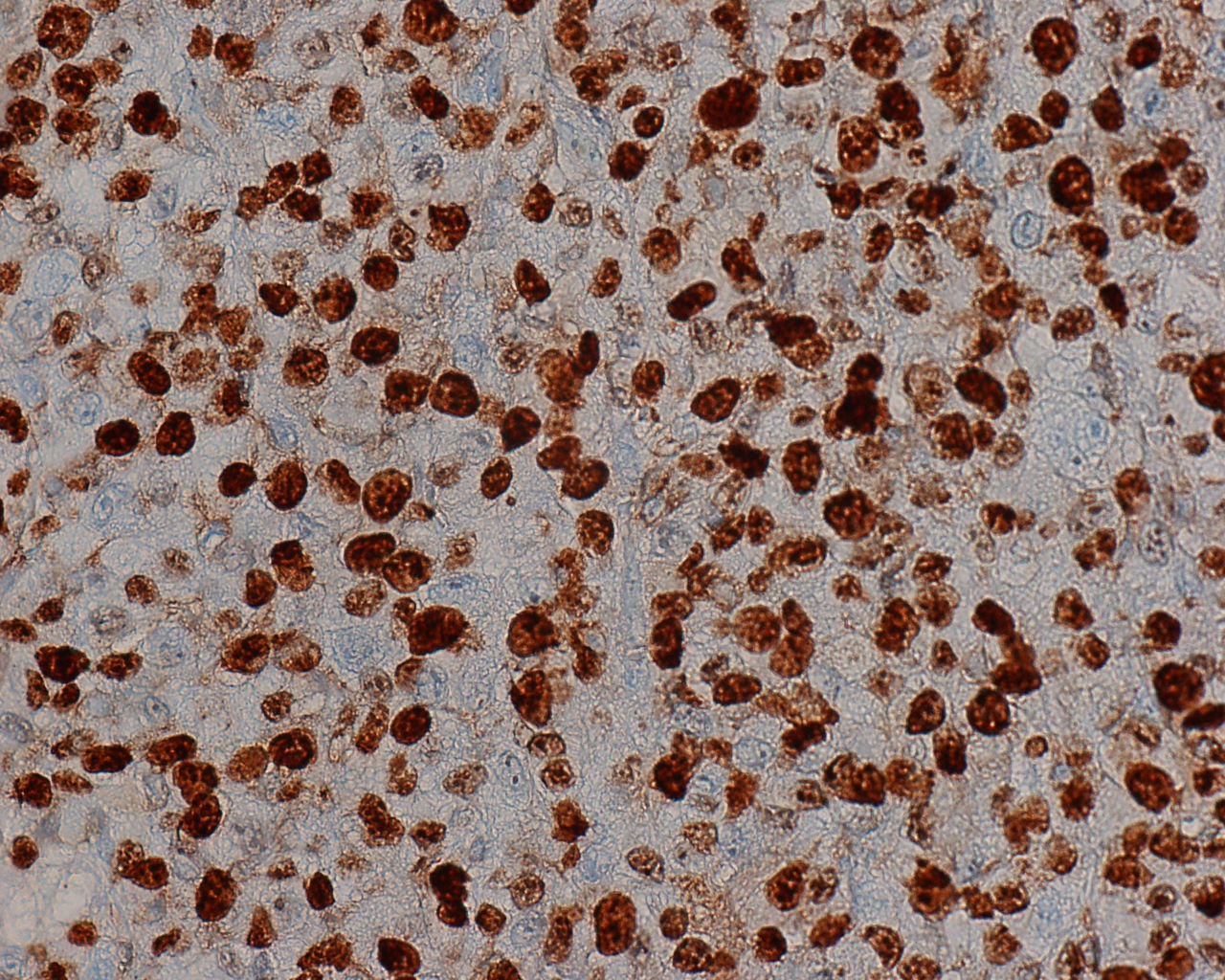

Supplement: Supplementary file 18 — Source data Fig. 8 [file 44319_2025_425_MOESM18_ESM.zip › Figure 8/8G/WT ki67.tif]

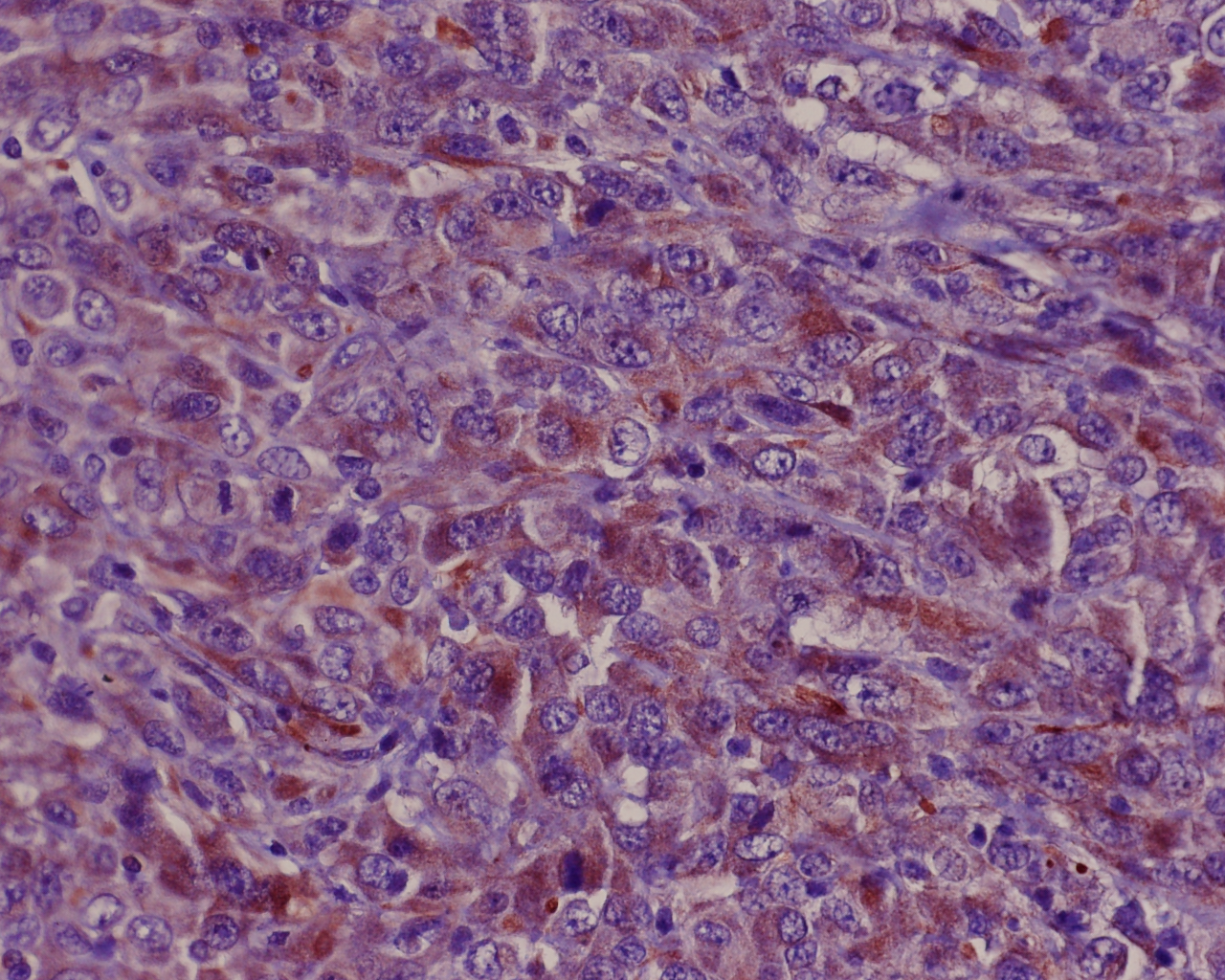

Supplement: Supplementary file 18 — Source data Fig. 8 [file 44319_2025_425_MOESM18_ESM.zip › Figure 8/8G/WT p53.tif]

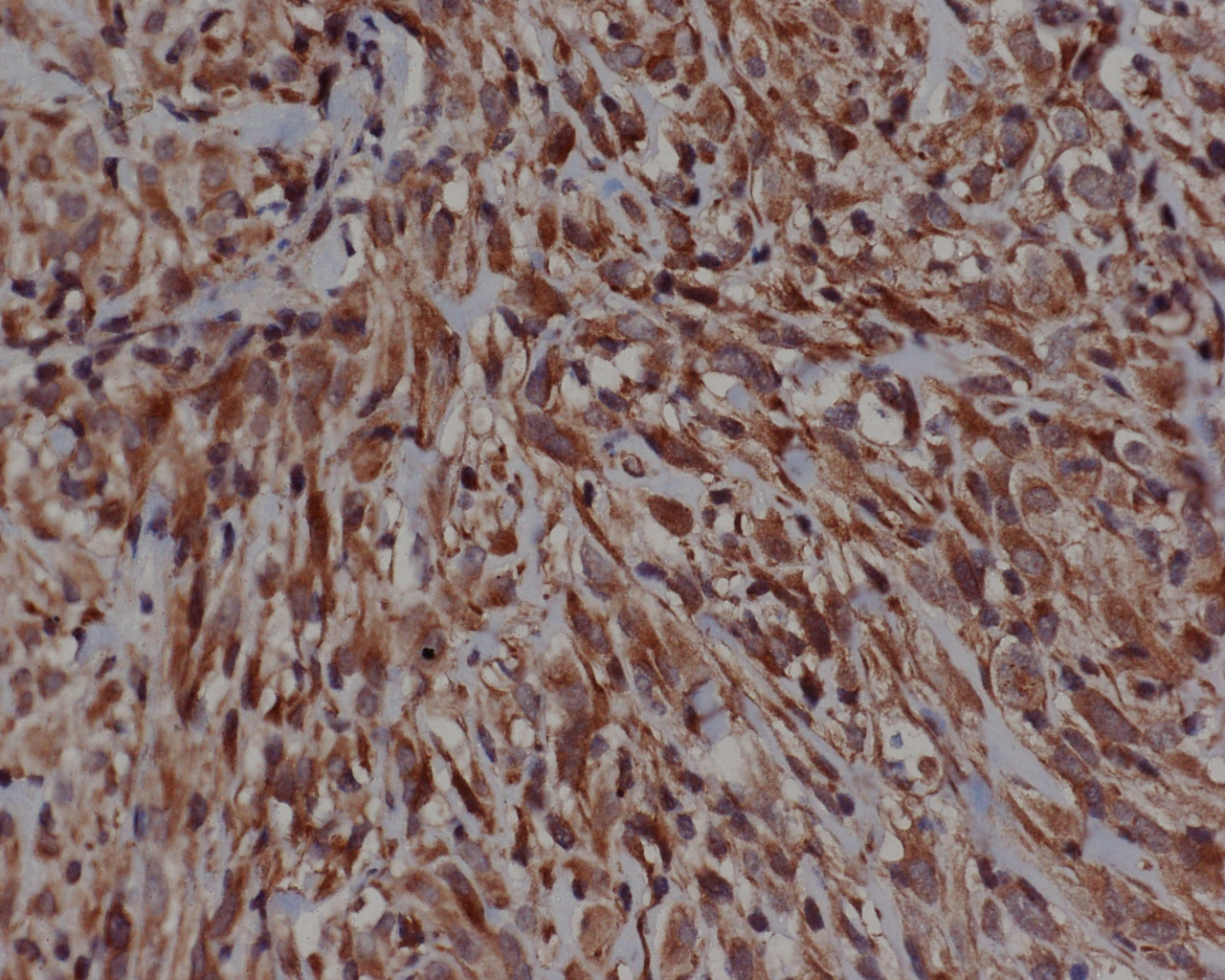

Supplement: Supplementary file 18 — Source data Fig. 8 [file 44319_2025_425_MOESM18_ESM.zip › Figure 8/8G/praja2KO p53.tif]

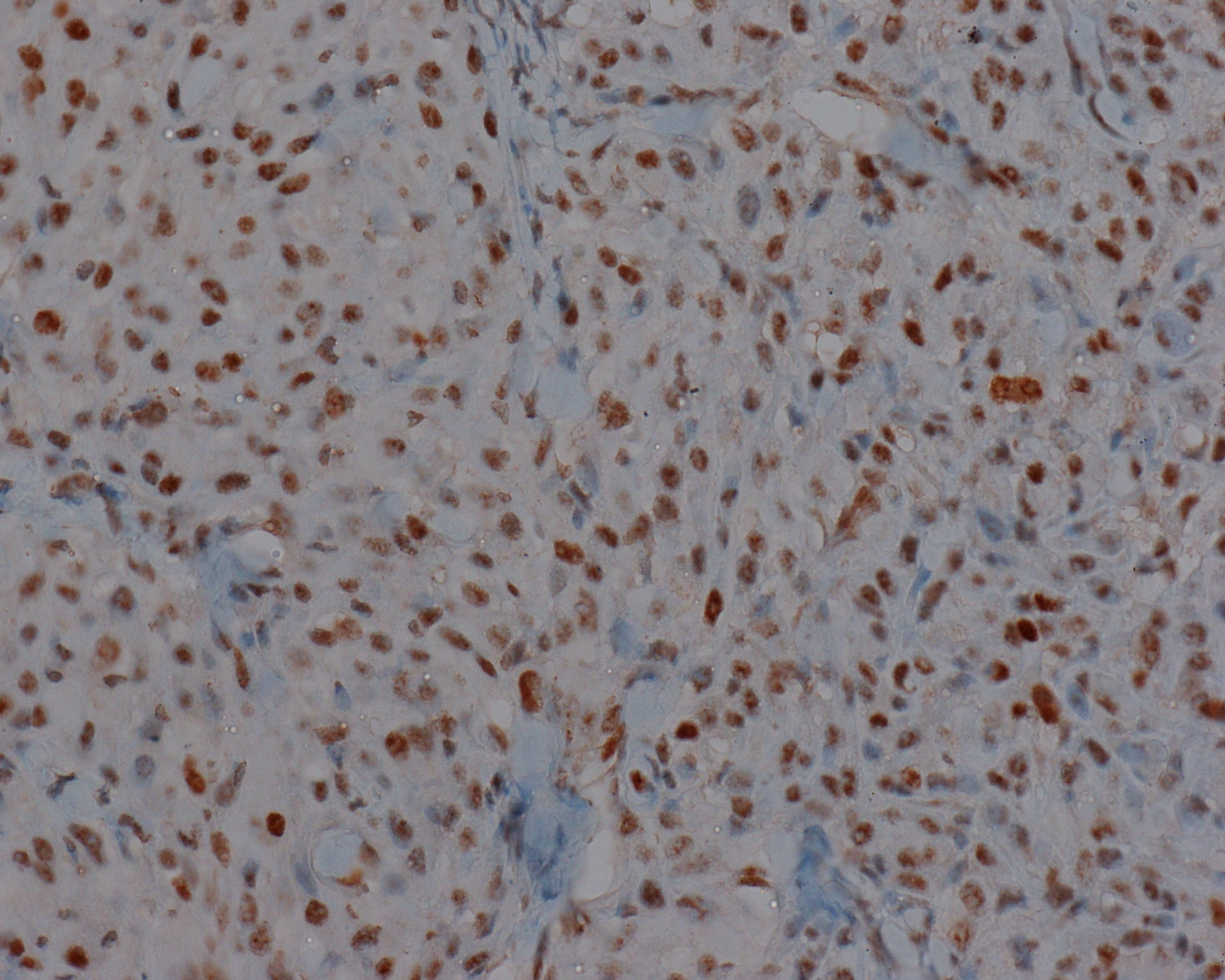

Supplement: Supplementary file 18 — Source data Fig. 8 [file 44319_2025_425_MOESM18_ESM.zip › Figure 8/8G/praja2KO p21.tif]

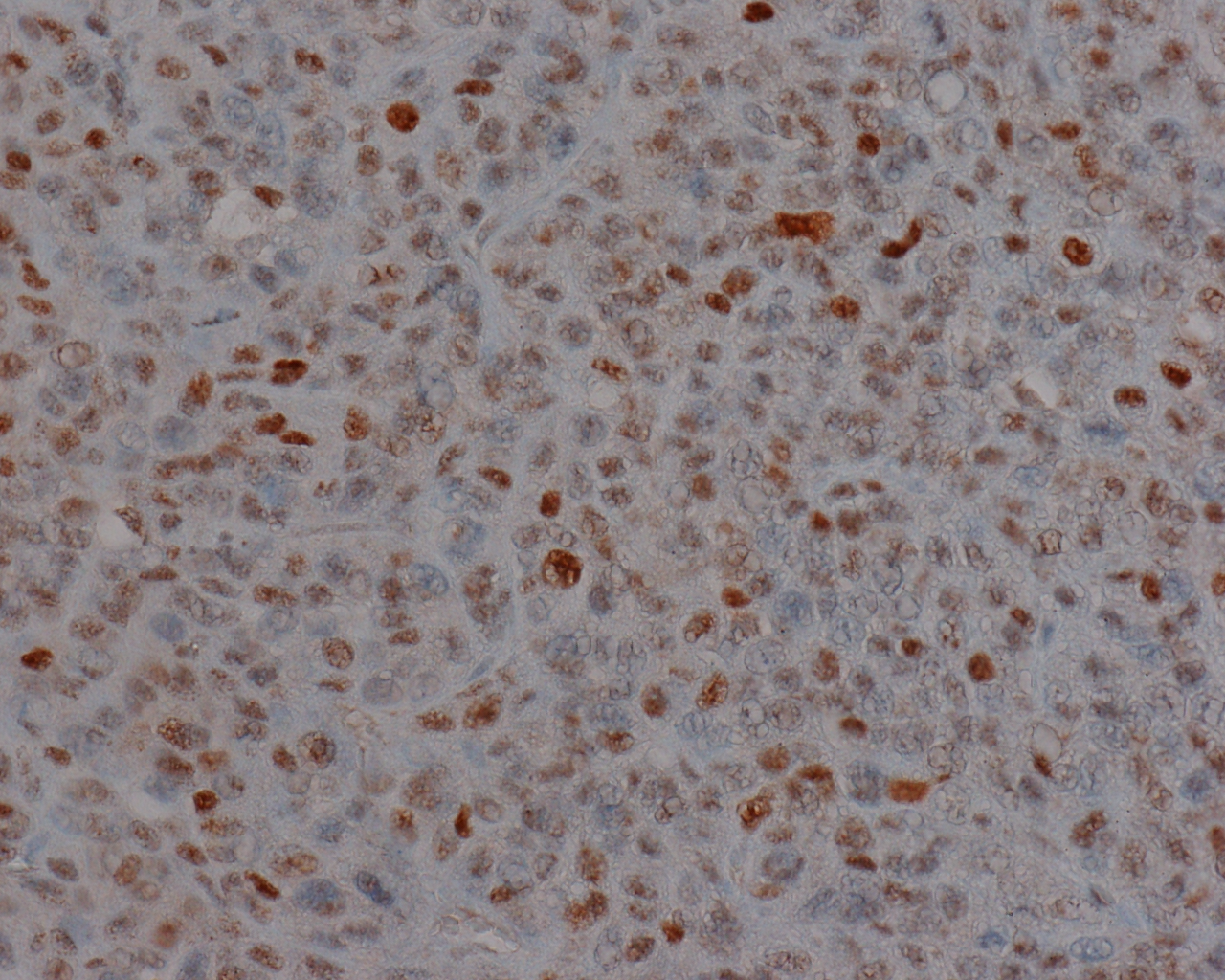

Supplement: Supplementary file 18 — Source data Fig. 8 [file 44319_2025_425_MOESM18_ESM.zip › Figure 8/8G/WT p21.tif]

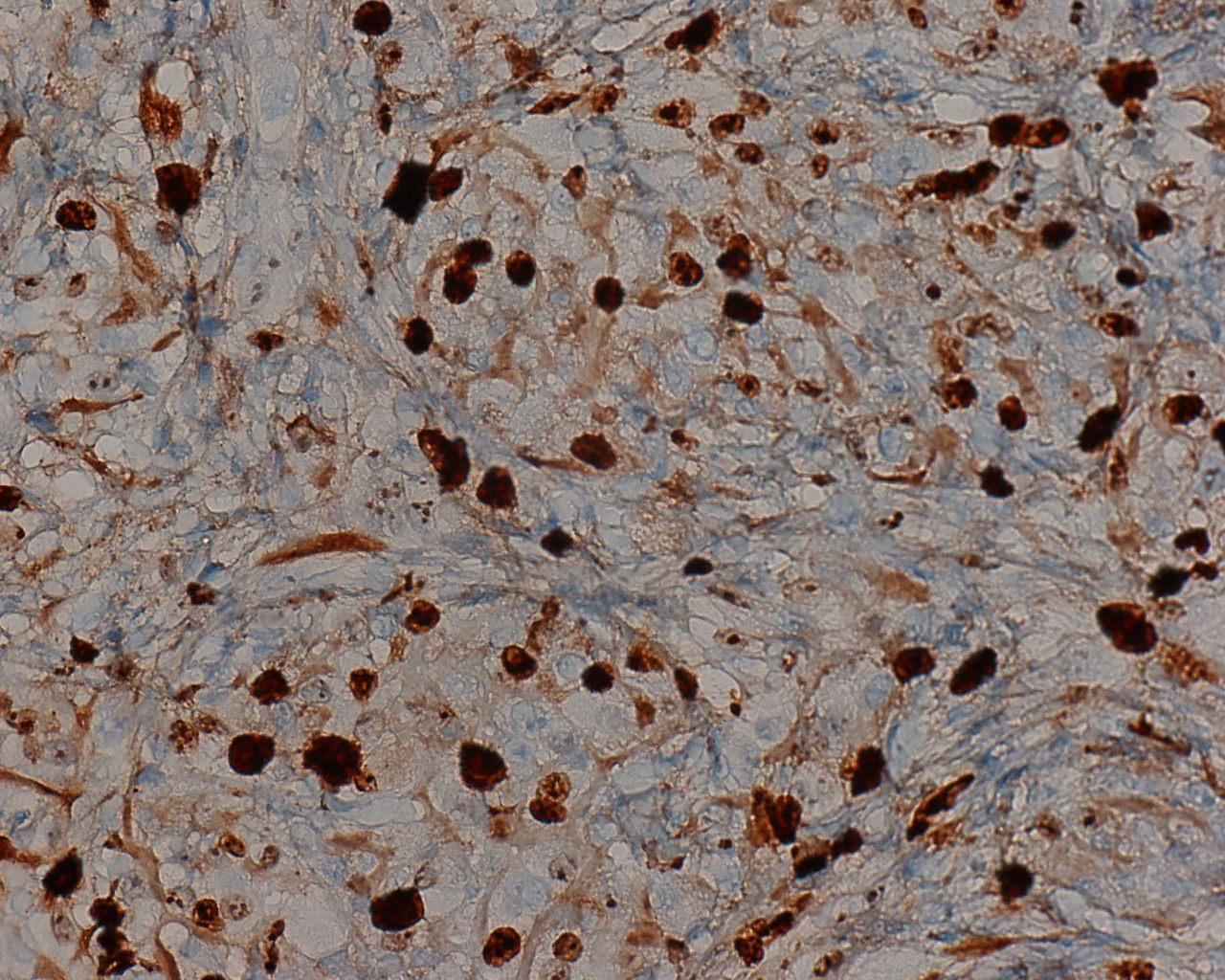

Supplement: Supplementary file 18 — Source data Fig. 8 [file 44319_2025_425_MOESM18_ESM.zip › Figure 8/8G/praja2KO ki67.tif]

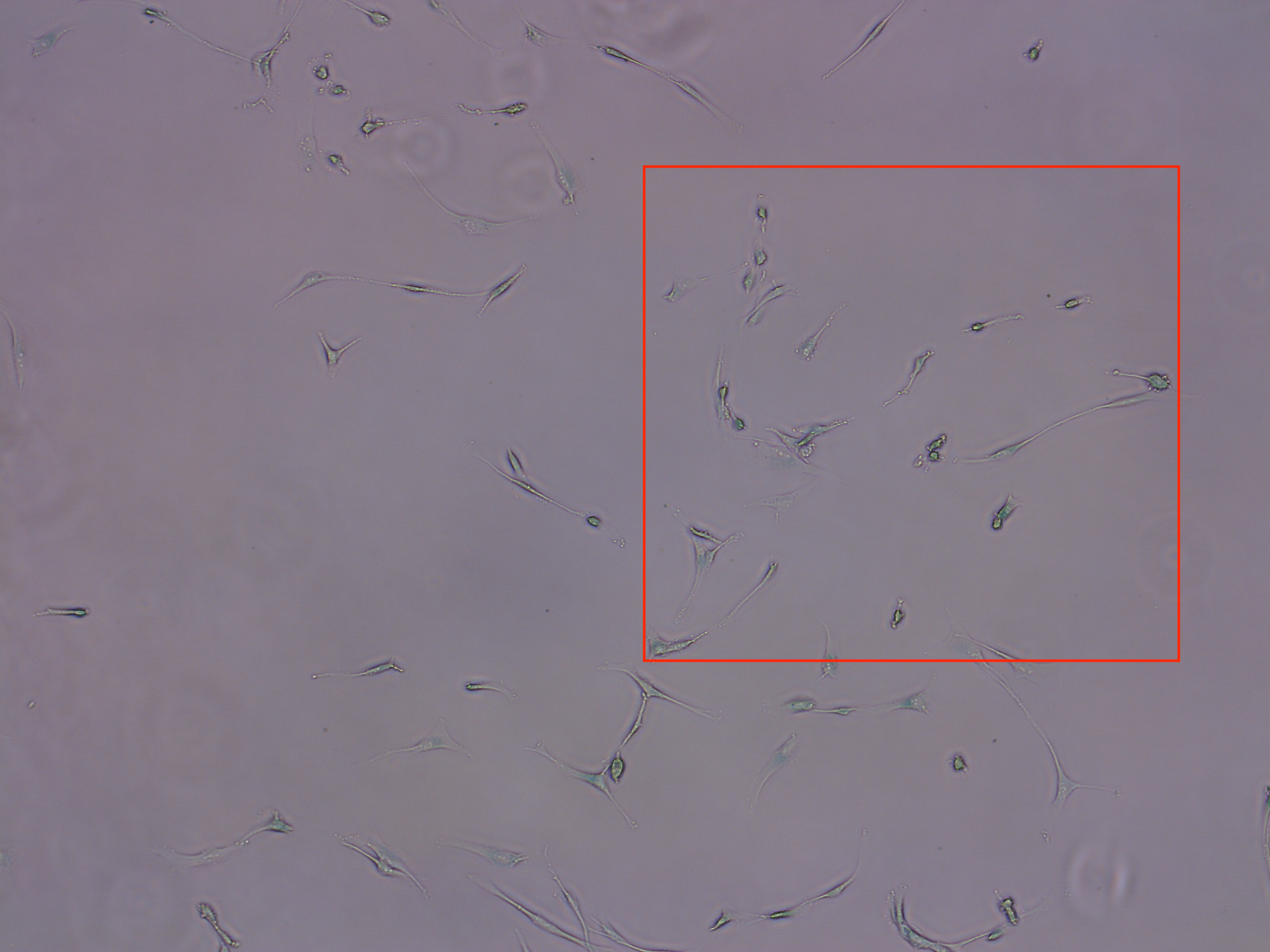

Supplement: Supplementary file 18 — Source data Fig. 8 [file 44319_2025_425_MOESM18_ESM.zip › Figure 8/8A/WT.tiff]

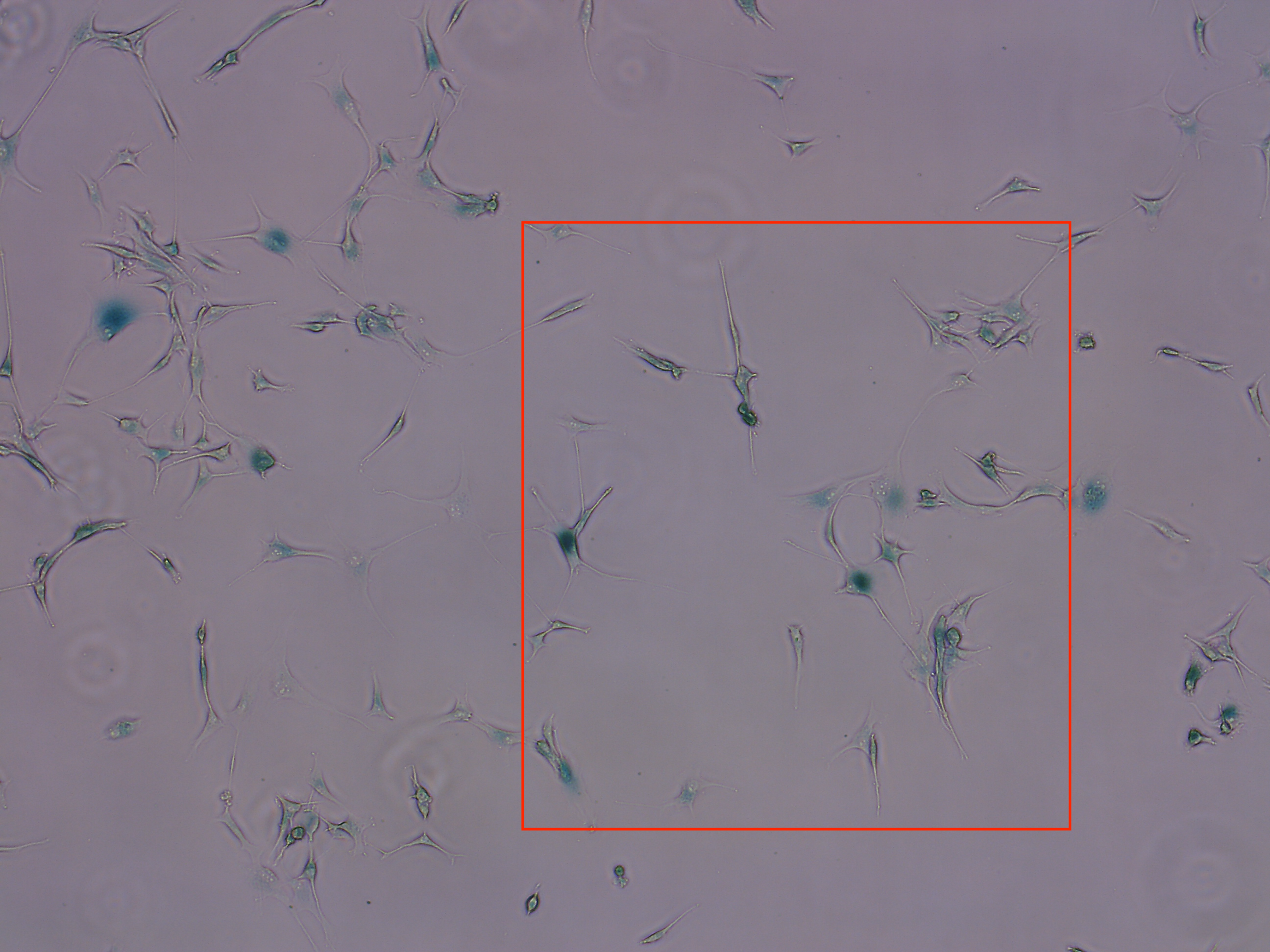

Supplement: Supplementary file 18 — Source data Fig. 8 [file 44319_2025_425_MOESM18_ESM.zip › Figure 8/8A/praja2KO.tiff]

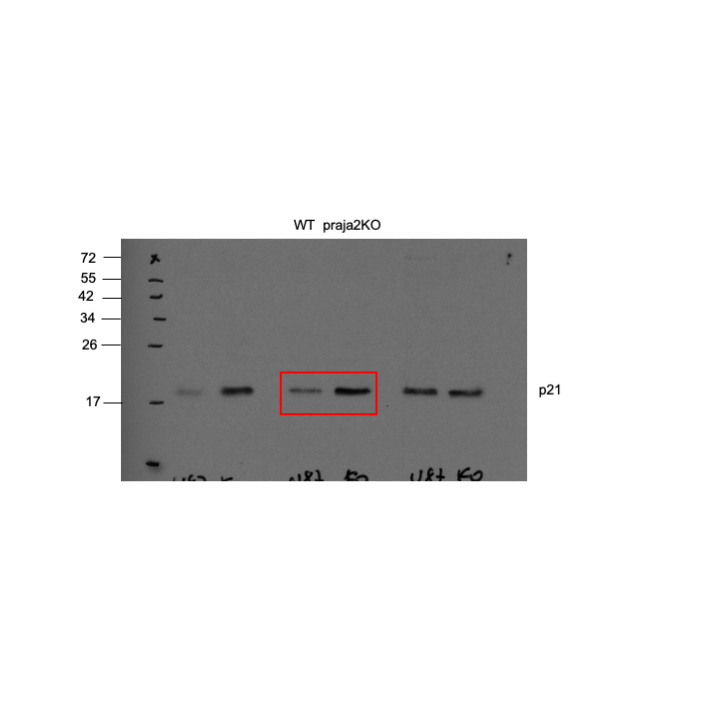

Supplement: Supplementary file 18 — Source data Fig. 8 [file 44319_2025_425_MOESM18_ESM.zip › Figure 8/8C/p21.tiff]

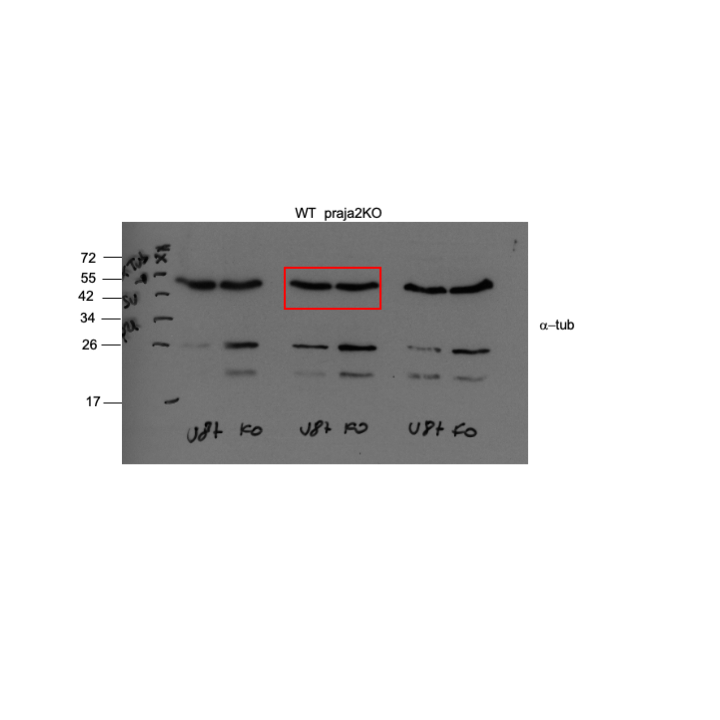

Supplement: Supplementary file 18 — Source data Fig. 8 [file 44319_2025_425_MOESM18_ESM.zip › Figure 8/8C/a-tub.tiff]

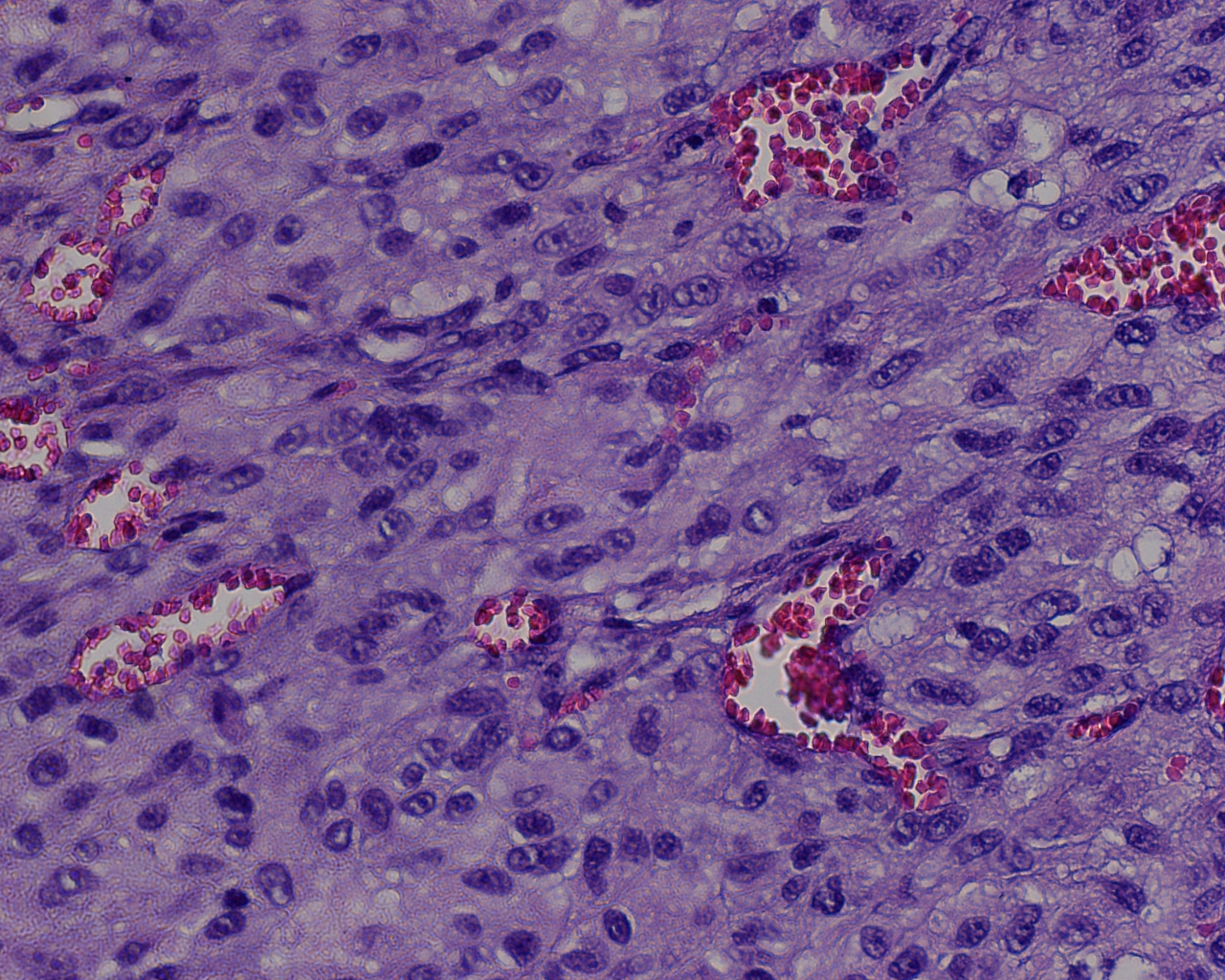

Supplement: Supplementary file 18 — Source data Fig. 8 [file 44319_2025_425_MOESM18_ESM.zip › Figure 8/8E/H:E WT.tif]

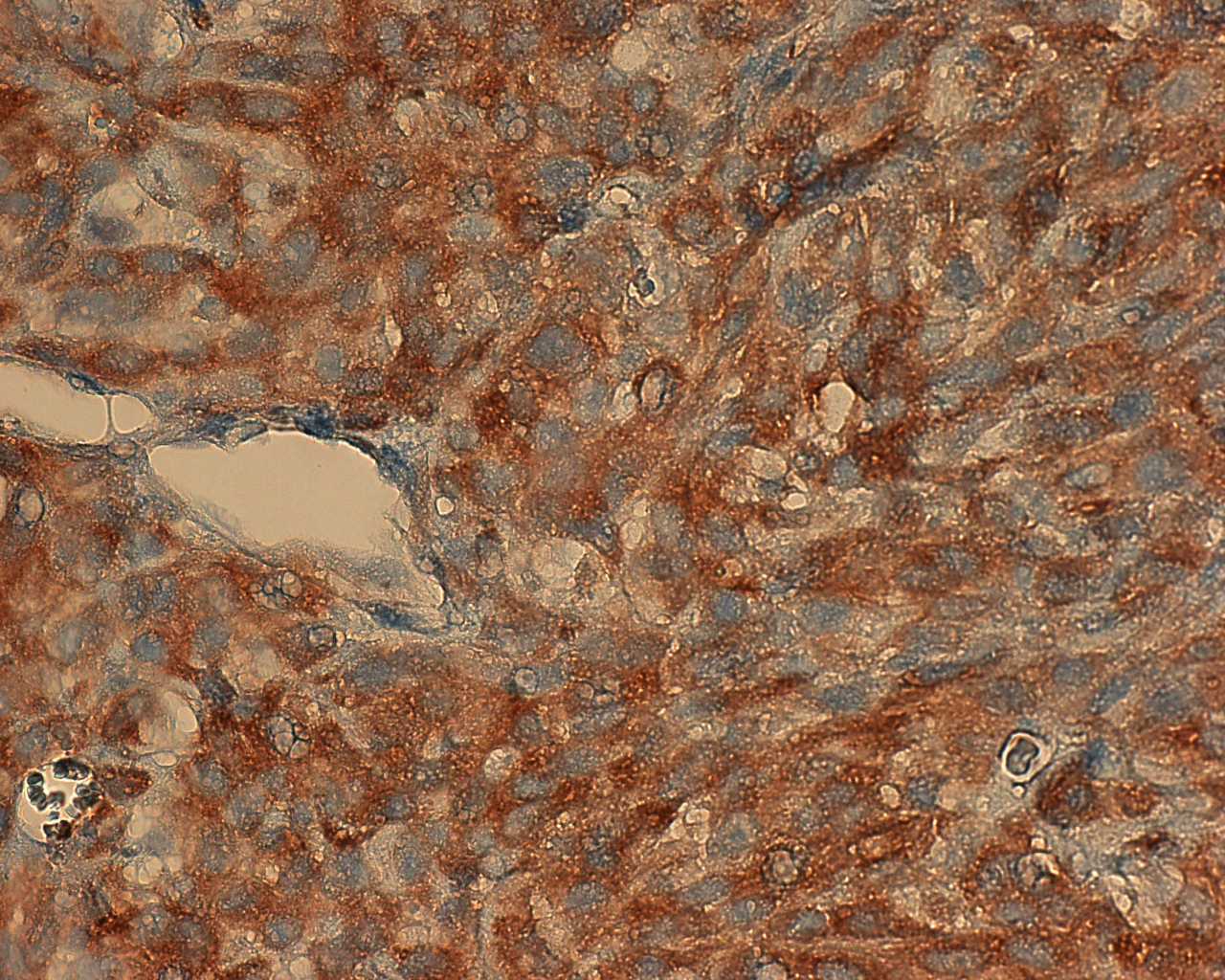

Supplement: Supplementary file 18 — Source data Fig. 8 [file 44319_2025_425_MOESM18_ESM.zip › Figure 8/8E/praja2 WT.tif]

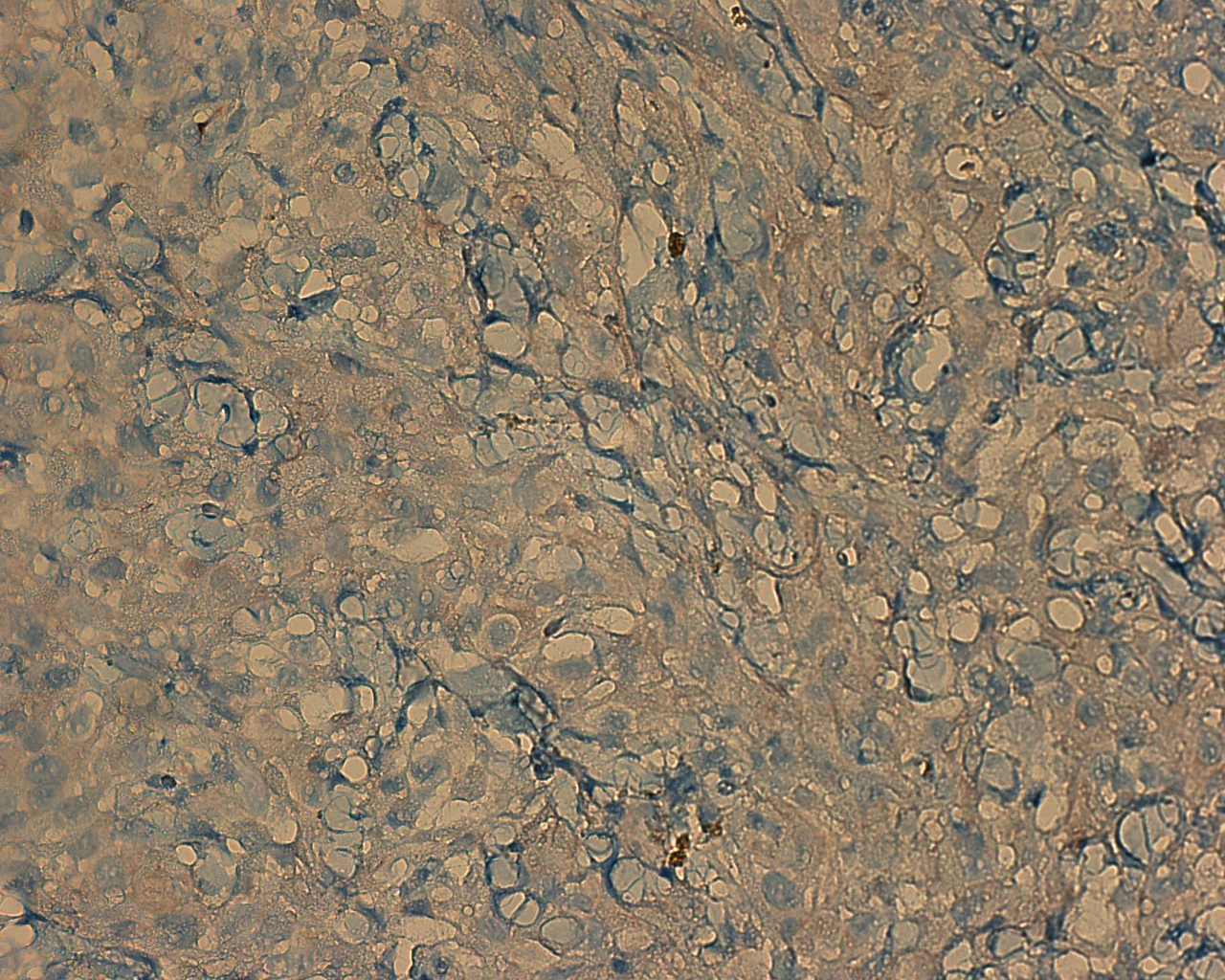

Supplement: Supplementary file 18 — Source data Fig. 8 [file 44319_2025_425_MOESM18_ESM.zip › Figure 8/8E/praja2 praja2KO.tif]

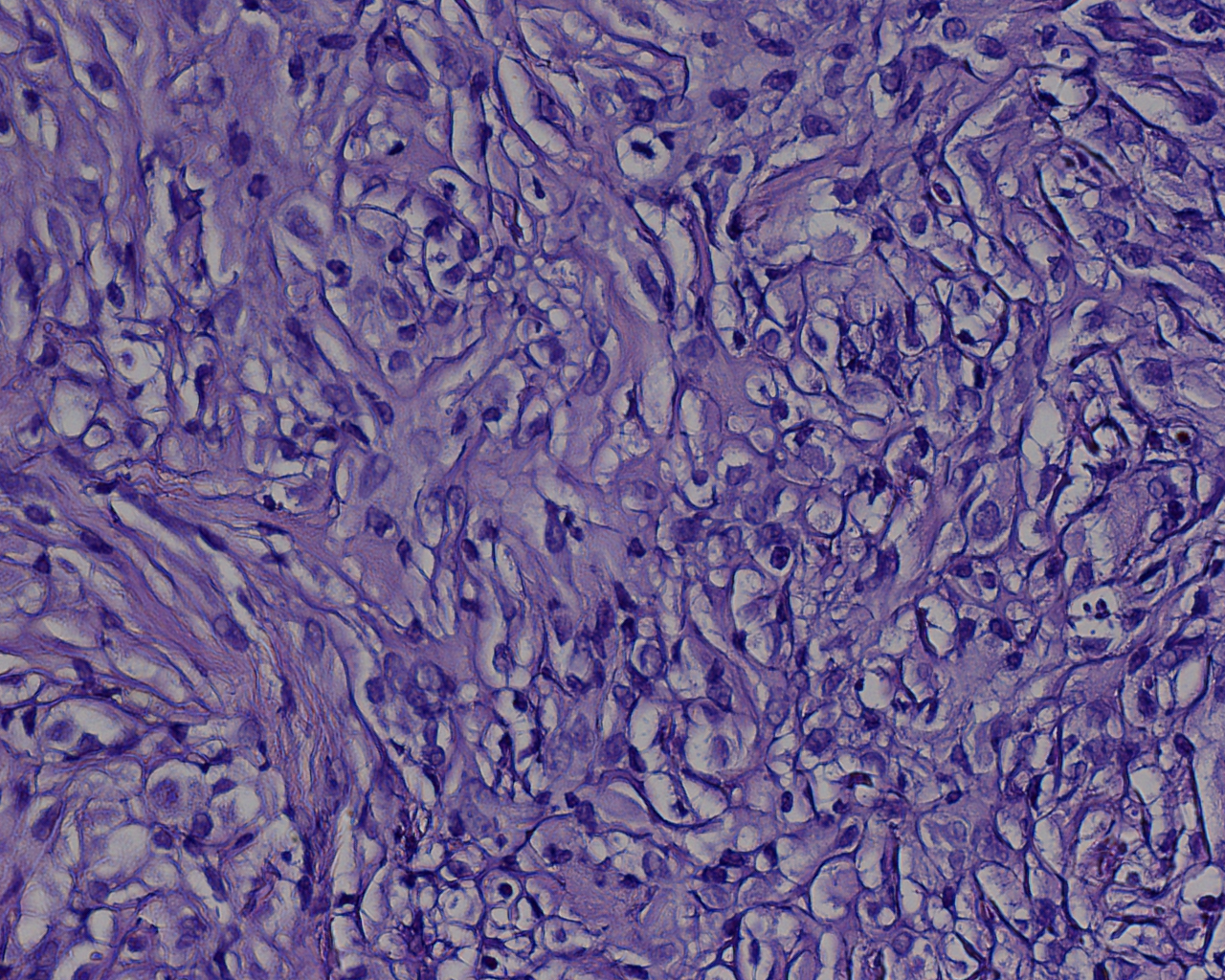

Supplement: Supplementary file 18 — Source data Fig. 8 [file 44319_2025_425_MOESM18_ESM.zip › Figure 8/8E/H:E praja2KO.tif]

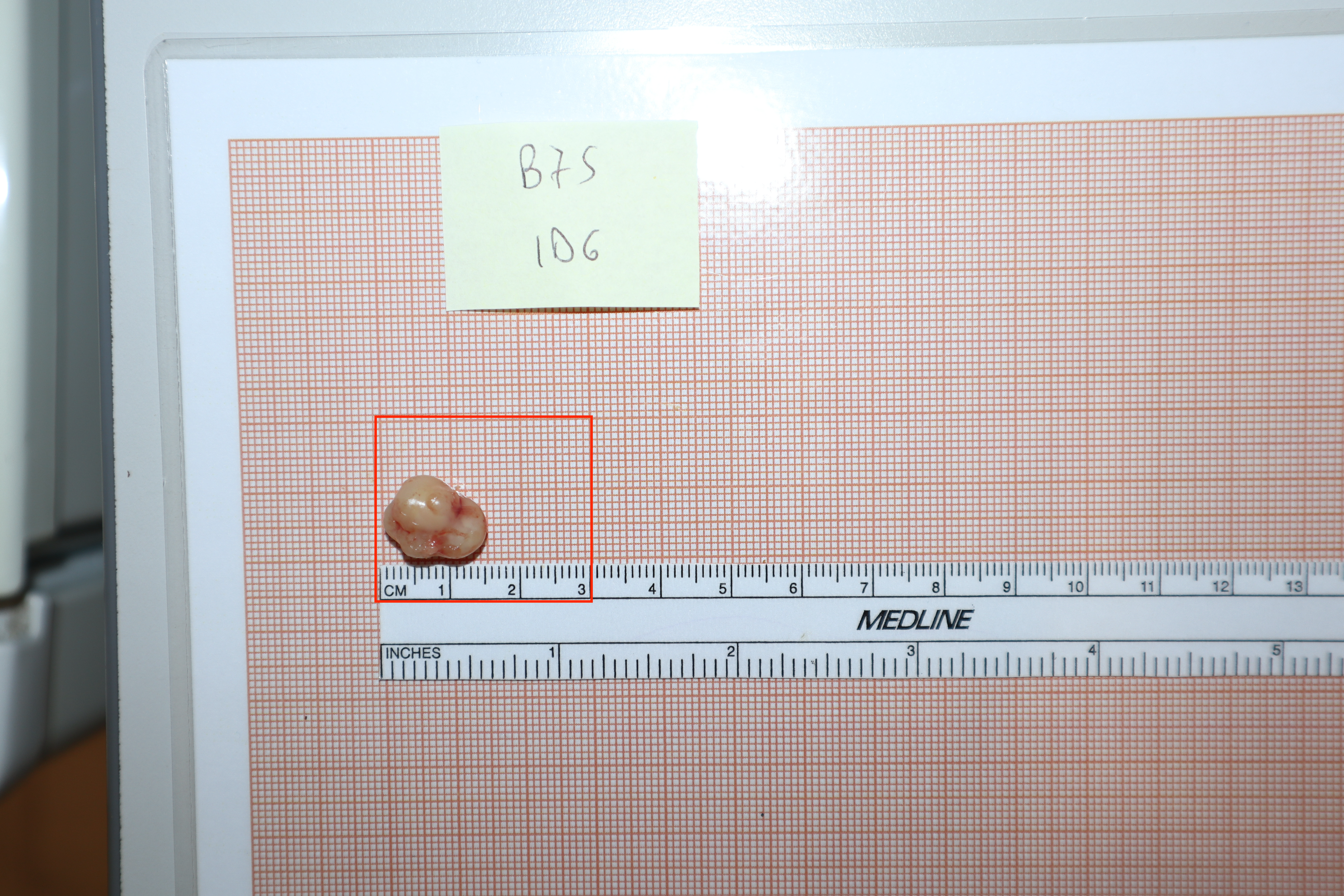

Supplement: Supplementary file 19 — Source data Fig. 9 [file 44319_2025_425_MOESM19_ESM.zip › Figure 9/9F/U87_luc2 empty right panel.png]

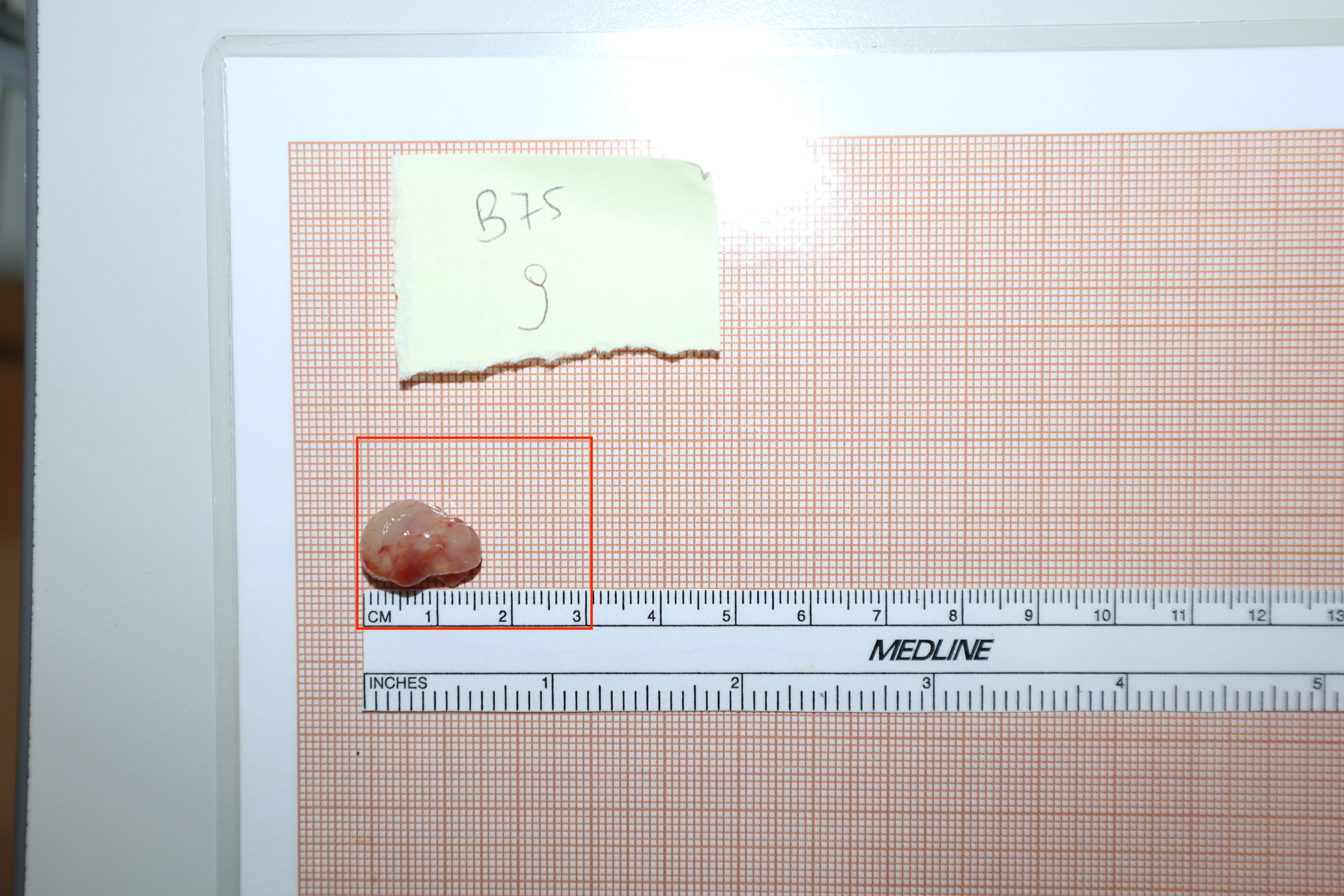

Supplement: Supplementary file 19 — Source data Fig. 9 [file 44319_2025_425_MOESM19_ESM.zip › Figure 9/9F/U87_luc2 DDX6-myc left panel.png]

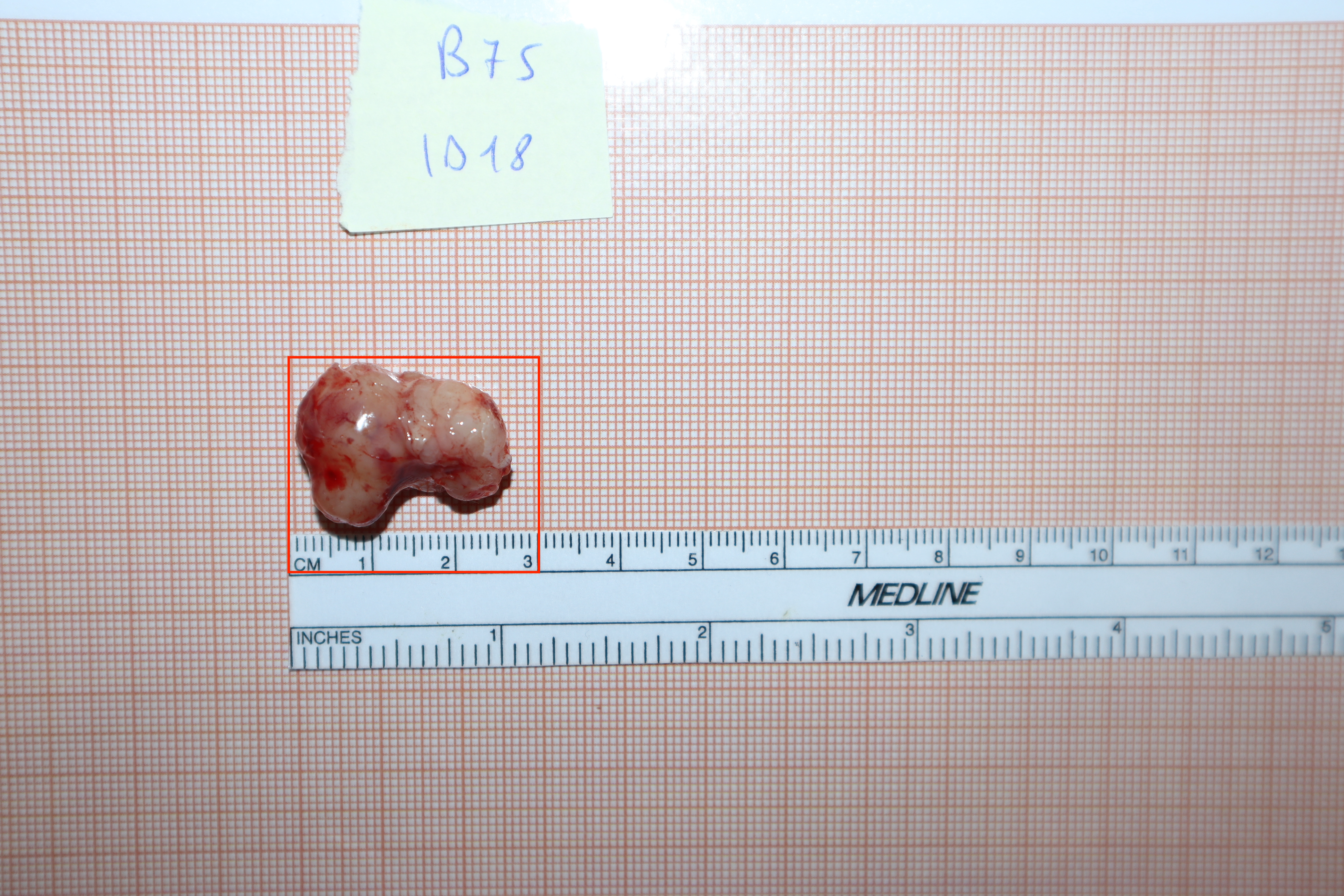

Supplement: Supplementary file 19 — Source data Fig. 9 [file 44319_2025_425_MOESM19_ESM.zip › Figure 9/9F/U87_luc2 allK:R-myc right panel.png]

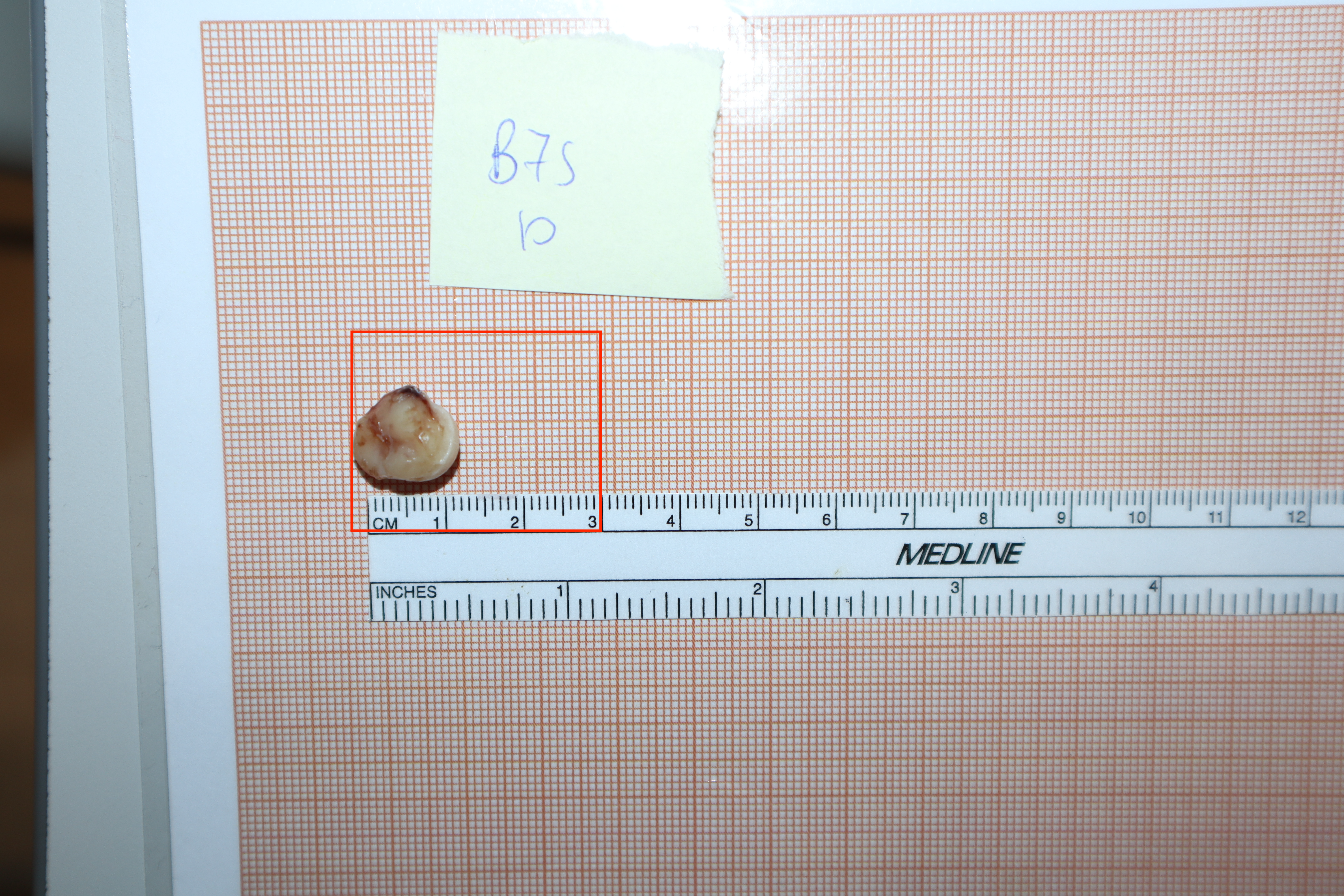

Supplement: Supplementary file 19 — Source data Fig. 9 [file 44319_2025_425_MOESM19_ESM.zip › Figure 9/9F/U87_luc2 DDX6-myc right panel.png]

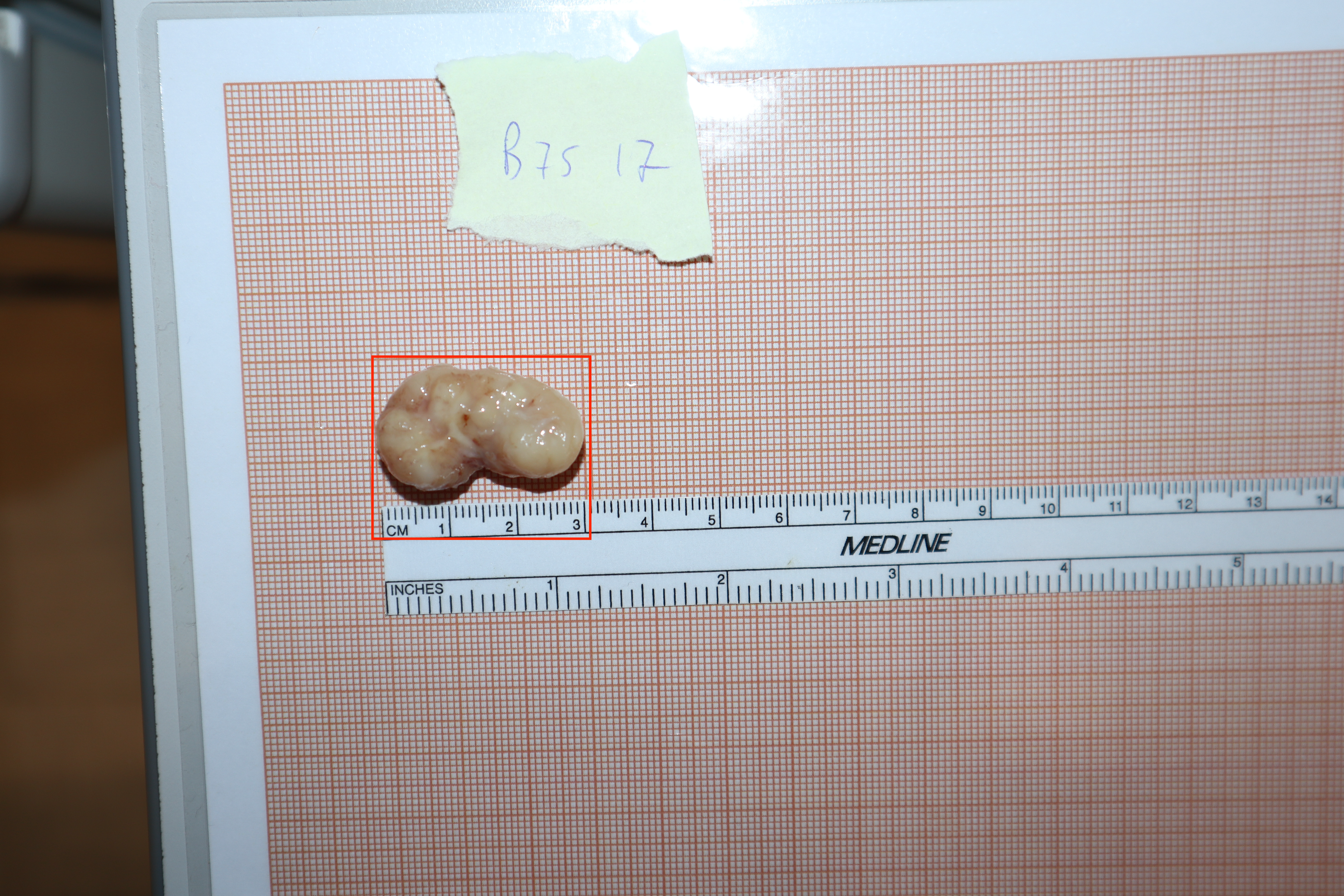

Supplement: Supplementary file 19 — Source data Fig. 9 [file 44319_2025_425_MOESM19_ESM.zip › Figure 9/9F/U87_luc2 allK:R-myc left panel.png]

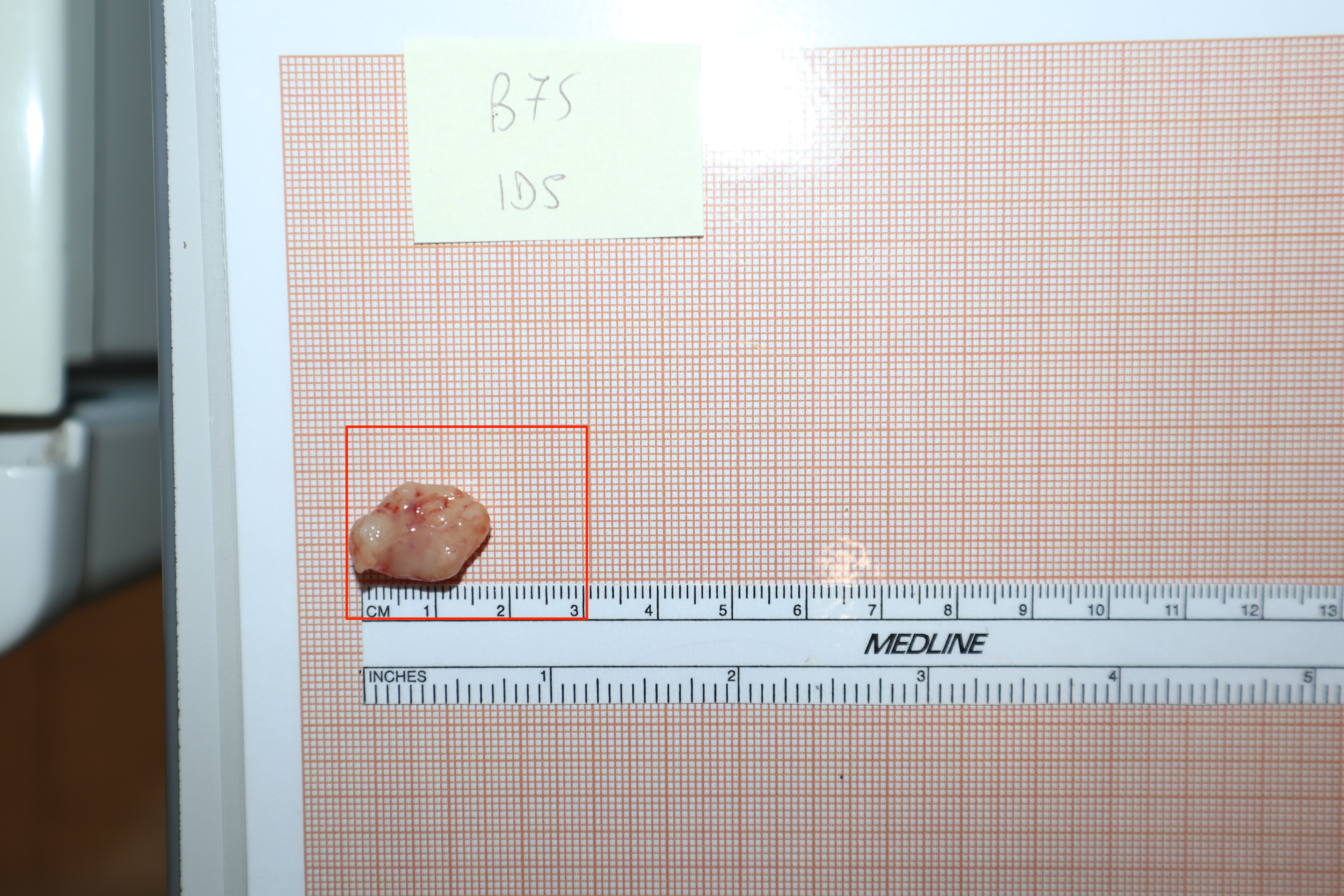

Supplement: Supplementary file 19 — Source data Fig. 9 [file 44319_2025_425_MOESM19_ESM.zip › Figure 9/9F/U87_luc2 empty left panel.png]

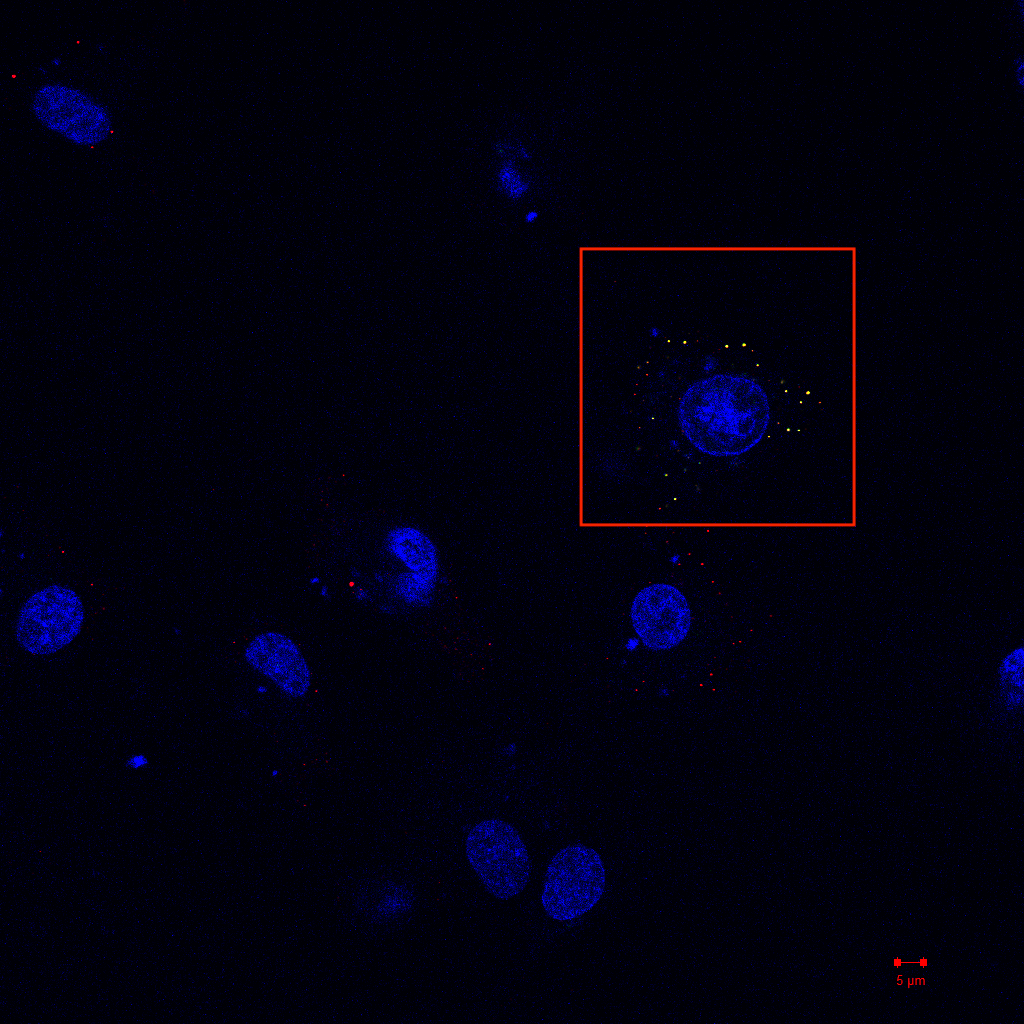

Supplement: Supplementary file 19 — Source data Fig. 9 [file 44319_2025_425_MOESM19_ESM.zip › Figure 9/9A/DDX6-myc -.tiff]

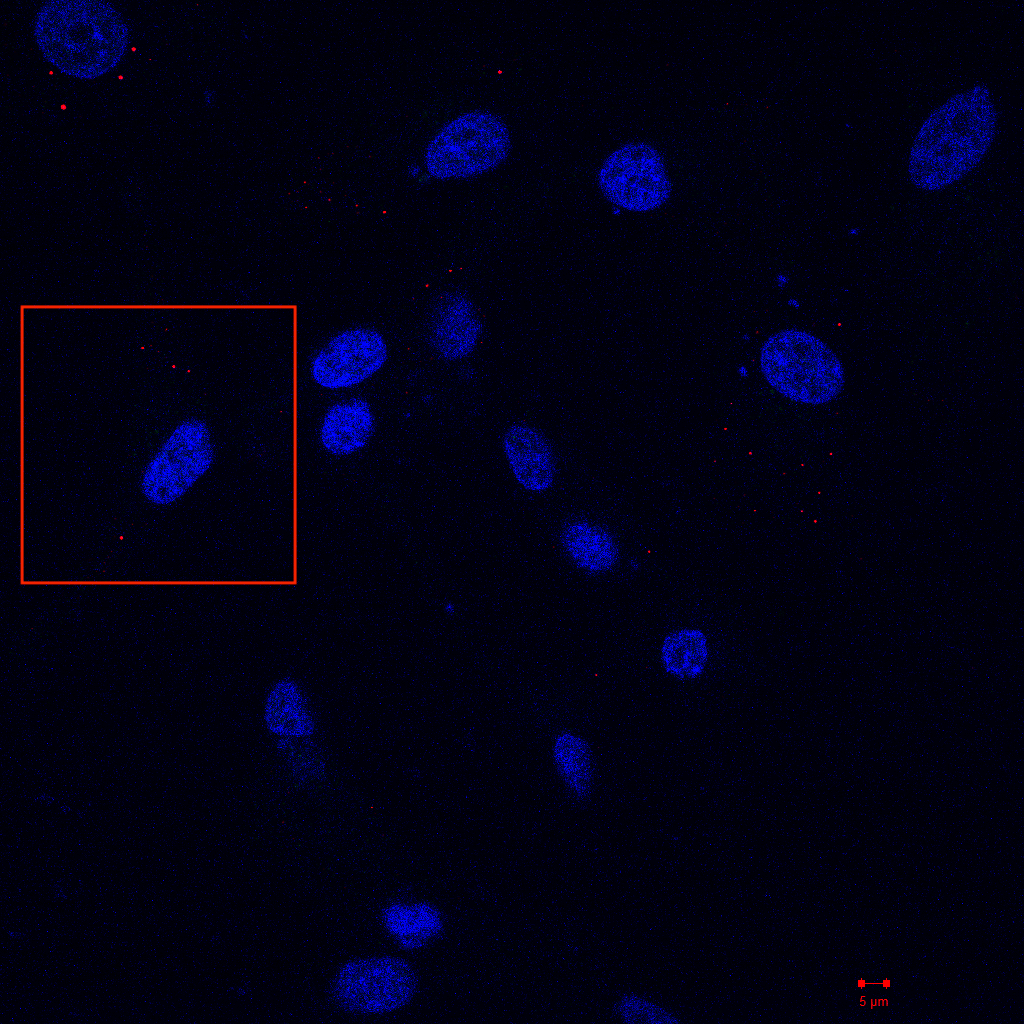

Supplement: Supplementary file 19 — Source data Fig. 9 [file 44319_2025_425_MOESM19_ESM.zip › Figure 9/9A/NT -.tiff]

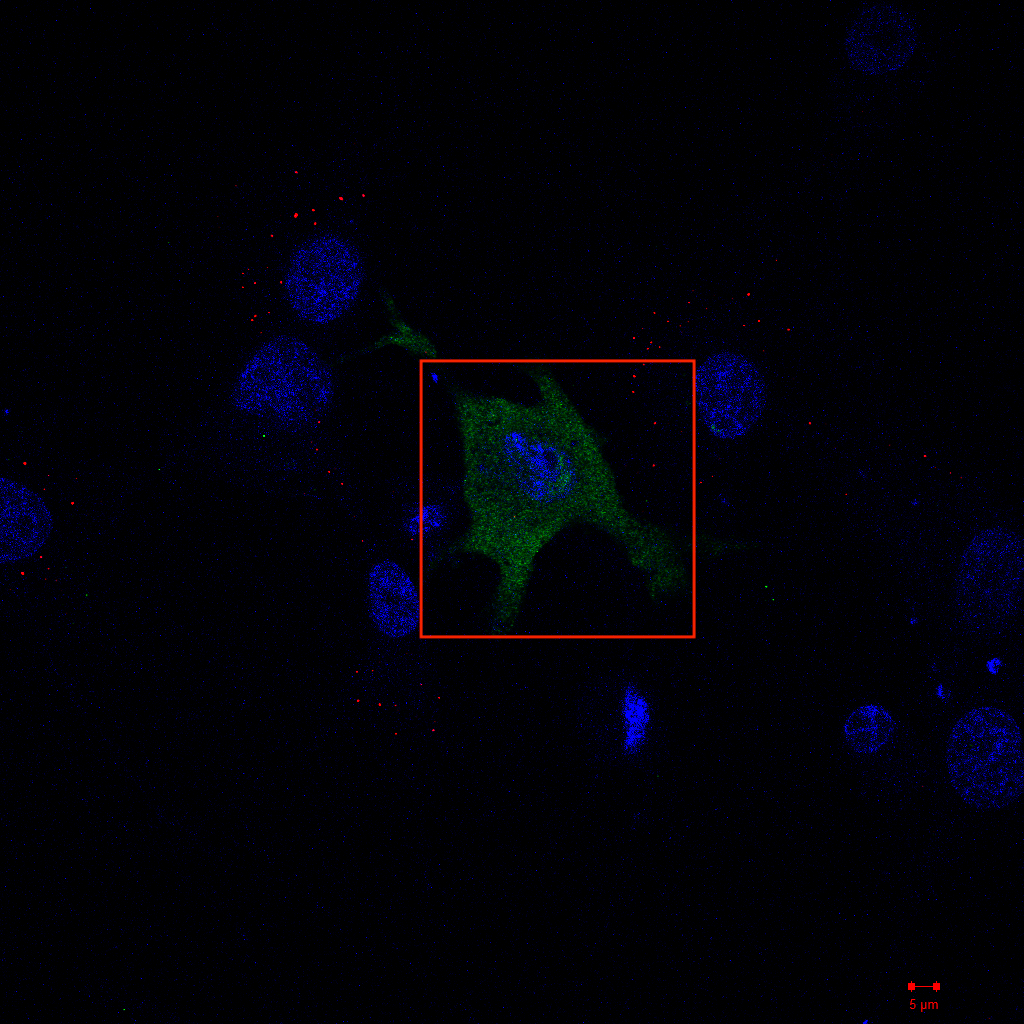

Supplement: Supplementary file 19 — Source data Fig. 9 [file 44319_2025_425_MOESM19_ESM.zip › Figure 9/9A/allK:R-myc 60' Fsk.tiff]

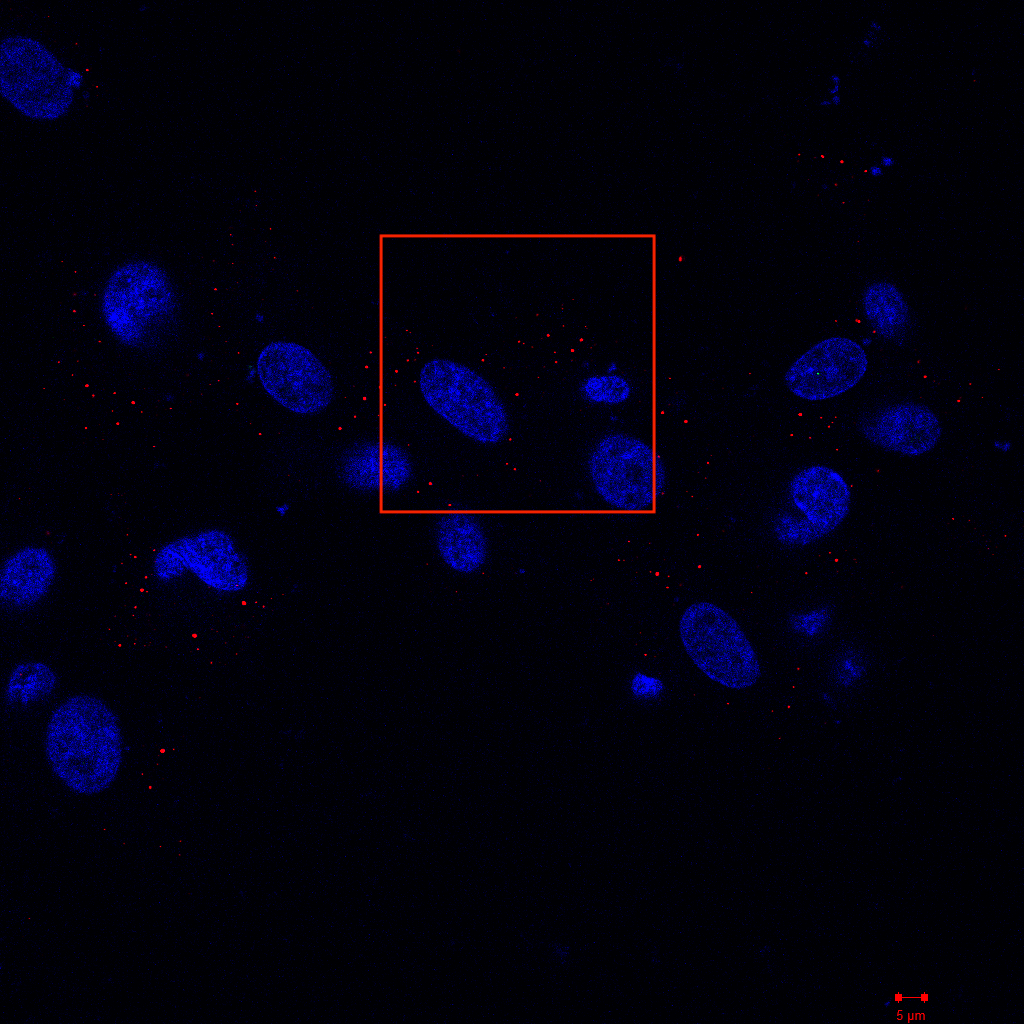

Supplement: Supplementary file 19 — Source data Fig. 9 [file 44319_2025_425_MOESM19_ESM.zip › Figure 9/9A/NT 60' Fsk.tiff]

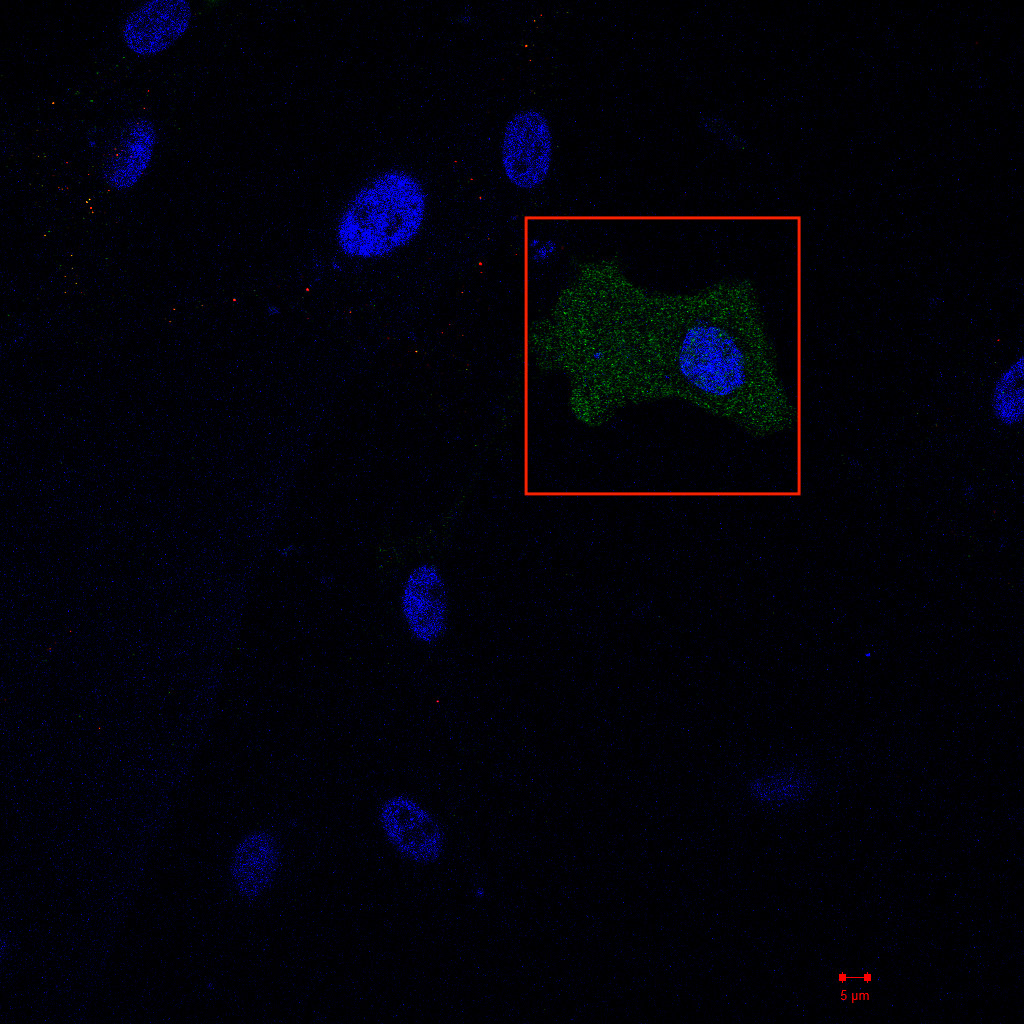

Supplement: Supplementary file 19 — Source data Fig. 9 [file 44319_2025_425_MOESM19_ESM.zip › Figure 9/9A/allK:R-myc -.tiff]

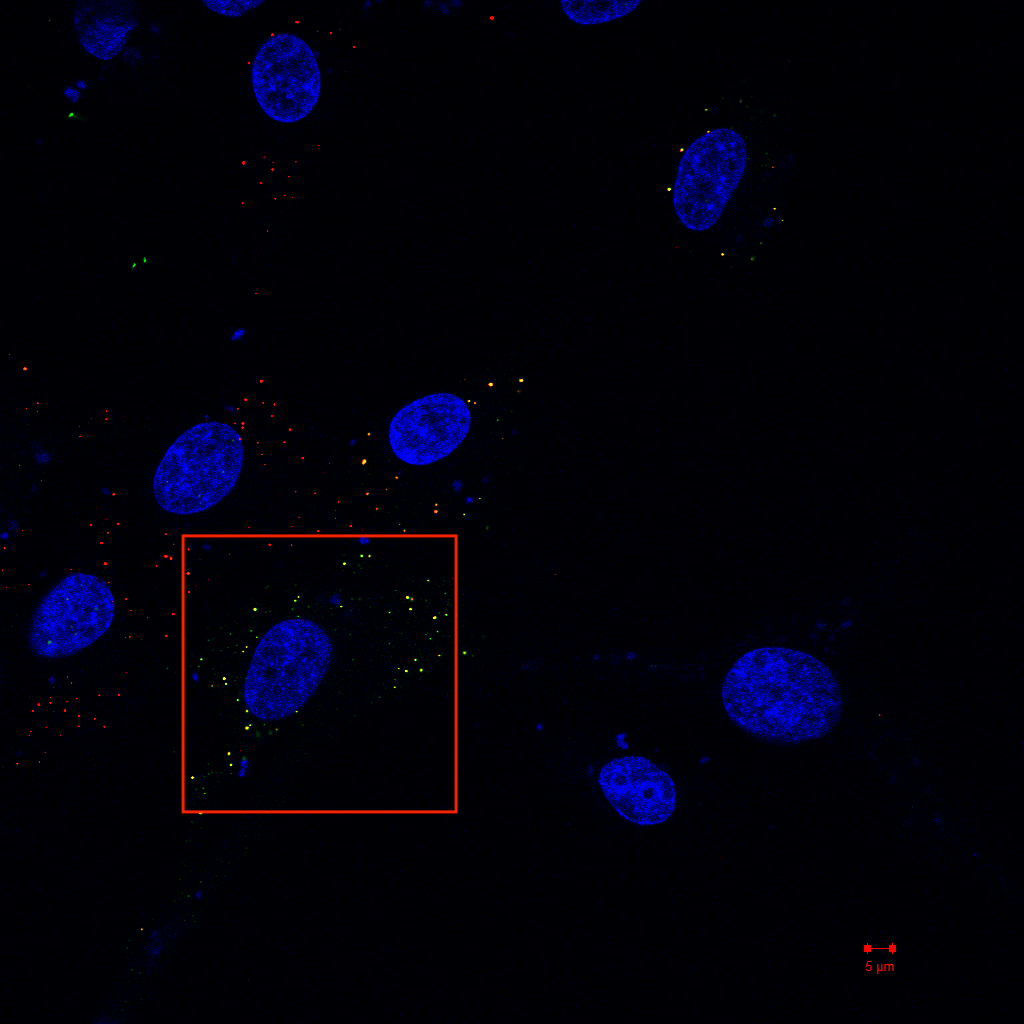

Supplement: Supplementary file 19 — Source data Fig. 9 [file 44319_2025_425_MOESM19_ESM.zip › Figure 9/9A/DDX6-myc 60' Fsk.tiff]

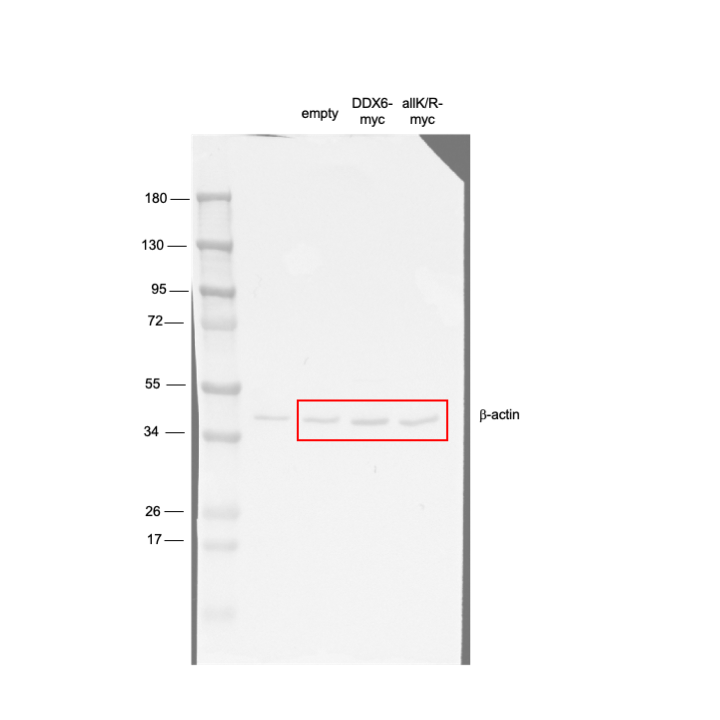

Supplement: Supplementary file 19 — Source data Fig. 9 [file 44319_2025_425_MOESM19_ESM.zip › Figure 9/9G/b-actin.tiff]

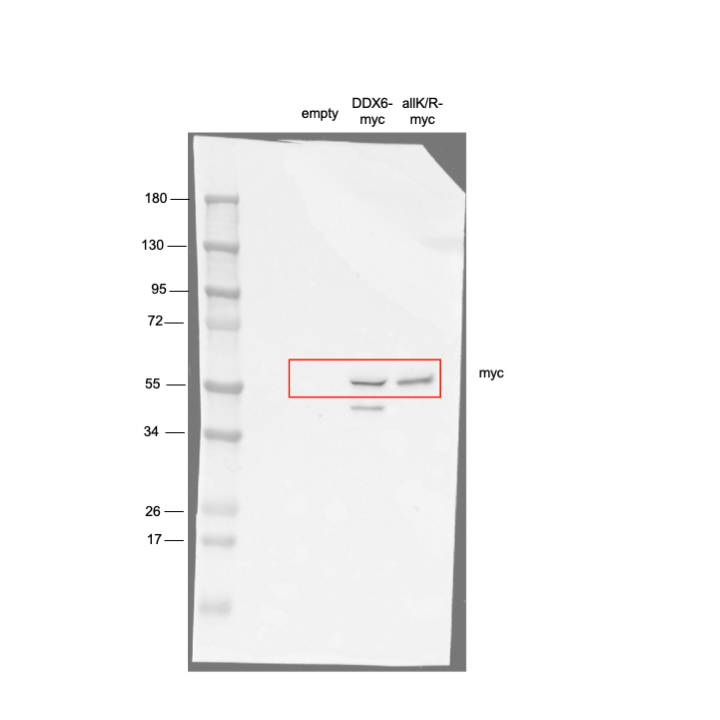

Supplement: Supplementary file 19 — Source data Fig. 9 [file 44319_2025_425_MOESM19_ESM.zip › Figure 9/9G/myc.tiff]
