# Supplementary figures and images for: Praja2 controls P-body assembly and translation in glioblastoma by non-proteolytic ubiquitylation of DDX6 (part 2 of 2)
Source: EMBO Rep. 2025 Mar 27;26(9):2347–77. doi: 10.1038/s44319-025-00425-5 (PMC12069581; doi:10.1038/s44319-025-00425-5)

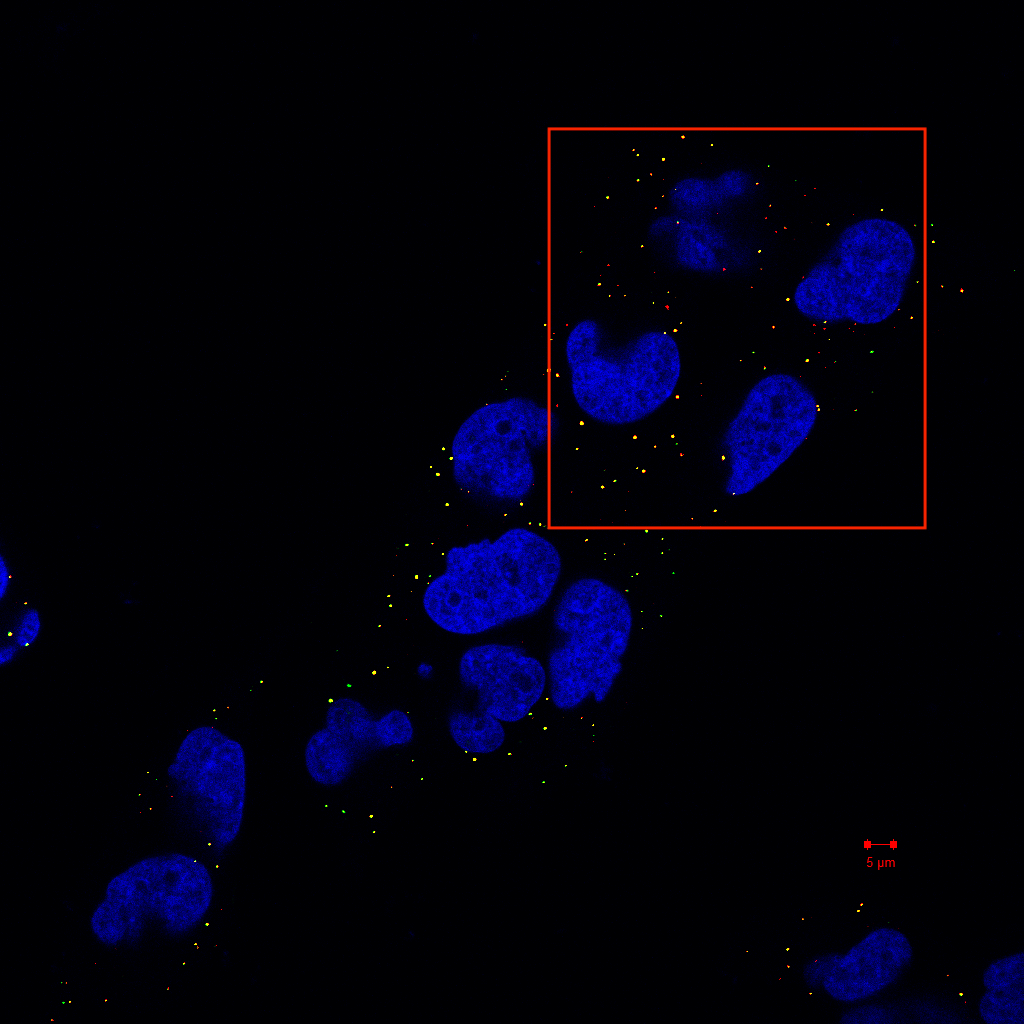

Supplement: Supplementary file 20 — Figure Source Data for EV [file 44319_2025_425_MOESM20_ESM.zip › EV /EV4A/60' Fsk.tif]

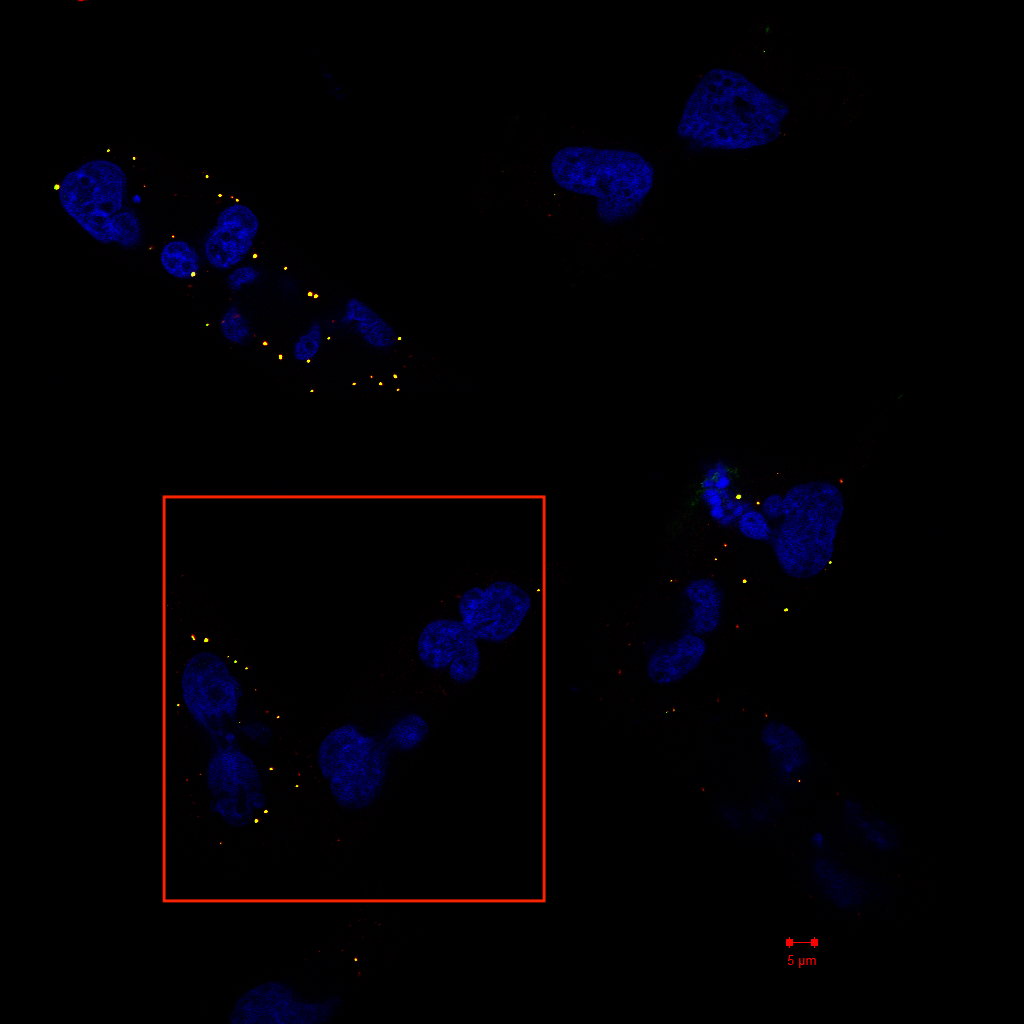

Supplement: Supplementary file 20 — Figure Source Data for EV [file 44319_2025_425_MOESM20_ESM.zip › EV /EV4A/-.tif]

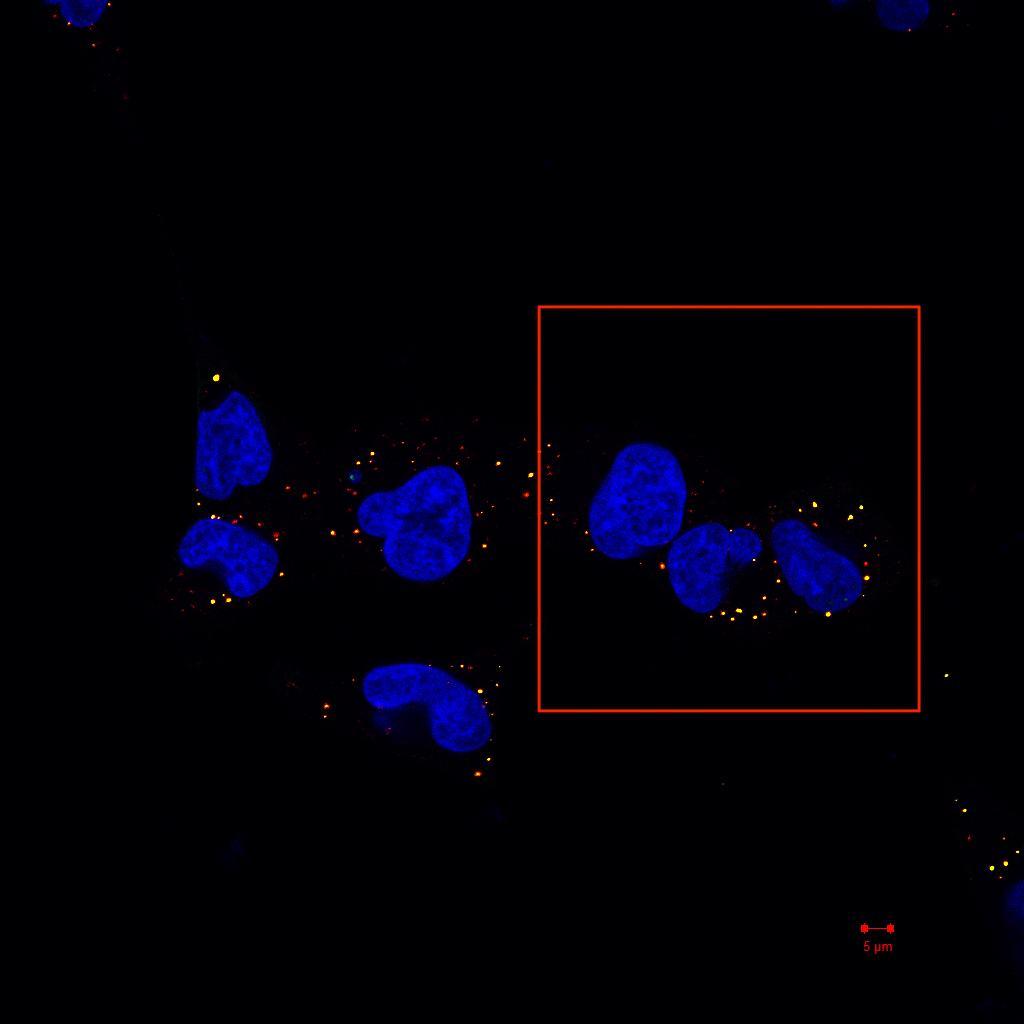

Supplement: Supplementary file 20 — Figure Source Data for EV [file 44319_2025_425_MOESM20_ESM.zip › EV /EV4A/30' Fsk.tif]

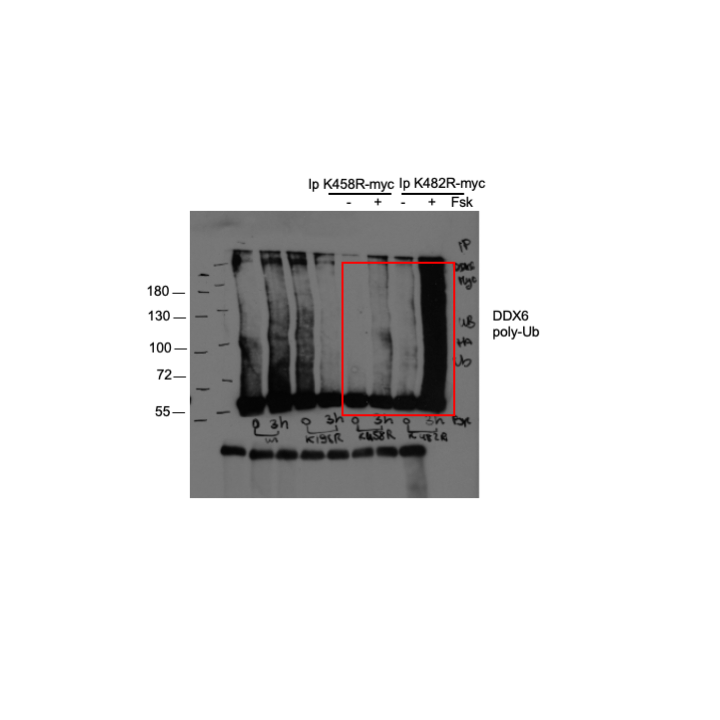

Supplement: Supplementary file 20 — Figure Source Data for EV [file 44319_2025_425_MOESM20_ESM.zip › EV /AppendixS1/S1F/DDX6 poly-Ub.tiff]

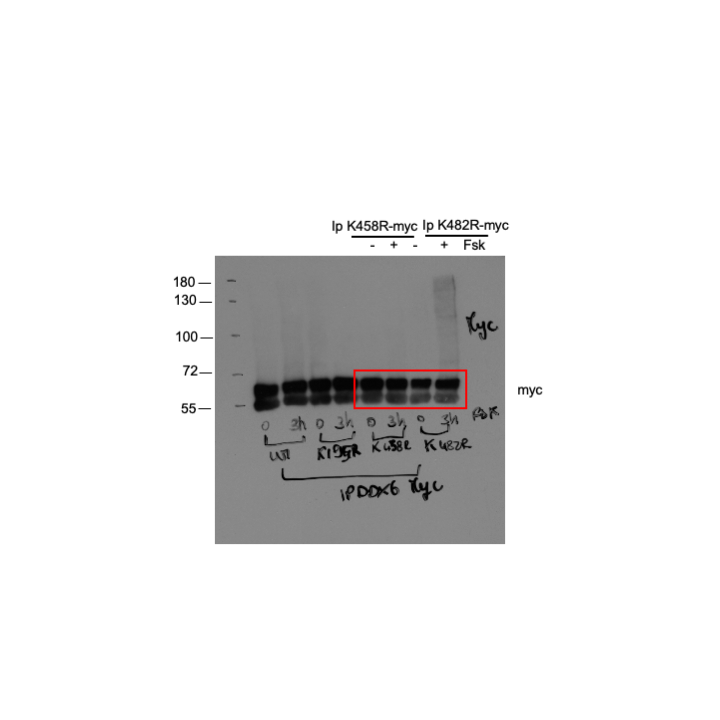

Supplement: Supplementary file 20 — Figure Source Data for EV [file 44319_2025_425_MOESM20_ESM.zip › EV /AppendixS1/S1F/myc.tiff]

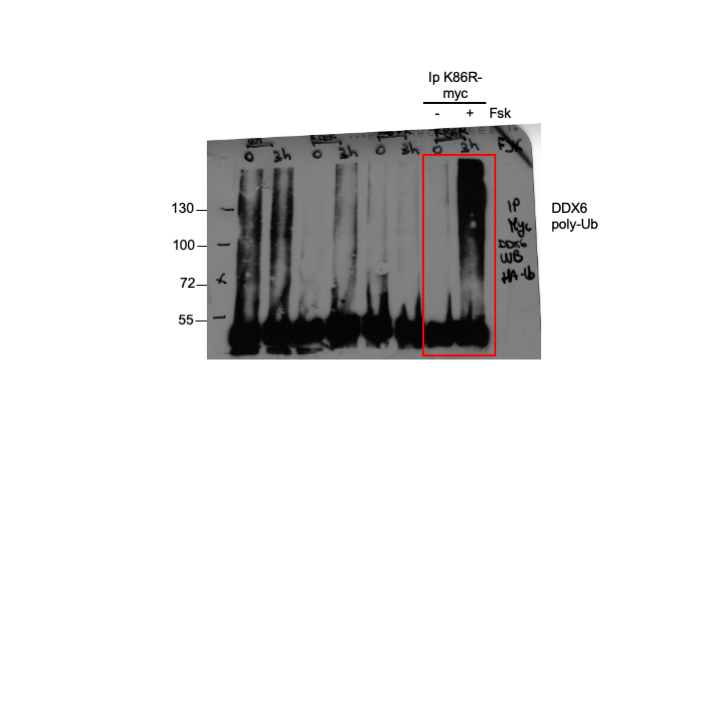

Supplement: Supplementary file 20 — Figure Source Data for EV [file 44319_2025_425_MOESM20_ESM.zip › EV /AppendixS1/S1A/DDX6 poly-Ub.tiff]

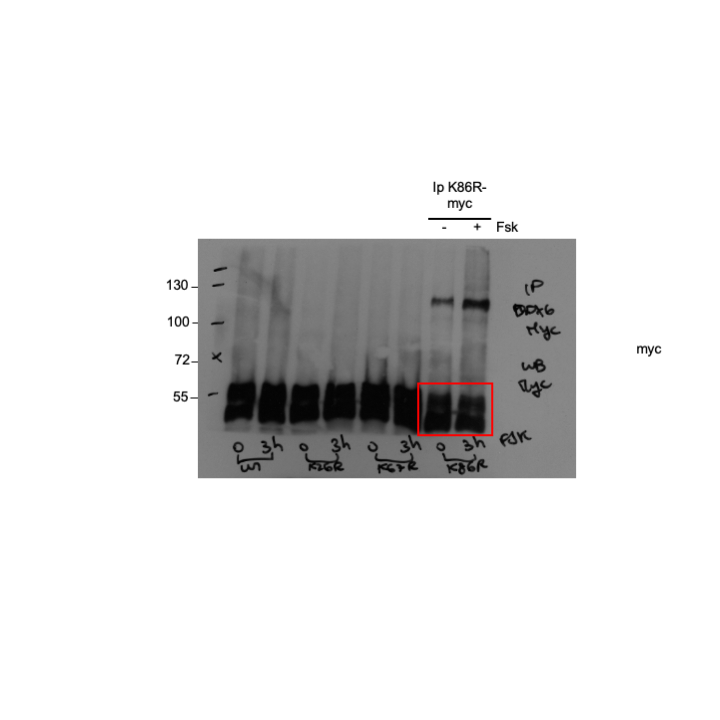

Supplement: Supplementary file 20 — Figure Source Data for EV [file 44319_2025_425_MOESM20_ESM.zip › EV /AppendixS1/S1A/myc.tiff]

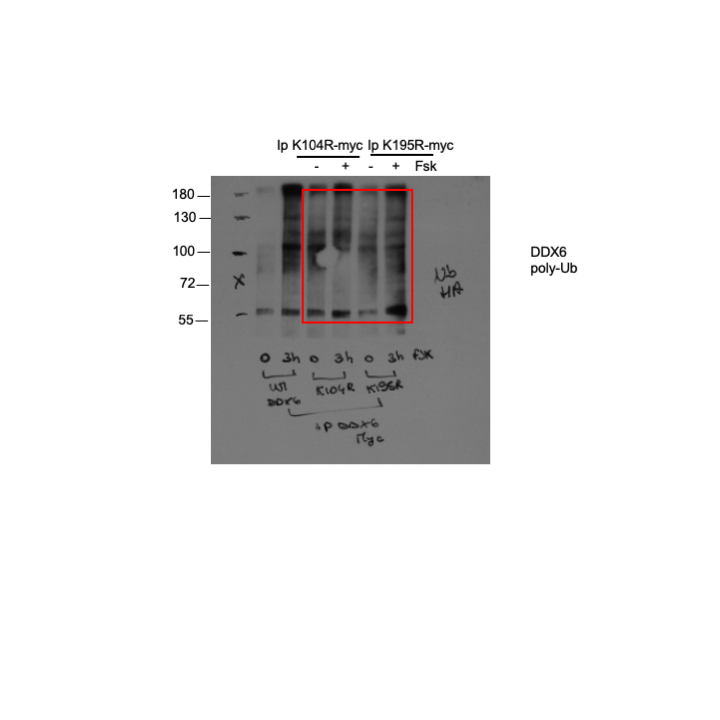

Supplement: Supplementary file 20 — Figure Source Data for EV [file 44319_2025_425_MOESM20_ESM.zip › EV /AppendixS1/S1D/DDX6 poly-Ub.tiff]

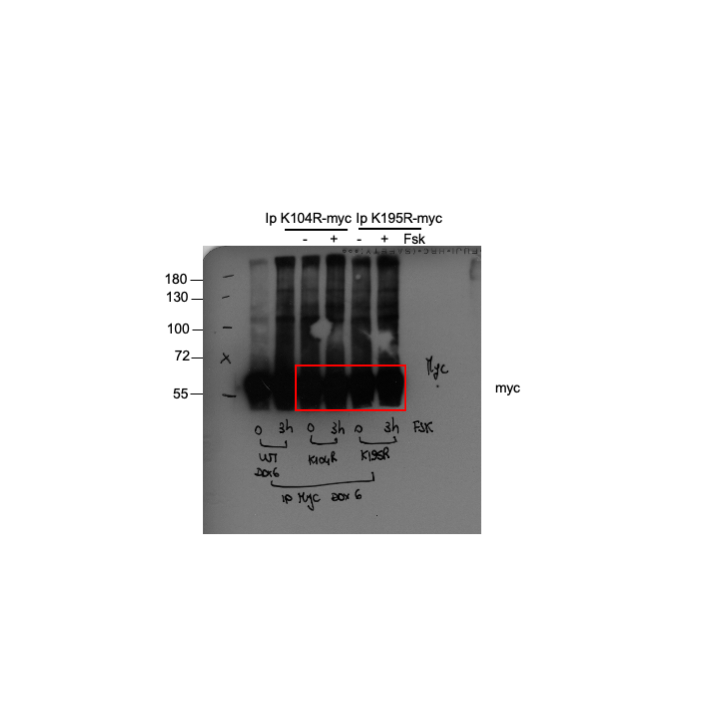

Supplement: Supplementary file 20 — Figure Source Data for EV [file 44319_2025_425_MOESM20_ESM.zip › EV /AppendixS1/S1D/myc.tiff]

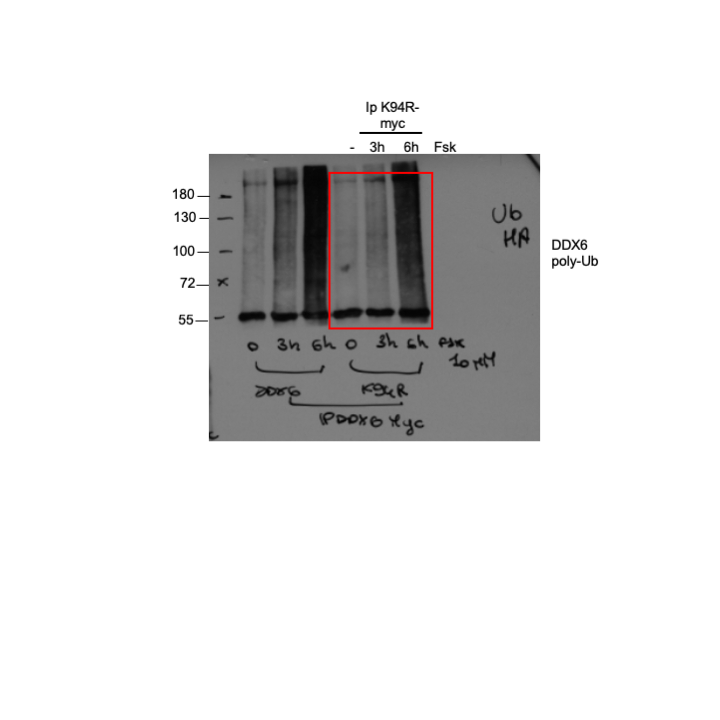

Supplement: Supplementary file 20 — Figure Source Data for EV [file 44319_2025_425_MOESM20_ESM.zip › EV /AppendixS1/S1C/DDX6 poly-Ub.tiff]

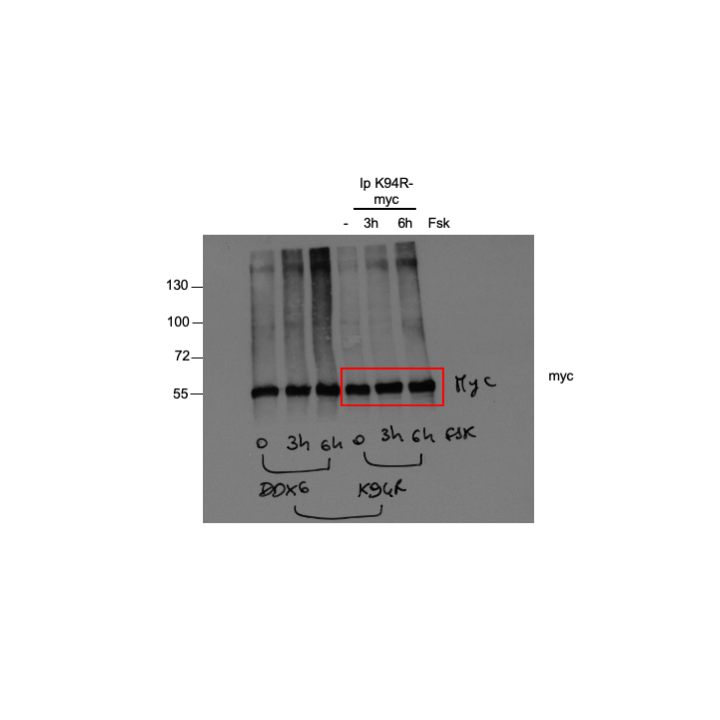

Supplement: Supplementary file 20 — Figure Source Data for EV [file 44319_2025_425_MOESM20_ESM.zip › EV /AppendixS1/S1C/myc.tiff]

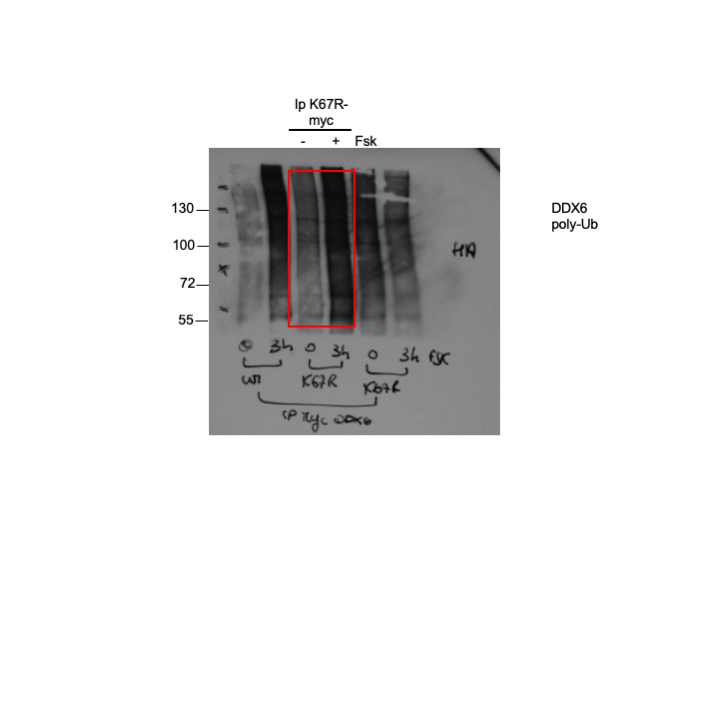

Supplement: Supplementary file 20 — Figure Source Data for EV [file 44319_2025_425_MOESM20_ESM.zip › EV /AppendixS1/S1B/DDX6 poly-Ub.tiff]

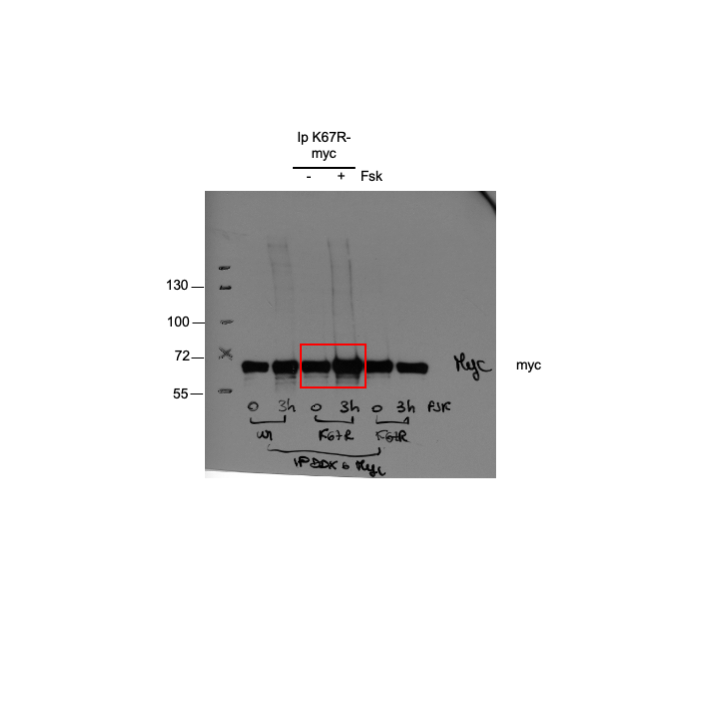

Supplement: Supplementary file 20 — Figure Source Data for EV [file 44319_2025_425_MOESM20_ESM.zip › EV /AppendixS1/S1B/myc.tiff]

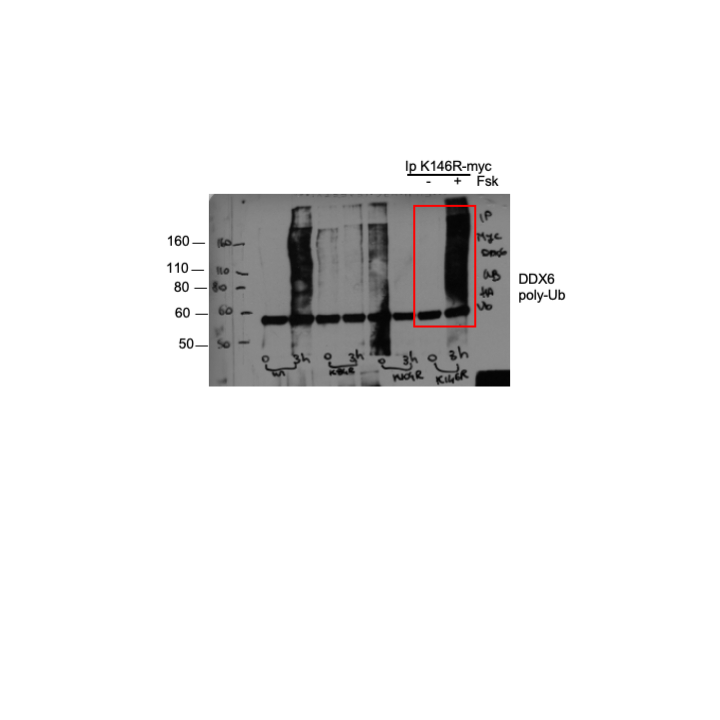

Supplement: Supplementary file 20 — Figure Source Data for EV [file 44319_2025_425_MOESM20_ESM.zip › EV /AppendixS1/S1E/DDX6 poly-Ub.tiff]

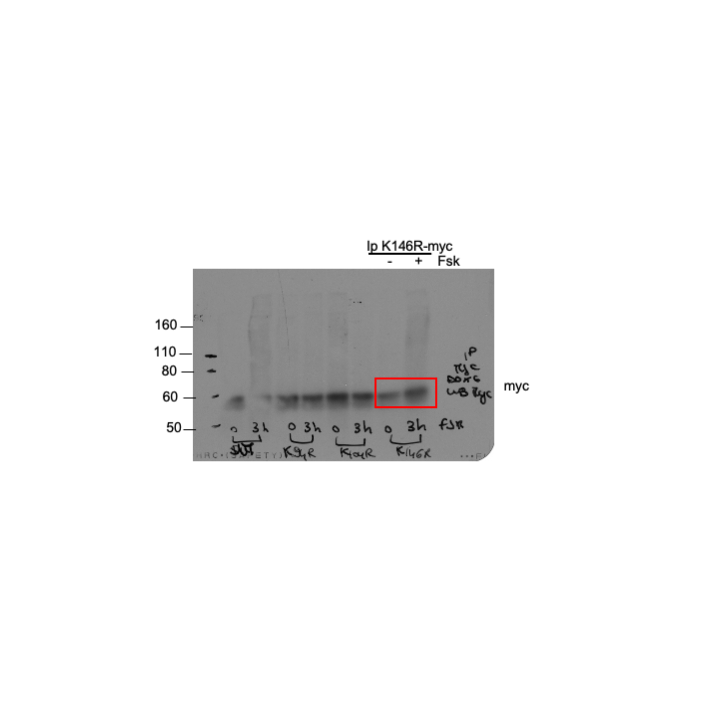

Supplement: Supplementary file 20 — Figure Source Data for EV [file 44319_2025_425_MOESM20_ESM.zip › EV /AppendixS1/S1E/myc.tiff]

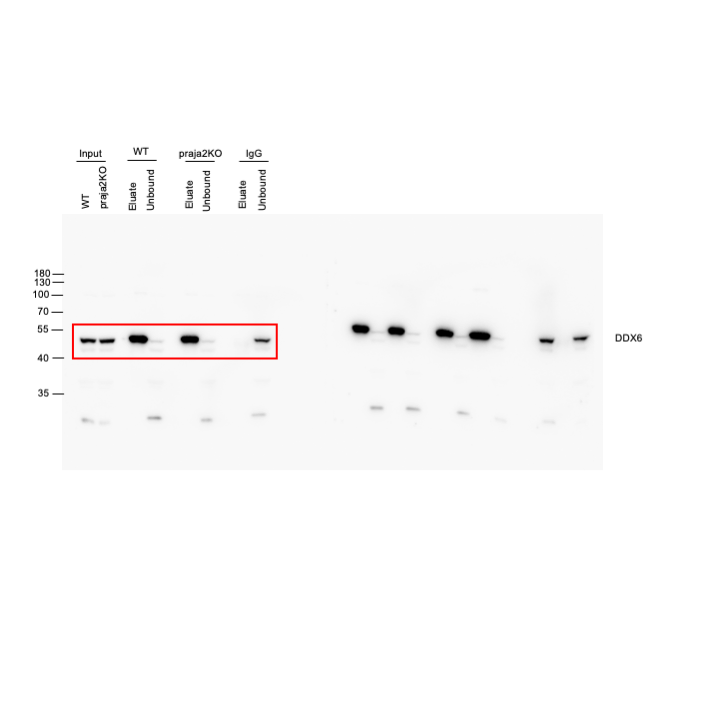

Supplement: Supplementary file 20 — Figure Source Data for EV [file 44319_2025_425_MOESM20_ESM.zip › EV /Appendix S3/S3B/DDX6.tiff]

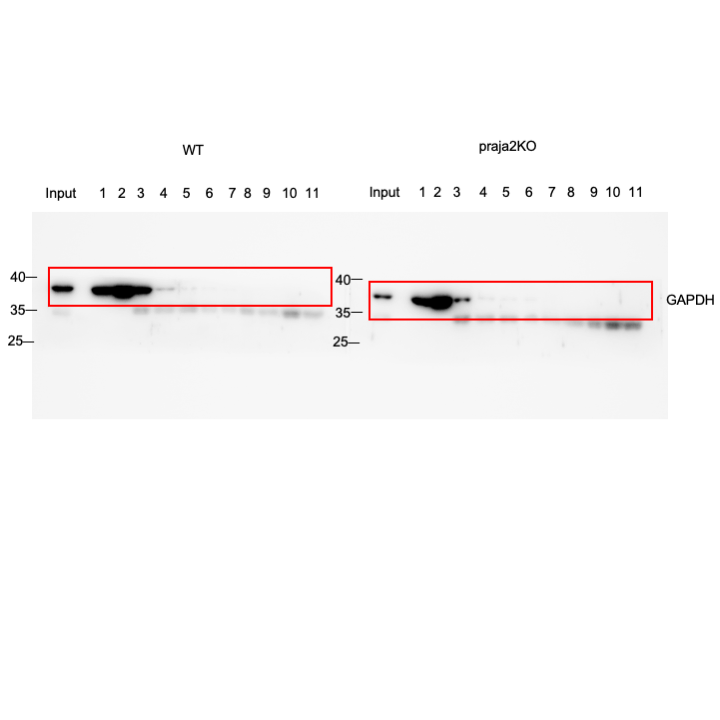

Supplement: Supplementary file 20 — Figure Source Data for EV [file 44319_2025_425_MOESM20_ESM.zip › EV /Appendix S3/S3A/GAPDH.tiff]

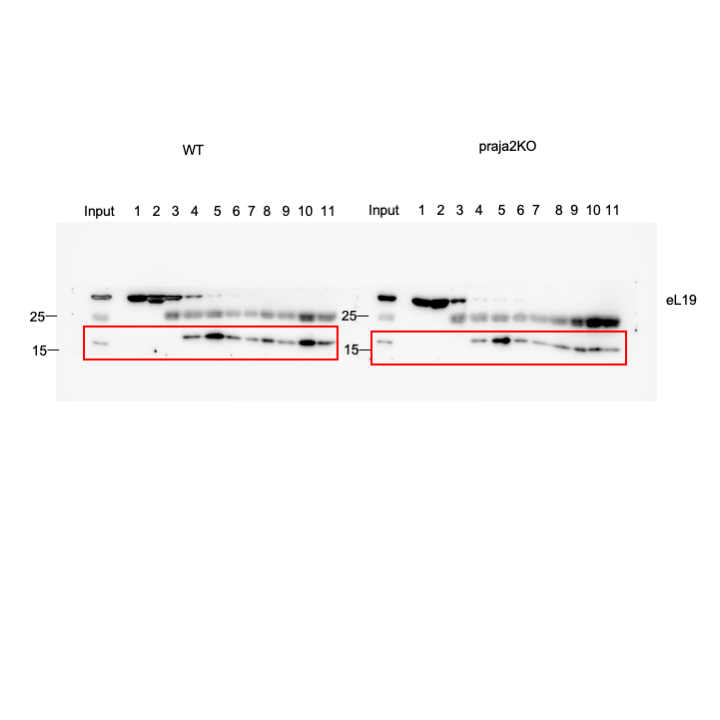

Supplement: Supplementary file 20 — Figure Source Data for EV [file 44319_2025_425_MOESM20_ESM.zip › EV /Appendix S3/S3A/eL19.tiff]

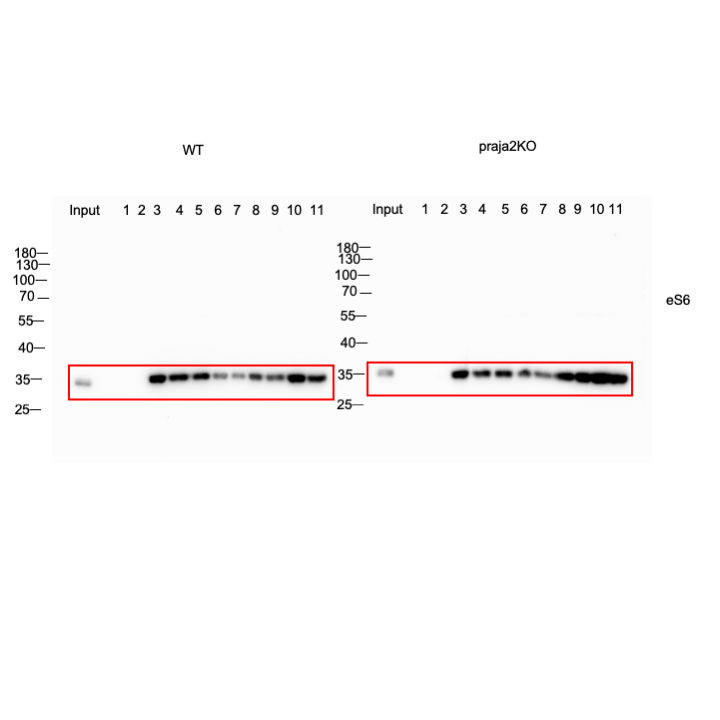

Supplement: Supplementary file 20 — Figure Source Data for EV [file 44319_2025_425_MOESM20_ESM.zip › EV /Appendix S3/S3A/eS6.tiff]

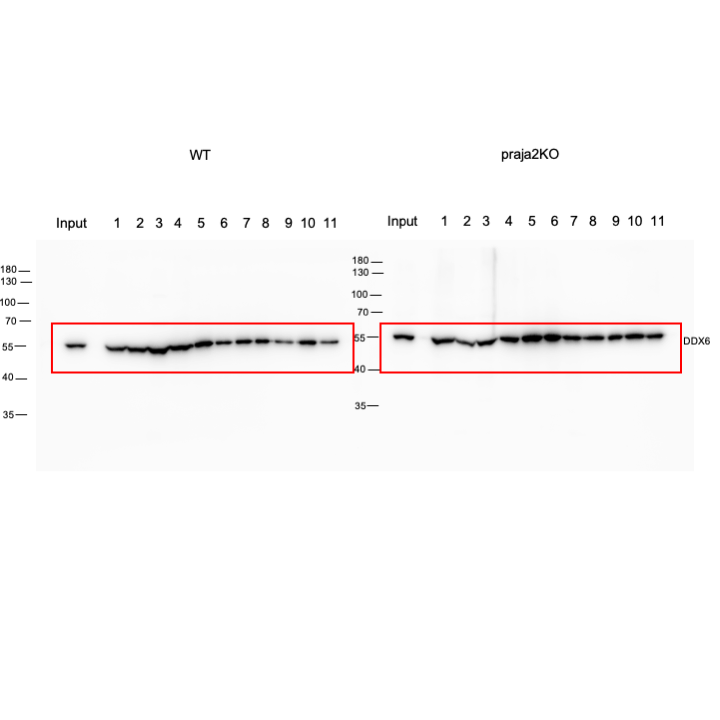

Supplement: Supplementary file 20 — Figure Source Data for EV [file 44319_2025_425_MOESM20_ESM.zip › EV /Appendix S3/S3A/DDX6.tiff]
