# Supplementary material for: Praja2 controls P-body assembly and translation in glioblastoma by non-proteolytic ubiquitylation of DDX6
Source: EMBO Rep. 2025 Mar 27;26(9):2347–77. doi: 10.1038/s44319-025-00425-5 (PMC12069581; doi:10.1038/s44319-025-00425-5)
Supplement: Supplementary file 21 — Expanded View Figures [file 44319_2025_425_MOESM21_ESM.pdf]

Expanded View Figures

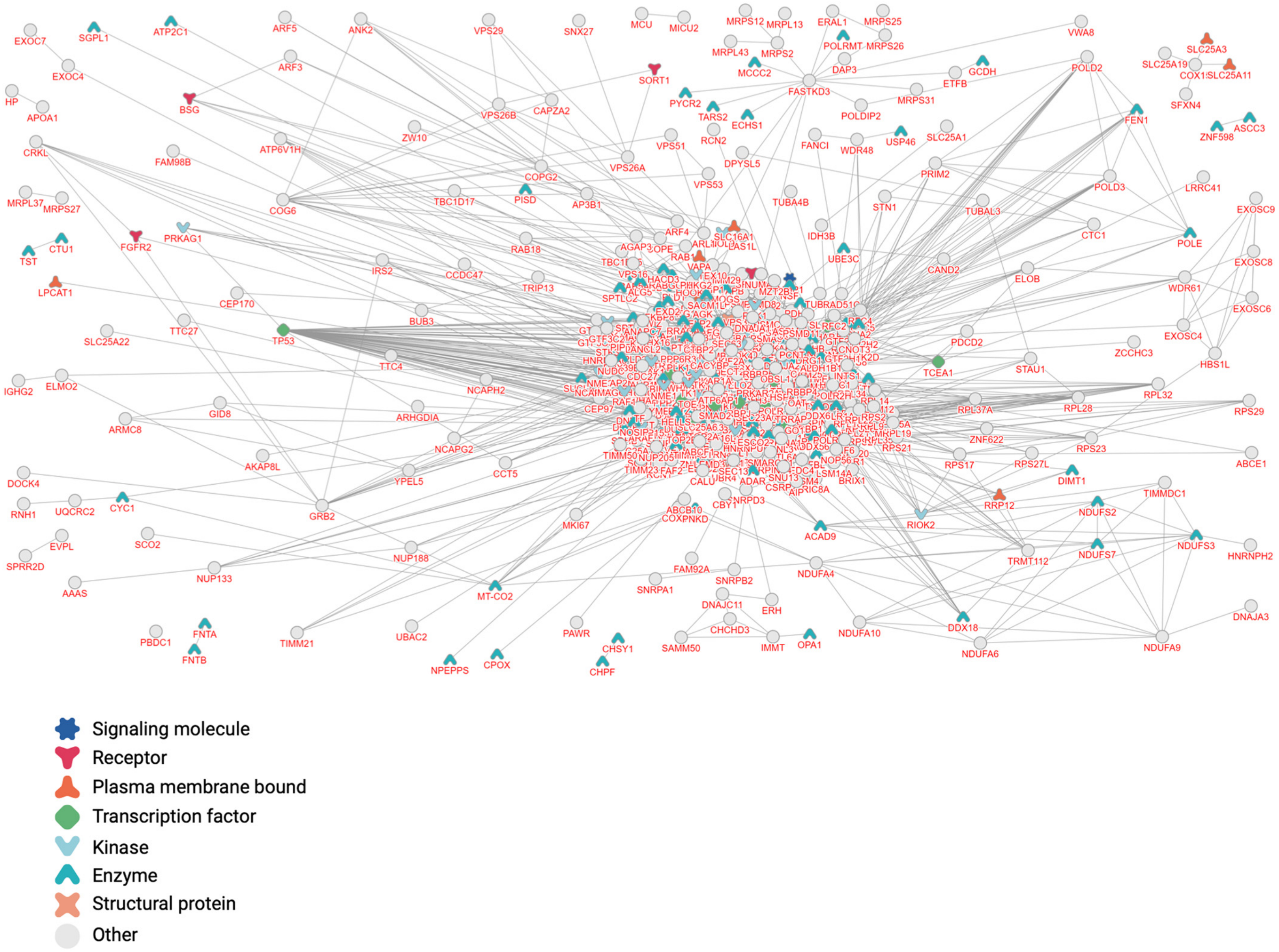

Figure EV1. Praja2 protein–protein interaction network.

Protein–protein interaction network of all the directly-interacting components belonging to the praja2 interactome, which was generated using the inBio Discover web tool (no network expansion) based on highly trusted interactions ($n = 506$ proteins, $n = 1563$ interactions). The figure legend indicates the functional roles of the displayed interactors.

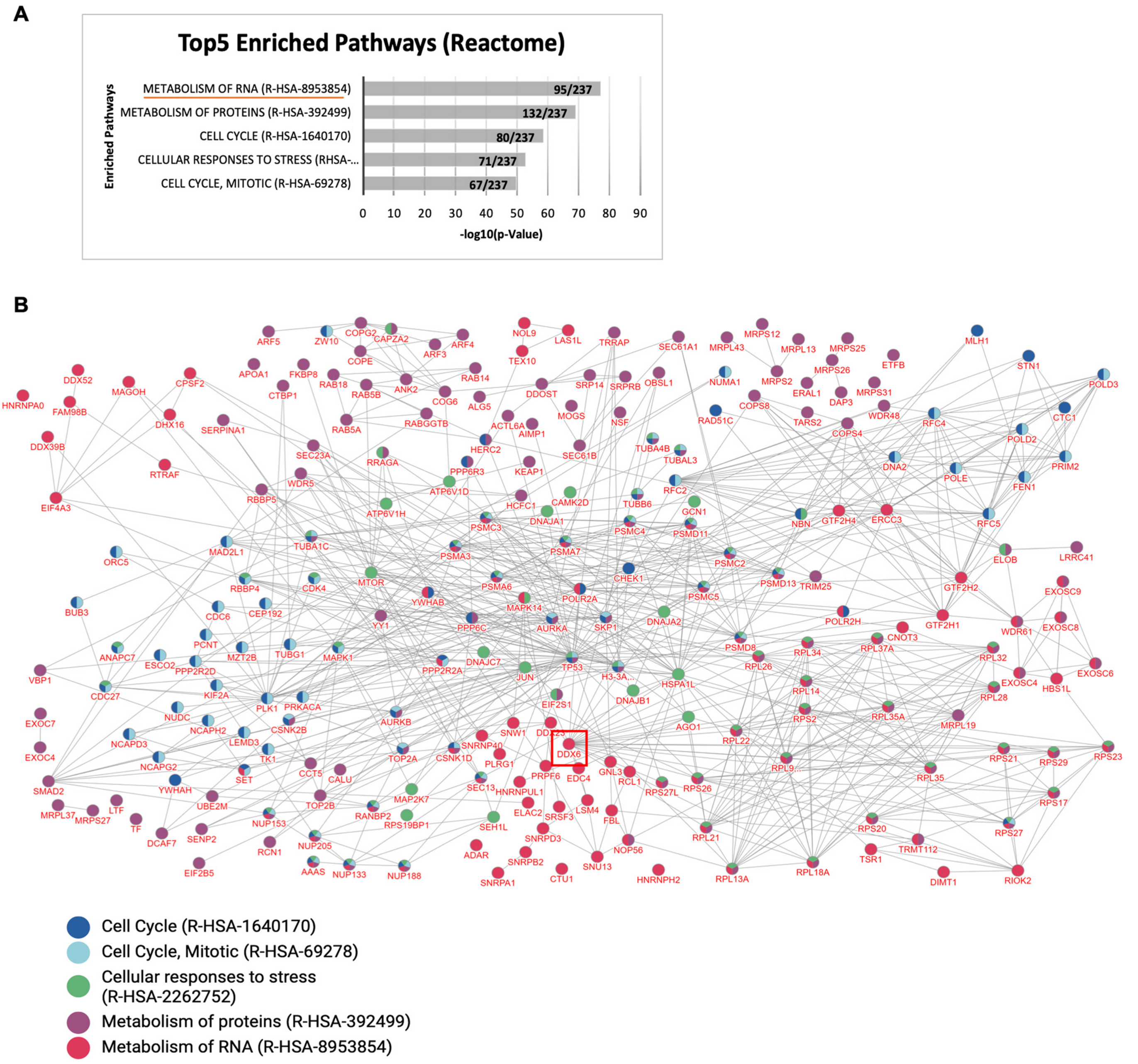

Figure EV2. Praja2 interacts with components of metabolism of RNA pathway.

(**A**) Barplot showing the Top5 most enriched REACTOME terms derived from the analysis of praja2-PPI network. (**B**) Protein–protein interaction network of components involved in cell cycle, response to stress and metabolism, which was generated using the inBio Discover web tool (no network expansion) querying the Reactome database for functional enrichment. The interactions existing between proteins involved in cell cycle (blue nodes), cell cycle, mitotic (cyan nodes), cellular responses to stress (green nodes), metabolism of proteins (purple nodes) and metabolism of RNA (magenta nodes) are shown ($n = 237$ proteins, $n = 762$ interactions).

 © The Author(s)

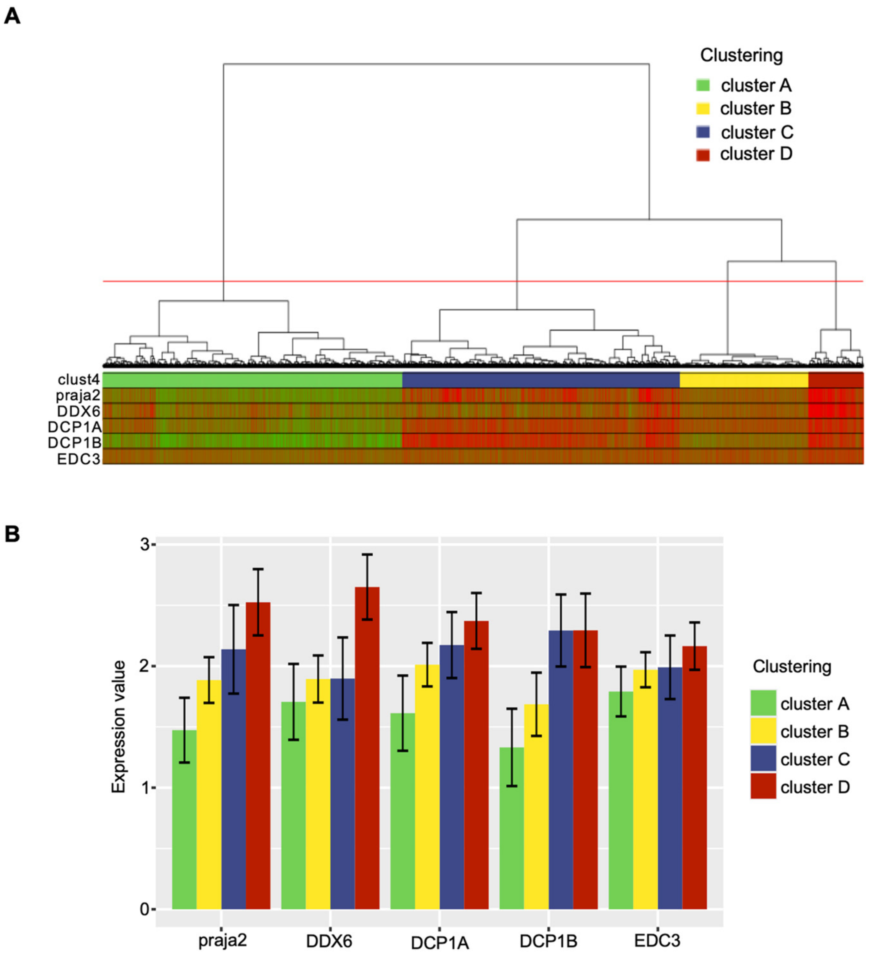

Figure EV3. Praja2 is a potential regulator of mRNA translation.

(**A**) Unsupervised hierarchical clustering of 20.000 cells from IDH wild-type GBM donor 701 (GSM3719277). The first annotation bar is the primary statistical clustering, while other bars show expression levels of the indicated genes (green= low expression, red= high expression). (**B**) Gene expression level across clusters. The bar graphs show each cluster's mean of gene expression levels of praja2, DDX6, DCP1A, DCP1B, and EDC3. Data from 20000 cells were used. Values represent the mean ± SD.

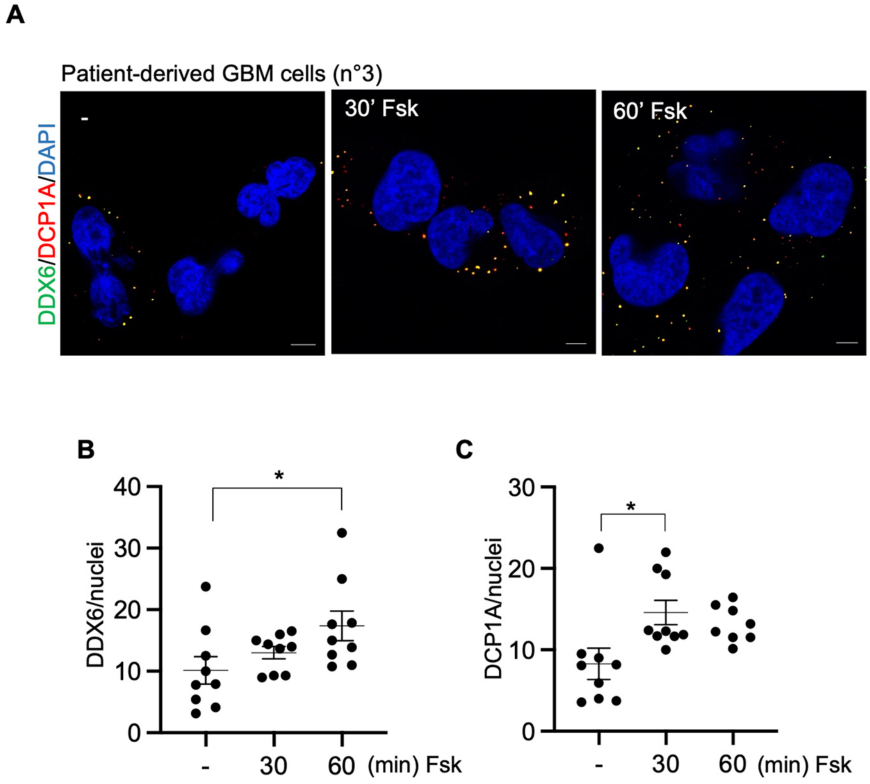

Figure EV4. cAMP induces P-body formation.

(**A**) GBM cells derived from patient 3 were treated 30 and 60 min with Forskolin (10 µM). Cells were fixed and immunostained with anti-DDX6, anti-DCP1A and DAPI. Scale bar: 5 µm. (**B**, **C**) Quantitative analysis of three biological independent experiments, mean ± SEM is indicated. *t* test $*P < 0{,}05$ (**B**, $P = 0.0413$; **C**, $P = 0.0213$). Source data are available online for this figure.

© The Author(s)

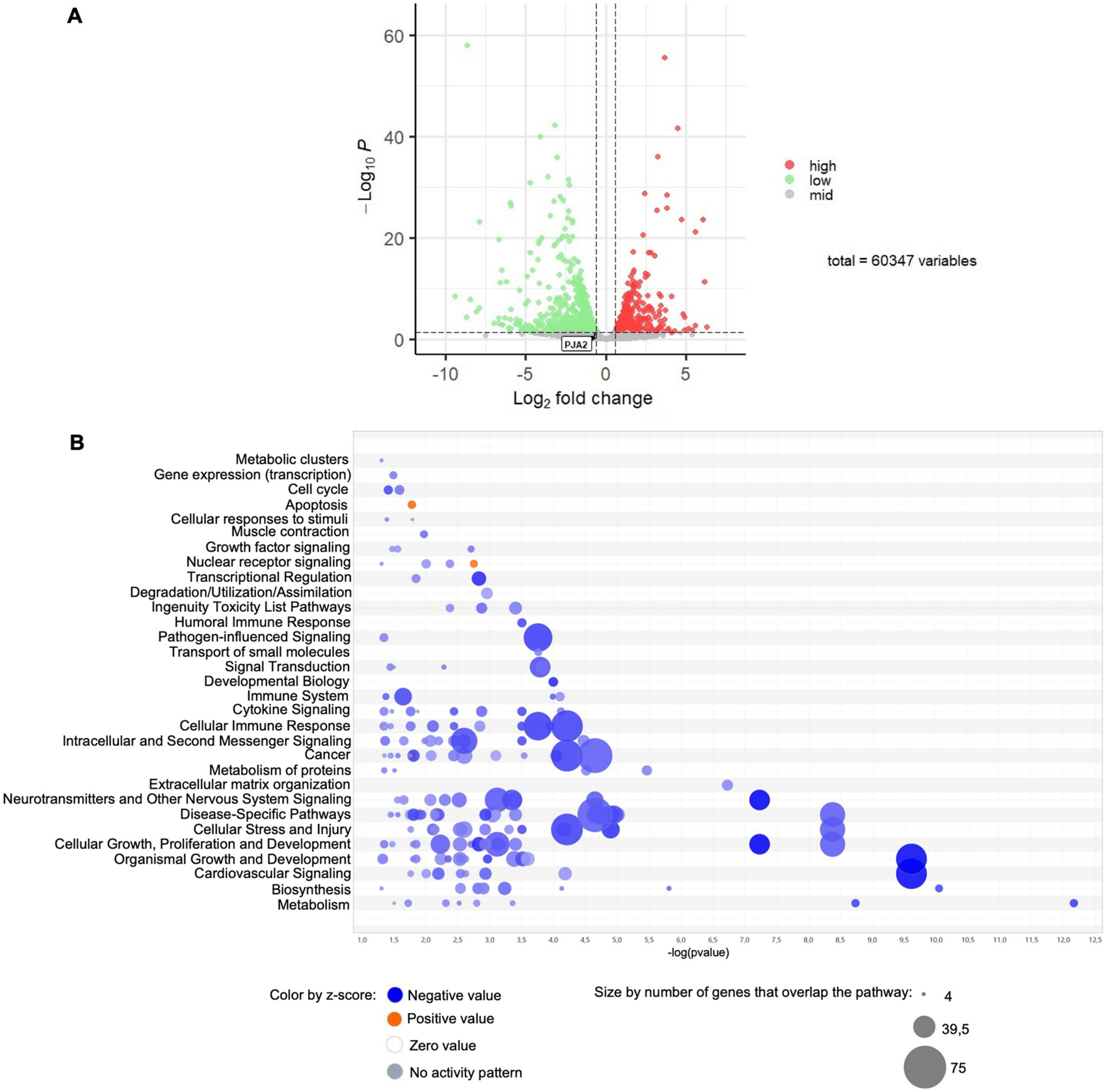

Figure EV5. Praja2 regulates transcriptome of GBM cells.

(**A**) Volcano plot showing log2FC and *P* value adjusted distribution of all genes, in particular down and upregulated differentially expressed (DE) genes comparing praja2KO versus WT cells are presented in green and red, respectively ($n = 3$ independent biological replicates). The statistical analysis was performed with Wald test of Deseq2. (**B**) Enrichment results of Ingenuity Pathways Analysis on DE genes, the size of the bubble indicates the number of enriched genes for each term. Statistical analysis performed with Fisher's Exact Test.
